# Optimized low‐dose combinatorial drug treatment boosts selectivity and efficacy of colorectal carcinoma treatment

**DOI:** 10.1002/1878-0261.12797

**Published:** 2020-10-05

**Authors:** Marloes Zoetemelk, George M. Ramzy, Magdalena Rausch, Thibaud Koessler, Judy R. van Beijnum, Andrea Weiss, Valentin Mieville, Sander R. Piersma, Richard R. de Haas, Céline Delucinge‐Vivier, Axel Andres, Christian Toso, Alexander A. Henneman, Simone Ragusa, Tatiana V. Petrova, Mylène Docquier, Thomas A. McKee, Connie R. Jimenez, Youssef Daali, Arjan W. Griffioen, Laura Rubbia‐Brandt, Pierre‐Yves Dietrich, Patrycja Nowak‐Sliwinska

**Affiliations:** ^1^ Molecular Pharmacology Group School of Pharmaceutical Sciences University of Geneva Switzerland; ^2^ Institute of Pharmaceutical Sciences of Western Switzerland University of Geneva Switzerland; ^3^ Translational Research Center in Oncohaematology Geneva Switzerland; ^4^ Department of Oncology Geneva University Hospitals and Faculty of Medicine Switzerland; ^5^ Angiogenesis Laboratory Department of Medical Oncology Cancer Center Amsterdam Amsterdam UMC‐location VUmc VU University Amsterdam The Netherlands; ^6^ Department of Medical Oncology Cancer Center Amsterdam Amsterdam UMC Vrije Universiteit Amsterdam The Netherlands; ^7^ OncoProteomics Laboratory Cancer Center Amsterdam Amsterdam UMC Vrije Universiteit Amsterdam The Netherlands; ^8^ iGE3 Genomics Platform University of Geneva Switzerland; ^9^ Translational Department of Digestive and Transplant Surgery Geneva University Hospitals and Faculty of Medicine Switzerland; ^10^ Hepato‐Pancreato‐Biliary Centre Geneva University Hospitals and Faculty of Medicine Switzerland; ^11^ Department of Oncology University of Lausanne Switzerland; ^12^ Ludwig Institute for Cancer Research Lausanne Switzerland; ^13^ Department of Genetics & Evolution University of Geneva Switzerland; ^14^ Division of Clinical Pathology Diagnostic Department University Hospitals of Geneva (HUG) Switzerland; ^15^ Division of Clinical Pharmacology and Toxicology Department of Anaesthesiology Intensive Care and Emergency Medicine Geneva University Hospitals Pharmacology Switzerland

**Keywords:** colorectal carcinoma, combination treatment, drug–drug interactions, drug–target interactions, phosphoproteomics, synergy

## Abstract

The current standard of care for colorectal cancer (CRC) is a combination of chemotherapeutics, often supplemented with targeted biological drugs. An urgent need exists for improved drug efficacy and minimized side effects, especially at late‐stage disease. We employed the phenotypically driven therapeutically guided multidrug optimization (TGMO) technology to identify optimized drug combinations (ODCs) in CRC. We identified low‐dose synergistic and selective ODCs for a panel of six human CRC cell lines also active in heterotypic 3D co‐culture models. Transcriptome sequencing and phosphoproteome analyses showed that the mechanisms of action of these ODCs converged toward MAP kinase signaling and cell cycle inhibition. Two cell‐specific ODCs were translated to *in vivo* mouse models. The ODCs reduced tumor growth by ~80%, outperforming standard chemotherapy (FOLFOX). No toxicity was observed for the ODCs, while significant side effects were induced in the group treated with FOLFOX therapy. Identified ODCs demonstrated significantly enhanced bioavailability of the individual components. Finally, ODCs were also active in primary cells from CRC patient tumor tissues. Taken together, we show that the TGMO technology efficiently identifies selective and potent low‐dose drug combinations, optimized regardless of tumor mutation status, outperforming conventional chemotherapy.

## Graphical abstract

Therapeutically guided multidrug optimization (TGMO) technology, composed of experimental testing and *in silico* modelling, allowed identification of synergistic and selective low‐dose optimized drug combinations (ODCs) active in multiple colorectal carcinoma models. The mechanisms of action of the ODCs was established using transcriptome sequencing and phosphoproteomic analyses. Our results indicate that simultaneous multitarget inhibition of important deregulated pathways has strong therapeutic potential and translational value between tumor types.

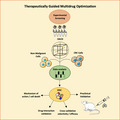

**Abbreviations**
ODC: optimized drug combination
PCL: plasma concentration limit
TGMO: therapeutically guided multidrug optimization
TW: therapeutic window

## 1 Introduction

Colorectal carcinoma (CRC) is among the most common cancers worldwide, and combination chemotherapy is the mainstay of treatment. Although life expectancy for CRC patients is improved by this therapy, the patients experience side effects and acquired drug resistance [1]. Currently, recommended first‐line regimens for advanced CRC include chemotherapy with 5‐fluorouracil/leucovorin/oxaliplatin (FOLFOX) or 5‐fluorouracil/leucovorin/irinotecan (FOLFIRI) [2]. Multidrug chemotherapy for CRC treatment is often supported by the administration of bevacizumab (Avastin^®^, targeting VEGF), or either cetuximab (Erbitux^®^) or panitumumab (Vectibix^®^, targeting EGFR), both positively correlated with improved survival in KRAS^WT^ CRC [3, 4]. Furthermore, the multikinase inhibitor regorafenib (Stivarga^®^, targeting with highest affinity VEGFR1‐3 and platelet‐derived growth factor receptor β, PDGFRβ) is now accepted as a third‐line treatment with beneficial survival profiles and manageable toxicities [5]. Notably, 5% of patients with stage IV CRC presenting a dMMR or MSI‐H tumor‐mediating high mutation burdens and unique immunogenic profiles are now eligible for treatment with anti‐PD‐1 or anti‐PD‐L1 antibodies, the first targeted immunotherapies approved for the treatment of CRC [6]. However, for late‐stage patients with a refractory disease, no further options exist beyond the chemotherapy combinations and abovementioned single or supplemental targeted therapies, thereby with an estimated 9.2% mortality rate in 2018 CRC remains the fourth leading cause of cancer‐related deaths worldwide [7].

On a molecular level, activation of receptor tyrosine kinases (e.g., EGFR, VEGFR, FGFR, and PDGFR) stimulates MAPK and PI3K/Akt/mTOR pathway. These signaling pathways play key roles in normal cell homeostasis. The MAPK pathway has a major role in stimulating cell proliferation through a RAS/RAF/MEK/ERK cascade, while the PI3K/Akt/mTOR pathways regulate a myriad of cellular processes including cell proliferation, differentiation, metabolism, and survival. Oncogenic activation and deregulation of these pathways are mediated by mutations in KRAS and BRAF, or activation of WNT, MYC, and TGF‐β signaling driving proliferation, cell cycle deregulation, and altered immune and stromal interactions [8]. The use of targeted agents such as tyrosine kinase inhibitors (TKIs) to selectively inhibit oncogenically activated signaling pathways has shown great promise [9]. However, the use of these compounds even at the maximum tolerated doses frequently leads to tumor relapse, as well as severe side effects [10].

Although the development of targeted therapies, the use of repurposed drugs [11] and the introduction of personalized medicine will improve treatment outcomes [12]. It is expected that combination strategies hold the biggest promise. Combination therapies can overcome complications linked to side effects and induction of drug‐related resistance due to nonoverlapping mechanisms of actions [13, 14]. Moreover, multinode targeting combination therapy is considered to be an attractive approach to effectively inhibit key oncogenic signaling pathways [15]. The mutations and deregulations of signaling pathways are linked to the robustness and adaptability of complex biological systems that favor compensatory mechanisms, which tumors can take advantage of [16]. Targeting those (compensatory) signaling pathways at multiple levels can result in enhanced efficacy and therapeutic selectivity [13, 17].

In order to design an effective anti‐cancer therapy containing multiple drugs, it is necessary to consider nonlinear complex networks, which are most likely not fully characterized. Although others have previously attempted to address this challenge by different approaches, we now propose to use our validated TGMO technology [18]. This phenotypically driven platform allows rapid optimization of synergistic multidrug combinations applied at low doses, and it is based on an experimental screen with only a small fraction of all possible drug combinations and data modeling. Moreover, in this screen we introduced the use of doses based on reported plasma levels in humans and combined this with the evaluation of these doses in nonmalignant cell lines to maximize the therapeutic window and translational applicability.

Here, we report the identification of an improved treatment regimen for CRC. The *in vitro* cell‐specific ODCs were further validated in more complex heterotypic 3D models and successfully translated to *in vivo* tumor models as well as in freshly isolated metastatic CRC patient material. RNA‐sequencing and phosphoproteomic analysis revealed modulation of the MAP kinase pathway, cell cycle inhibition, and cell death induction as the main mechanisms of action of the ODCs. Therefore, our technology enables identification of ODCs that outperforms genome‐ and mutation‐based predictions of possible treatments.

## 2 Materials and methods

### 2.1 Cells and culture conditions

Human CRC and nonmalignant cells were obtained from ATCC or Public Health England with a corresponding authentication certificate. Human immortalized endothelial cells ECRF24 cells were generously donated by Prof. AW Griffioen (Angiogenesis Laboratory, UMC Amsterdam). The cells were cultured in a humidified incubator at 37°C and 5% CO_2_ in culture medium supplemented with 10% fetal bovine serum (S1810‐500, Biowest, Nuaillé, France) and 1% penicillin/streptomycin (4‐01F00‐H, BioConcept, Allschwil, Switzerland). Cells were monitored for mycoplasma contamination using the MycoAlert kit (LT07‐218, Lonza, Rockland, ME, USA).

2D cell cultures for drug combination optimization experiments were performed in flat‐bottom 96‐well plates (353072, Falcon, Durham, NC, USA), seeding 2,500 c/w DLD1, 5,000 c/w SW620, 2,500 c/w HCT116, 3,500 c/w LS174T, 2,500 c/w HT29, 10,000 c/w SW48, 3,000 c/w CCD18co, 3,000 c/w CCD841, and 5,000 c/w ECRF24 cells. For immunocytochemistry, staining cells were seeded in 24‐well plates (662160, Falcon) on 12‐mm round glasses at 12,000 for DLD1, 30,000 for SW620, 12,000 for HCT116, 20,000 for LS174T, 12,000 for HT29 and 60,000 for SW48 per well, for 2h/24h and 72h treatments, respectively. For flow cytometry and RNA‐sequencing experiments, cells were seeded in flat‐bottom 6‐well plates (353046, Falcon) at 200,000 and 80,000 for DLD1, 500,000 and 250,000 for SW620, 200,000 and 80,000 for HCT116, 250,000 and 125,000 for LS174T, 200,000 and 80,000 for HT29, and 800,000 and 300,000 for SW48 per well, for 2h/24h and 72h treatments, respectively. Culture media: RPMI‐1640 Glutamax medium (61870‐010, Gibco, Paisley, UK) for DLD1; DMEM Glutamax medium (31966‐021, Gibco) for SW620, HCT116, LS174T, HT29, SW48; EMEM medium (M2279‐500ML, Sigma‐Aldrich, St. Louis, MO, USA) additionally supplemented with 2 mM L‐Glutamine (25030024, Gibco) for CCD18co and CCD841CoN. ECRF24 cells were cultured on a 0.2% gelatin‐coated surface (G1393‐100ML, Sigma) in DMEM/RPMI 1:1.

3D cell cultures were performed in 96‐well U‐bottom low attachment plates (650970, Greiner Bio‐One, Frickenhausen, Germany) with CRC cells seeded 1:1 with CCD18co cells and 5% ECRF24 cells (500:500:50 cells). Culture media consisted of equal amounts of DMEM, RPMI and EMEM supplemented with 2.5% Matrigel^TM^ (354254, Corning, Bedford, MA, USA) [19]. The 3D‐CCs were treated with drugs on day 2.

### 2.2 Patient material

The study methodologies with the patient‐derived CRC metastasis cell cultures were approved by the Swiss Ethics Committee on research involving humans (2017‐00364). The study methodologies conformed to the standards set by the Declaration of Helsinki. The experiments were undertaken with the understanding and written consent of each subject.

The patient‐derived CRC metastasis tissues were transported in DMEM‐F12 (31330038, Gibco) and 1x Primocin (ant‐pm‐1, Invivogen, Toulouse, France), and processed within 1h of resection. Tissues were thrice washed, weighted for reference, and mechanically dissociated into 1–3 mm^3^ cubes with a surgical blade in a small glass petri dish in 1 mL digestion medium. Digestion medium consisted of DMEM/F12 with 1x Primocin, 100 µg/mL DNAse I (10104159001, Roche, Mannheim, Germany), and 5 mM CaCl2 (C7902), Collegenase IV (C1889) and TES (T1375) from Sigma‐Aldrich. Afterward, enzymatic digestion was continued with 10 mL/gr tissue using GentleMACSTM technology using violet C tubes (130‐096‐334) from Miltenyi Biotec (Bergisch Gladbach, Germany), for 1h at 37°C with protocol 37C_TDK‐01. Cell suspensions were filtered with a 100‐µm nylon EASYstrainer (7.542 000, Greiner) and washed twice with HBSS‐MgCl2/‐CaCl2 with phenol red (14170088, Gibco) to stop digestion, and cells were collected through centrifugation. Cells were resuspended, and cell viability and cell counts were obtained through fluorescent live‐death orange acrylamide and propidium iodide staining (LGBD10012, Vita Scientific, College Park, MD, USA) using the LUNA^TM^ automated cell counter (Logos Biosystems, Villeneuve d’Ascq, France).

Primary patient cultures were established in 96‐well U‐bottom low attachment plates (650970, Greiner), seeding at 20.000 c/w in supplemented DMEM/F12 culture medium with HEPES and L‐glutamine. Supplements: 1x primocin; 1x MEM NEAA (11140050), 1x insulin‐transferrin‐selenium (41400045) and 1x B27 (17504044) from Gibco; 0.15% D‐glucose (G8270‐1000), 1 mM N‐acetyl‐L‐cysteine (A9165‐5G), 10 mM nicotinamide (N0636‐100G), 2 µg/mL hydrocortisone (H0888‐1G), and 4 µg/mL heparin (H3149) from Sigma‐Aldrich. Cells were imaged using the BioTek Cytation 3 imaging reader with corresponding Gen5 Image software version 3.04.

### 2.3 Drugs and treatments

Drugs were dissolved in DMSO and stored at −80, and aliquots were thawn prior to each use. Drugs were dissolved at concentrations resulting in *in vitro* experimental conditions 0.15% DMSO maximum in the culture medium. Drug stocks: 20 mg/mL regorafenib (R‐8024), 15 mg/mL erlotinib (E‐4007), 20 mg/mL vemurafenib (V‐2800), and 1 mg/mL BEZ‐325 (N‐4288) from LC laboratories (Woburn, MA, USA); 20 mg/mL selumetinib (HY‐50706), 10 mg/mL AZD‐4547 (HY‐13330, 10 mg/mL GDC‐0994 (HY‐15947), 20 mg/mL folinic acid (HY‐17556), and 5 mg/mL oxaliplatin (HY‐17371) from MedChemExpress (Monmouth Junction, NJ, USA); 10 mg/mL vatalanib (S1101) and 10 mg/mL crenolanib (S2730) from Selleck Chemicals (Houston, TX, USA); 10 mg/mL 5‐fluorouracil (F6627) from Sigma‐Aldrich; 4 mg/mL Zaltrap^®^ from Sanofi (Paris, France). Single drugs or premixed drug combinations were incubated with the 2D cell cultures for 24 hours or 72 hours, applied at day 1 postseeding and with the 3D cell cultures for 72 hours or 72 hours + 48 hours, applied at day 2 or day 2 + day 5 postseeding [19, 20]. The 48‐hour retreatment was performed by adding another volume of media containing a 1x concentration of drugs to not affect the final concentration in the wells.

### 2.4 Metabolic ATP activity assays

Drug treatment activity in 2D and 3D cultures was measured using the CellTiter‐Glo^®^ cell metabolic activity (ATP) assays (G7572 and G9683, Promega, Madison, WI, USA), according to the manufacturer`s instructions. Assay bioluminescence was detected using the BioTek Cytation 3 and corresponding Gen5 Image software version 3.04 at standard settings.

### 2.5 Therapeutically guided multidrug optimization method

The therapeutically guided multidrug optimization (TGMO) method [18] was used for (i) the identification of drug–drug interactions between 11 drugs and multiple doses and (ii) the selection of an optimal drug combination, see Fig. S1.

First, the experimental data points, that is, the drug combinations, are based on orthogonal array composite design (OACD) matrices, each specifically designed for the optimal information acquisition from experimental screening of drug combinations performed in *Search 1* (11 drugs), *Search 2* (7 drugs), or *Search 3* (4 drugs) [21, 22]. In specific, the first part of the matrix is a two‐level fractional factorial design, exposing linear effects over a large search space resulting in estimated regression coefficients of single‐drug and two‐drug interactions. The second part, a three‐level orthogonal array design, investigates both linear and quadratic effects and informs on the nonlinear response surface over multiple doses. The resulting OACD matrix is a resolution IV matrix [23] and is ideal for defining the most influential variables within a group or system by estimating each variable's main effect.

In practice, the TGMO is initiated by defining the two dose‐level inputs for the screening. The drug input is optimal at low doses: ED_20_ (highest dose, aliased as dose 2) and half of this dose or ED_10_ (lowest dose, alias dose 1), in addition to the use of no dose (dose 0). The low doses are selected in order for the regression analysis to accurately provide estimates on the effect of the variables and explore drug response surfaces, the latter is also an important influencer in drug–drug interactions. Thus, the first step is to perform drug dose–response curves and define the drug dose input for each of the 11 drugs. Note, to enhance clinical relevance, only clinically relevant doses were selected (see plasma concentration limit below). In some cases, the drug input is therefore below the ED_20_. Consequently, while more realistic, it limits the full potential of the variables to be identified as interactors to only the strongest of drug interactions.

The next step is the drug combination screening in *Search 1* (11 drugs in 155 combinations) according to the OACD resolution IV matrix and performed in the CRC and the nonmalignant CCD841CoN cell simultaneously. The resulting output of the drug combination activity was measured in cell metabolic activity (ATP levels, % CTRL) to represent cell viability and consisted of the average of technical triplicate values. The experimental data points are then used for step‐wise second‐order linear regression analysis by Matlab^®^. This model mathematically describes the relationship between the drug combination input and output activity of each possible two‐drug combination. In this equation, the activity is the sum of β_0_, β_i,_ β_ii,_ and β_ij_, which represent the intercept and the linear, quadratic, and bilinear (or interaction) terms, respectively. x_i_ and x_j_ are the independent variables (the drugs), and ε is an error term with a mean equal to zero [22].

The second‐order linear regression analysis generates a model with predicted effects for the variables represented in estimated regression coefficients. These coefficients describe (i) the contribution of each drug individually to the drug combination, referred to as single‐drug first‐order terms; (ii) the identified drug:drug interactions and their overall effect on the activity of the drug combination, referred to as the two‐drug interaction terms; and (iii) the response surface of a drug independently and as part of an interacting drug pair, referred to as single‐drug second‐order terms (drug^2^). The later one specifically defines the effect of the drug on the drug combination activity over the different dose levels. Graphically, for (i) and (ii), negative regression coefficients signify inhibitory efficacy or synergistic activity, and positive regression coefficients signify stimulatory efficacy or antagonistic activity. For (iii), positive and negative regression coefficients depict stable effect over the dose range tested and dose‐dependent contributions, respectively. The generated models guide drug selection and elimination, and through consecutive rounds of screening (*Search 1‐3*), biological noise is narrowed and the most strong and robust drug interactions define the final drug combination selection.

Besides modeling the drug combination activity on CRC cells, the screening is simultaneously performed on nonmalignant colon epithelial CCD841CoN cells and the difference between the two, termed the therapeutic window (TW), is used as a secondary model to visualize selectivity of drug combination activity. Consequently, the most optimal effect is depicted as opposite regression coefficients for CRC efficacy (negative) and the TW (positive).

To confirm the selection of the step‐wise second‐order linear regression model, the analysis includes an ANOVA lack of fit test which should show a lack of significance to confirm correct model selection. Second‐order linear regression models are generally sufficient. Higher‐order three‐drug interactions have mostly a negligible effect on the overall combination activity [24]. Exceptionally, a third‐order linear regression model of a 3 or 4 drug combination could be needed to expose underlying three‐drug interactions and is indicated by significance of the ANOVA lack of fit test.

To determine the predictive value of the models based on the experimental data, several model analyses are conducted (Fig. S2). First, the coefficient of multiple determination (R^2^) evaluates the observed vs. fitted accuracy and co‐dependence of variables. The higher the R^2^, the lower the exogeneity and lack of multicolinearity, and the higher the model accuracy and correlation between experimental and fitted data. Second, Q‐Q plots visualize the independence of errors, residual analysis of the observed vs. fitted data points assesses the constancy of variance, and residual histograms appraise if the variance is normally distributed. Third, Cook’s distance analysis identifies influential outliers in the set of predictor variables. The larger the leverage of a data point, the higher the Cook’s distance and the more likely it negatively impacts the model. Therefore, several models are acquired from each dataset: a model without outliers, a model with the maximum outlier removed and a model with the outliers above 3‐fold the mean Cook’s distance removed. In practice, differential interactions appear between the models. In some cases, the removal of outliers might give more accurate drug interaction predictions, but care must be taken to not create bias and incorrect variables by removing too many outliers. Importantly, those regression coefficient terms not affected by outlier removal are the most reliable and are used for guiding drug selection and elimination. To summarize, the models with the highest integrity or robustness are those with the highest R^2^, have a good fit between observed and fitted values, and are nearly unchanged after outlier removal.

Important to note is that biological variation can result in inaccurate estimations of drug variables, mostly restricted to drugs with a lack of or low single‐drug activity. To counteract this, experiments are performed in triplicate. Moreover, the data are modeled for each dataset separately and for all datasets together in the combined model (the final model graphically presented). The most reliable interactions are those that appear in all models and have the highest significance.

Nonempirical testing can result in inaccurate effect predictions of variables by ‘shielding’ of the effect of variables within the effect of other variables or two‐factor effects. To counteract this, the resolution IV OACD matrix provides cross‐validation between the two parts of first‐order and second‐order within the matrix design. To further improve the accurate identification of the most optimal drug combinations, the screening is performed in sequential rounds. Each round feeds the selection of the most interesting and active drugs and the elimination of the most inactive and antagonistic drugs for the next round. The most robust interactions are those that appear in multiple rounds, including the final model. In this case, screening progresses from *Search 1* to *Search 3*, finally resulting in the selection of the most optimal combination.

### 2.6 Plasma concentration limit

To select for clinically relevant doses, the clinically attainable drug concentration measured in the blood plasma of patients after treatment is used to calculate the concentration limit for *in vitro* experiments. First, drug pharmacokinetic studies in patients yield information on the drug concentration over the first 24h, specifically, the C_max_ and area under the curve (AUC_0–24h_). The average drug concentration is calculated from the AUC_0–24h_ and is implemented as the plasma concentration limit (PCL) which drug dose selection must remain below for *in vitro* experiments. If a drug is FDA approved, the AUC_0–24h_ selected is the one corresponding to the recommended dose in clinical practice. If a drug is in clinical trials, the AUC_0–24h_ selected is the one corresponding to the maximum tolerated dose in early stage clinical trials. Note, experiments were conducted with the most current PCL calculated from the AUC_0–24h_ information available at the start of experiments (December 2017), see Table S4.

### 2.7 Flow cytometry and immunofluorescence stainings

Flow cytometry analysis of cell cycle distribution was performed with propidium iodide staining to measure cell cycle distribution and the fraction of dead cells, according to a standard protocol. Attached and floating cells (1–5 × 10^6^) were harvested from 6‐well plates, were washed once with PBS, fixated with 70% EtOH for 2h, washed once with PBS and stained for 30 minutes at RT with FxCycle^TM^ propidium iodide/RNAse staining solution (F10797, Invitrogen, Carlsbad, CA, USA). Emission at 617 nm was detected with the BL2 channel on the Attune NxT flow cytometer (Life Technologies, Carlsbad, CA, USA), using corresponding software v.2.5.

### 2.8 Immunofluorescence stainings

Immunofluorescence staining was performed on cells cultured in 24‐well plates fixated on 12‐mm round glasses using cytoskeleton F‐actin and nuclear DAPI staining. Briefly, cells were fixed with 2% formaldehyde for 10 minutes at RT, washed twice with PBS, and permeabilized with 0.1% Triton‐X in PBS for 15 minutes. Blocking of unspecific binding sites was done with 1% BSA solution for 20 minutes, and cells were stained for F‐actin with Phalloidin Flash‐488 (424201, Biolegend, San Diego, CA, USA) for 1h at room temperature (RT). Glasses were washed twice with PBS, and stained for the nucleus with DAPI (A4099,0005, PanReac AppliChem, Darmstadt, Germany) and submerged in PBS. Fluorescence images were obtained using the BioTek Cytation 3 imaging reader with corresponding Gen5 Image software version 3.04. Imaging was performed with bright field or with the DAPI, GFP, and Texas Red filter cubes using the 4x and 10x objectives. Images were obtained using the BioTek Cytation 3 imaging reader with corresponding Gen5 Image software version 3.04. Imaging was performed with bright field or with the DAPI, GFP and Texas Red filter cubes using the 4x and 10x objectives.

### 2.9 mRNA transcriptome and analysis

RNA extraction was performed with the RNA easy® Plus kit (74134, Qiagen, Hilden, Germany) according to the manufacturer’s instructions. The RNA quality control, library preparation using TruSeqHT Stranded mRNA (Illumina), and sequencing on an Illumina HiSeq 4000 System using 100‐bp single‐end reads protocol were performed. Quality control was performed with FastQC v.0.11.5. Reads were mapped to the human genome (UCSC hg38) using STAR v.2.5.3a software with average alignment around 92%. Biological quality control was done with PicardTools v.2.9.0. Raw counts were obtained using HTSeq v.0.9.1. Normalization and differential expression analysis were performed with the R/Bioconductor package edgeR v.3.24.3, and statistical significance was assessed with a general linear model, negative binomial distribution, and quasi‐likelihood F test. Genes with a fold change> 2 and p‐value < 0.05 (with a false discovery rate of 5%) were considered differentially expressed. The RNA‐Seq data have been deposited in the NCBI Gene Expression Omnibus (www.ncbi.nlm.nih.gov/geo) and are accessible through GEO Series accession GSE142340.

Gene ontology enrichment analysis on genes downregulated after ODC treatment was performed in Enrichr (http://amp.pharm.mssm.edu/Enrichr ) for biological process. For network analysis, differentially expressed genes (downregulated after ODC) were analyzed in STRING (string‐db.org), incorporating a maximum of 10 first‐order interacting proteins. Networks, excluding unconnected nodes, were visualized in Cytoscape (v3.7.1).

### 2.10 Phosphoproteomics

CRC cells were cultured to near‐confluence and were exposed to ODCs or vehicle solutions for 2 hours. Cells were lysed in the presence of phosphatase inhibitors and processed and INKA analysis was performed, as described previously [25]. INKA scores and associated networks were presented with the outline of the top 20 active kinases (i.e., highest ranking INKA scores) of untreated samples are overlaid with the scores after ODC treatment. For interpretation and visualization of differential phosphoprotein expression, normalized count data were used, and selections were made based on> 1.5 FC in ODC‐ vs. CTRL‐treated samples, for both replicates, and a summed count value over the replicates of> 5 to include only proteins with a consistent level of expression. Protein–protein interactions were analyzed using STRING, and visualized with Cytoscape (v3.7.1), leaving out unconnected nodes. Nodes were color‐coded proportional to expression fold change. Pathway enrichment analysis (WikiPathways) was done using Enrichr on the ODC‐treated downregulated phosphogenes. Proteomics data have been deposited in ProteomeXchange via the PRIDE repository with accession number PXD016604 and Tables S14–19 at Zenodo (https://doi.org/10.5281/zenodo.3580018).

*In silico* drug–target interaction analysis was performed. Drug–target interactions were analyzed and visualized using data previously generated by Klaeger *et al*., based on cell‐free assays [26] (www.proteomicsdb.org). In effect, drugs were selected at their ODC concentration, and the percentage of effective inhibition was set to 50%. Subsequently, targeted kinases were analyzed for protein–protein interactions in STRING, either with or without the inclusion of a maximum of 10 first‐ and second‐order interacting proteins.

### 2.11 Subcutaneous and orthotopic *in vivo* tumor models

All procedures including animal use were performed in accordance with the Institutional Ethical Committee of Animal Care in Geneva and the Swiss Cantonal Veterinary Office (Authorization number GE‐136‐19). Briefly, female and male Swiss nu/nu mice aged 6–8 weeks were obtained from Charles River (Écully, France). For subcutaneous xenografts mice were inoculated in the left flank with 5x10^6^ DLD1 or SW620 cells suspended in 100 µL of cell culture medium, supplemented with 1% FBS. Treatment was initiated when palpable tumors had formed (approximately 30 mm^3^) on day 4 for DLD1 and day 5 for the SW620 model.

The orthotopic model was conducted as previously described [27, 28]. Mice were anesthetized using 3–5% inhalation isoflurane, and once asleep, the skin was cleaned with iodine and ethanol. An incision was made in the skin and peritoneum of ± 1 cm and the cecum exteriorized unto sterile gauze as previously described. 2 x 10^6^ DLD1 luciferase‐expressing cells were resuspended in nonsupplemented medium and mixed with 33% Matrigel to a total volume of 25 µL.

Cells were inoculated using a Hamilton syringe (074421, Ham‐7644.01, 805RN 50 µL; 036078, RN 803‐02). For cell inoculation, the cecum was kept moist and flattened to facilitate easy entry into the cecum wall. After the protrusion containing the cell suspension was confirmed, the needle was retracted and the cecum was thoroughly flushed with PBS to prevent cell reflux. After returning the cecum to the gut, the peritoneum was closed using running interrupted sutures, and the skin was closed with wound clips. The surgery area was topically treated with betadine.

Mice received 200–300 mg/kg Dafalgan in the drinking water 24h pre‐operatively until 72h postoperatively. 15 min before anesthesia and surgery mice were injected with 0.1 mg/kg Buprenorphine i.p. to reduce pain and stress. Postsurgery, mice received up to two 0.1 mg/kg Buprenorphine injections every 6h. Tumor growth was followed based on bioluminescence measurements after injecting of 0.15 mg/gram mouse using 45 mg/mL D‐luciferin (BC218, SYNCHEM) and imaged ± 30 min postinjection. Tumor size was measured daily and tumor volume was calculated using the formula ‘smallest diameter^2^ + biggest diameter/ 2’.

Treatment was initiated once tumor growth was confirmed with bioluminescence measurements. Drugs were administered through oral gavage based on average body weight of the males (100 µL/mouse) and females (80 µL/mouse) at various concentrations, see Tables S10 and S11.

Adequate steps were taken to ensure that animals did not suffer at any stage of experiment by daily weighing and behavior scoring to monitor health. Mice were euthanized according to the Swiss regulations at endpoints of 1,000 mm^3^ tumor volume. At the last day of experiment, tumors were resected, measured, and weighted.

### 2.12 *In vivo* drug combination treatment

For drug treatment, multicomponent solvents were prepared in stock solutions of 50 mL and were used for step‐wise dissolving of the drugs to a total of 100% solution A (regorafenib, erlotinib, selumetinib) or B (vemurafenib, GDC‐0994). Solution A: step (1) 30% PEG 400 + 5% propylene glycol + 0.5% Tween 80 and (2) 64.5% sterile H_2_O. Solution B: step (1) 10% DMSO, (2) 30% PEG 300 + 5% Tween 80 and (3) 55% sterile H_2_O. Vemurafenib was prepared 2x concentrated (half or three/sixth of total solution), and the remaining drugs were prepared 6x concentrated (one/sixth of total solution), after which drugs were diluted to prepare 1x drug concentrations for the ODC or single drugs using the other drugs or complete solvent solutions without drugs for lower concentrations. FOLFOX was administered as 6 mg/kg oxaliplatin two hours before administration of 90 mg/kg leucovorin and 50 mg/kg (DLD1) or 25 mg/kg (SW620) 5‐fluorouracil at day 1 and a repeat of 5‐fluorouracil dosing on day 2.

### 2.13 Cachexia analysis

For DLD1 tumor‐bearing mice, the following numbers of mice hearts were included in the analysis: 6 in the CTRL group (3 males and 3 females), 5 in the ODC‐treated group (3 males and 2 females), and 3 in the FOLFOX‐treated group (2 males and 1 female). For SW620‐bearing mice, we included the following number of mice hearts: 3 in the CTRL group (2 males and 1 female), 5 in the ODC‐treated group (2 males and 3 females), and 4 in the FOLFOX‐treated (3 males and 1 female). The analysis was performed by calculating the % of heart weight per overall mouse weight.

### 2.14 Pharmacokinetics study

The pharmacokinetic (PK) study was performed using the dried blood spot method. Mice were treated with CTRL, ODCs, and corresponding monotherapies as described above. To determine the disposition in blood in the first 24h, blood samples (5 µL/mouse) were collected from the tail vein (n = 4 mice) at 2h, 4h, 8h, and 24h time points and transferred onto paper (a filter paper 903 protein saver card from Whatman (MA, USA)). A disk with the entire spot was punched from the paper card and transferred to the HPLC vial tube. Hundred µL of MeOH containing a mix of internal standards was added, and the tube was vortex‐mixed for about 1 min before injecting 10 µL into the LC‐MS/MS system. The LC‐MS/MS system consisted of an API 4,000 triple quadrupole mass spectrometer (AB sciex, Concord, ON, Canada) controlled by analyst 1.6.1 software. The mass spectrometer was operated in the multiple reaction monitoring (MRM) mode with positive electrospray ionization. The instrument was coupled with an Agilent series 1100 (Waldbronn, Germany) LC system. Chromatography was performed on a Phenomenex Kinetex C18 analytical column (50 mm x 2.1 mm, 2.6 um; Torrance, CA, USA). The flow rate was 0.5 mL/min using gradient elution conditions. The method was fully validated before application to this pharmacokinetic study.

At the experimental endpoint, plasma and tumors were collected 1h post‐treatment and part of the tumor tissue was used to determine the intratumor drug concentrations. Plasma and tumor samples were analyzed using the same LC‐MS/MS method used for drug determination in DBS. Before analysis, tumor tissues were homogenized in a 1 mL mixture of water/acetonitrile (30/70). Hundred uL of homogenates were transferred into new tubes and 50 µL of acetonitrile, as well as 50 µL of a mix of internal standards in MeOH were added. Tubes were vortexed and centrifuged for 10 min at 9,000 rpm. The supernatant (100 µL) was evaporated under nitrogen and reconstituted in 100 µL of acetonitrile with 0.1% formic acid/water with 0.1% formic acid (30/70). 10 µL was then injected into the LC‐MS/MS system. Plasma samples were spotted onto the same paper used for whole blood analysis and were extracted and analyzed using the same methods as used for DBS.

### 2.15 Immunohistochemistry and immunofluorescence

DLD1 and SW620 tumors resected from Swiss nu/nu mice were fixed in 4% formaldehyde and paraffin‐embedded. Cross sections were used for the following stainings: hematoxylin and eosin (H&E), Ki67, and CD31, using standard protocols. For both immunohistochemistry (IHC) and immunofluorescence (IF), cross sections were de‐paraffinized with Néo‐Clear (64741‐65‐7, Sigma‐Aldrich) and EtOH and heat‐induced epitope retrieval was conducted using Citrate buffer at 100°C for 20 minutes. Sections were washed twice with 1x PBS, and once with 0.2% Triton‐X in PBS (PBST), a boundary was marked using a hydrophobic pen (H‐4000, Vectorlabs, Burlingame, CA, USA) and slides were placed in a humidified chamber. For IHC, endogenous peroxidase activity was blocked with a 0.3% peroxidase H_2_O_2_ blocking solution (S2023, DAKO, Agilent, Santa Clara, CA, USA) for 5 minutes at RT after which the sections were washed once. For both IHC and IF, blocking of unspecific binding sides was done with 1% BSA solution for 20 minutes at RT. Sections received primary antibody solution diluted in blocking buffer for overnight incubation in the humidified chamber at 4°C and were washed twice with PBST and once with 1x PBS. Sections were stained with secondary antibodies diluted in blocking buffer and incubated for 1h in the humidified chamber at RT and washed twice with PBST and once with 1x PBS. For IC, the sections were directly mounted with Vectashield^®^ hard fluorescence mounting medium containing DAPI to stain the nucleus (H‐1500, Vectorlabs, Burlingame, CA, USA). For IHC, sections were incubated with DAB substrate (ab64238, Abcam, Cambridge, UK) for 5 minutes at RT, counterstained with hematoxylin, and mounted with Vectashield^®^ hard fluorescent mounting medium (H‐1000, Vectorlabs). Antibodies: rabbit‐anti‐Ki67 (9027S, Cell signaling, Danvers, MA, USA), rat‐anti‐CD31 (DIA‐310, Dianova GmbH, Hamburg, Germany), goat‐anti‐rat IgG H&L biotin (ab6844, Abcam). Bright field and fluorescence images were obtained using the Axio Scan.Z1, 20x objective, using ZEN lite corresponding software at standard settings.

### 2.16 Statistical analysis

All data are presented as the mean of minimally two independent experiments with corresponding error bars of standard deviation (SD) or the standard error of the mean (SEM), as indicated in the figure legends. Data analysis was performed with Prism version 7.02 (Graphpad Software Inc.) using the one‐way or two‐way ANOVA test with post hoc Dunnett’s or Sidak’s multiple comparison tests or an unpaired Student’s t‐test, as specified in the figure legends. Significance is represented with *p < 0.05, **p < 0.01, and ***p = 0.01–0.001. Secondary significance calculations are represented with a ^#^p.

### 2.17 Bliss independence calculation

The Bliss independence model can be used to evaluate the drug interaction potential of a drug mixture. The model is based on the principle that each drug has an independent drug mechanism of action, but all contribute to a common result (e.g., cell death or tumor volume inhibition) [29]. The model is based on the probability theory and requires the drug activity of each drug at the selected dose to predict the inhibition rate (IR) at additive doses. For *in vivo* tumor volume results, we predicted the IR based on the fraction of tumor volume remaining after single‐drug treatment of each of the drugs vs 100% CTRL (F_1,2,3,4_), using the following formula: IR = (((F_1_*F_2_) * F_3_) * F_4_).

The IR of DLD1 = (((F_regorafenib_*F_erlotinib_)*F_selumetinib_)*F_vemurafenib_) = (((F_0.90_*F_0.82_)*F_0.62_)* F_1.06_) = 0.489 = 48.9% tumor volume inhibition. The IR of SW620 = (((F_regorafenib_*F_GDC‐0994_)*F_selumetinib_)* F_vemurafenib_) = (((F_0.65_*F_0.55_)*F_0.74_)* F_0.76_) = 0.202 = 20.2% tumor volume inhibition.

## 3 Results

### 3.1 Identification of synergistic multidrug combinations using phenotypically driven therapeutically guided multidrug optimization

In this study, we used the validated TGMO, a phenotypically driven technology for the identification of ODCs specifically targeting CRC cells. A detailed description of the method is provided in Material and Methods and Fig. S1‐2. Briefly, the TGMO‐based screen was performed in a panel of six CRC cell lines of different origin, type, and genetic background, Table S1. The set of eleven drugs included in the TGMO‐based screen consisted of nine TKIs, one IgG‐like protein neutralizing VEGF antibody, and one histone deacetylase inhibitor (HDACi), see Table S2. All drugs were clinically approved or in late‐phase clinical trials, and the selection was based on previously published efficacy and the availability of clinical data on CRC. To start, drug dose–response curves were generated for each drug in each cell line, measuring the efficacy in cell metabolic activity inhibition (ATP levels, presented as % control, Fig. S3). Drug doses corresponding to ED_20_ and half of this dose (ED_10_) were selected to initiate the TGMO‐based screen (Fig. S4). Importantly, selected drug concentrations were at clinically relevant levels, indicated with the plasma concentration limit (PCL) described in Material and Methods and Table S3. In order to identify low‐dose synergistic ODCs, consecutive rounds of screening were conducted (S*earch 1–3*), with the resulting output activity of the drug combinations used to yield step‐wise second‐order linear regression models guiding drug selection towards an optimized drug combination in *Search 3*. Importantly, the search was performed simultaneously in normal, nonmutated colon epithelial (CCD841CoN) cells to identify a therapeutic window (TW), defined as the difference in activity between the nonmalignant cells and CRC cells.

**Fig. 1:**
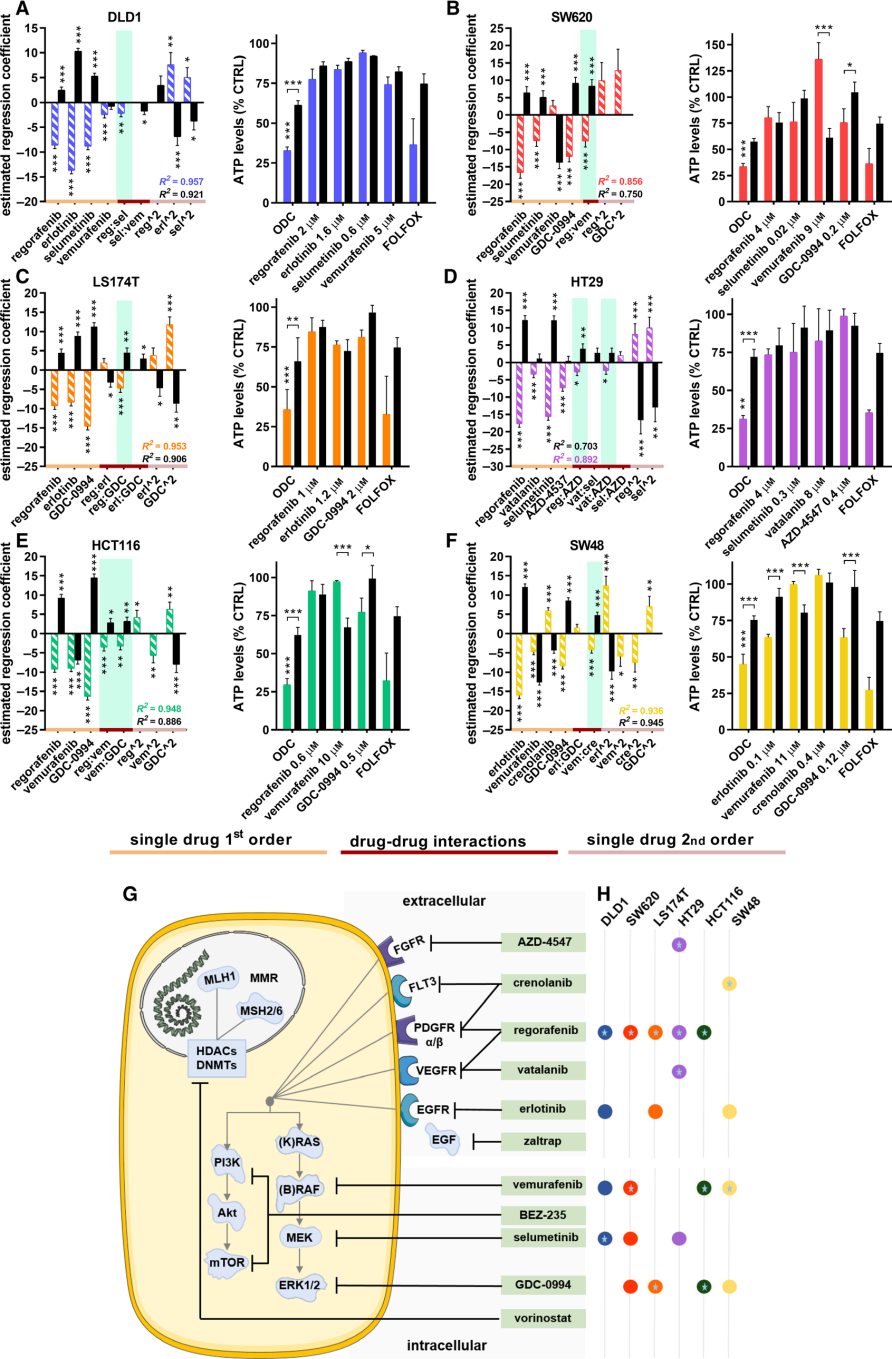
Optimized drug combination (ODC) activity and drug–drug interactions. Estimated regression coefficients of single‐drug first‐ and second‐order activity (orange line and pink line, respectively), and drug–drug interactions (red line) of the a. DLD1, b. SW620, c. LS174T, d. HT29, e. HCT116, and f. SW48 cells (colored/striped bars) and the therapeutic window (solid/black bars) in the left panel. Synergistic drug interactions are highlighted in green. Corresponding right panel presents the activity of the ODCs, corresponding monotherapies and FOLFOX (0.5 µm leucovorin, 10 µm 5‐fluorouracil, 0.5 µm oxaliplatin) as measured by the ATP levels vs. CTRL (<0.15% DMSO) of the CRC cells (colored bars) and the CCD841 healthy colon epithelial cells (black bars) used to obtain the therapeutic window (TW). Error bars represent the SD. *N* = 2‐6 experiments (see legends Figures S4‐S9) and significance of estimated regression coefficients is represented with *P < 0.05, **P < 0.01, and ***P < 0.001, as determined by a one‐way (left graph) or two‐way ANOVA (right graph). g. Schematic of drugs (green boxes) targeting upstream extracellular receptors or downstream intracellular signaling pathways. h. Overview of the optimized CRC cell‐specific drug combinations consisting of 3 or 4 drugs at specific drug concentrations. Colored circles represent the drugs. Drugs part of an interacting drug pair are indicated with a star.

Using the TGMO method, we identified cell‐specific ODCs for the panel of CRC cells. The models generated from each consecutive screening round are presented for all cell lines in Figs S4‐9 (a. *Search 1*, 11‐drug combinations, b. *Search 2*, 7‐drug combinations, c. *Search 3*, 4‐drug combinations, d. efficacy of the drug combinations of all searches combined). The final ODC selection for each cell line consisted of three or four active and synergistic drugs administered at specific doses (Fig. 1A‐F, left graph). Drug interactions are listed in Table S5. The most common drugs in the final selection of ODCs were regorafenib, vemurafenib, and GDC‐0994. In the last optimization step, *Search 4,* doses of the drugs composing the ODCs were optimized and their selectivity clearly outperformed the corresponding monotherapies and first‐line chemotherapy combination FOLFOX used here as positive control (p < 0.001, Fig. 1A‐F, right graph; Table S5).

**Fig. 2:**
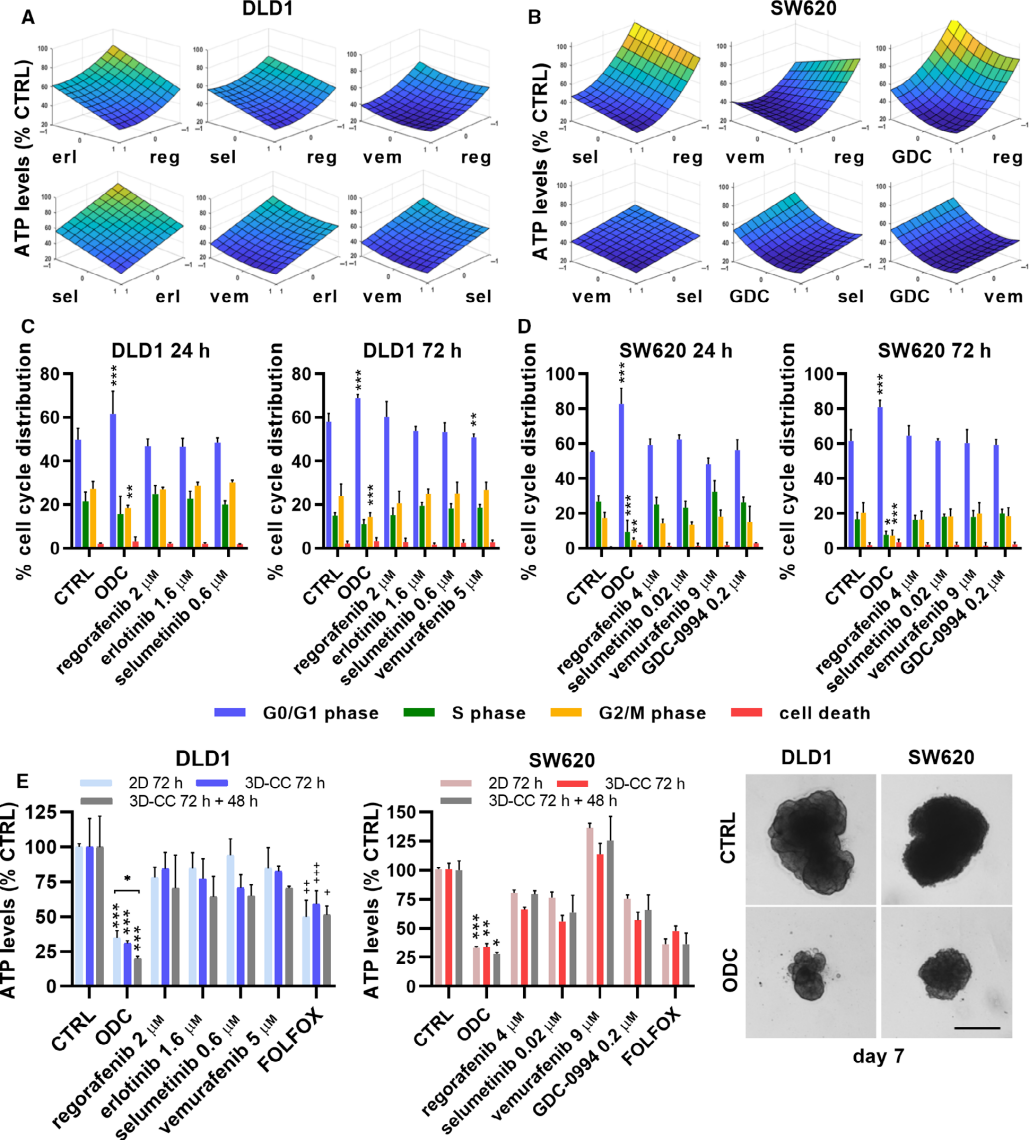
ODC response surfaces, cell cycle distribution, cell morphology, and heterotypic 3D cultures after ODCs treatment. Response surface contour plots in a. DLD1cells and b. in SW620 cells between all two‐drug options after treatment in *Search 3* with the cell‐specific ODCs, fitted with the step‐wise second‐order linear regression model. The y‐axis represents drug efficacy (ATP levels, % CTRL), and the x‐axis represents the dose range (1, high dose, ED_20_; 0, low dose, ED_10_; −1, no drug) for each drug. Abbreviations: reg, regorafenib; erl, erlotinib; sel, selumetinib; vem, vemurafenib; GDC, GDC‐0994. Independent experiments conducted: *N* = 4 (DLD1 24h/72h), *N* = 3 (SW620 24h) and N‐4 (SW620 72h). Cell cycle distribution (G0/G1, S, G2/M phases or cell death) of c. DLD1 and d. SW620 cells after 24h (left graph) or 72h (right graph) post‐treatment with the ODCs, corresponding monotherapies or the CTRL (0.15% DMSO). e. Efficacy in cell metabolic activity (ATP levels, % CTRL) and representative images of heterotypic 3D co‐cultures (3D‐CCs) after (re)treatment for 72h (day 5) or 72h + 48h (day 7) with the cell‐specific ODCs, corresponding monotherapies or the CTRL (0.15% DMSO) of DLD1 and SW620 cells, respectively. 2D cultures were treated on day 1 postseeding. The heterotypic 3D‐CCs consisted of CRC cells in ratio 1 : 1 with healthy colon CCD841CoN fibroblasts and 5% human endothelial ECRF24 cells and were treated for 72 h starting on day 2. Error bars represent the SD of independent experiments conducted for 72h and 72h + 48 h, respectively, with *N* = 3 and 2 for DLD1 and *N* = 2 and 2 for SW620. Significance of *P < 0.05, **P < 0.01, and ***P < 0.001 represent the comparison with the CTRL and monotherapies (c,d,e), while ^+^ *P* < 0.05, ^++^ *P* < 0.01, and ^+++^ *P* < 0.001 represent the comparison between the ODC‐treated and FOLFOX‐treated groups (e) or two‐way ANOVA with post hoc Dunnett’s multiple comparisons test (c,d). Scale bars represent 100 µm and 200 µm, in 2D and 3D, respectively.

A schematic overview of the drugs, drug targets, and cell‐specific ODCs with synergistic drug pairs revealed that the ODCs and synergies associated with upstream and downstream mediators of the MAPK pathway, involved in cell proliferation and survival, see Fig. 1G‐H. Furthermore, we calculated the combination index (CI) using Compusyn, for the ODCs obtained in *Search 3* and *4* (Table S5) and visualized the relationship between the ODC drugs by building response surface contour plots between all two‐drug combinations (Fig. 2A,B and Fig. S10a‐d).

For the DLD1‐specific ODC, the activity was derived from a synergy between regorafenib and selumetinib and additive contributions of other drugs in the combination. The flatness of the dose‐response surface (the 3D representation of dose–dose relations and activity), observed between regorafenib and selumetinib implied that the synergistic activity was maintained with some concentration change (Fig. 2A). Indeed, reducing the drug doses by 25% or 50% resulted in a nonsignificant increase in cell metabolic activity (Fig. S10e). In the SW620‐specific ODC, regorafenib, selumetinib, and GDC‐0994 each contributed to the overall activity and the therapeutic window. Synergy occurred between vemurafenib and regorafenib when administered in combination, thus enhancing ODC activity and enlarging the therapeutic window. The corresponding response surfaces indicated a dose‐dependent relationship between those drugs (Fig. 2B and Fig. S10e). Notably, the ODC activity and TW were enhanced by reducing the dose of regorafenib and selumetinib, while increasing the dose of vemurafenib and GDC‐0994 (Table S4 and S5).

Cross‐validation of cell line‐specific ODCs in other cell lines revealed that the most effective ODCs were those of advanced CRC cell lines DLD1 and SW620, which were also active in all other CRC cell lines (Table S6). Moreover, both ODCs showed strong synergistic potential in the TGMO models as well as the CI. Further results for DLD1 and SW620 cells are presented in the main figures, whereas data on other CRC cells are listed in the Supplementary Figures.

### 3.2 ODCs induce changes in cell cycle and cell morphology and are active in heterotypic 3D co‐cultures

To investigate the underlying mechanisms of cell metabolic activity inhibition in the CRC cells after ODC treatment, we measured the cell cycle distribution by flow cytometry in cells exposed to ODCs for 24h and 72h. We observed a significant G0/G1 phase arrest in ODC‐treated DLD1 ODC cells compared to the control (CTRL) cells (61.6% vs. 49.7%, p < 0.001), which was associated with a reduced number of cells in S‐ and G2/M‐phase (Fig. 2C, 24h, left graph). For the cells exposed to the ODCs for 72h, the percentage of cells arrested in G0/G1 increased to 68.9% (p < 0.001, Fig. 2C, right graph). The ODC‐treated SW620 cells had a significantly higher percentage of cells arrested in the G0/G1 phase after 24h compared to the CTRL cells (82.9% vs. 55.2%, p < 0.001), which remained constant after 72h (Fig. 2D). The results for other cell lines are presented in Fig. S11a‐d. These results indicate that the cell metabolic activity inhibition observed after ODC treatment is the result of cell cycle inhibition or induction of cell death.

To visualize treatment‐related changes in cell morphology, CRC cells were stained for F‐actin (phalloidin) and the nucleus (DAPI). We observed pronounced cell clustering among all cell lines (Fig. S12a, representative images). Structural changes in the actin skeleton (i.e., stress fibers) were observed for DLD1, but this did not result in significant changes in the cell body or nucleus size (Fig. S12b). For the remaining cell lines, the most notable effect was a decrease in cell size in HCT116 cells after ODC treatment, see Fig. S12c‐f. This effect was induced by vemurafenib and GDC‐0994. Of note, vemurafenib as a single drug significantly enhanced the capacity of HCT116 cells to form tunneling nanotubules (TNTs,> 10 µm) protruding from the cell (14.0% vs. 9.5%, p < 0.01, Fig. S12g and representative image of TNTs in CTRL in Fig. S12a). In addition, these TNTs also tended to be longer compared to the TNTs observed in the CTRL cells (32 vs. 21 µm, nonsignificant, Fig. S12h). TNTs are known to facilitate intercellular communication with the exchange of intracellular materials such as signaling molecules, vesicles, and even whole organelles [30]. TNT formation was stimulated in vemurafenib‐treated cells, significantly reduced in regorafenib or GDC‐0994‐treated cells (0.95 and 0.87 µm, p < 0.001), and completely inhibited through the administration of the HCT116‐specific ODC (Fig. S12g).

The efficacy of the ODCs above was identified in the 2D *in vitro* screening. As a next step for translating cell‐specific ODCs toward *in vivo* application, we evaluated the effect of the ODCs in heterotypic 3D co‐cultures (3D‐CCs), which model more faithfully tumor organization and microenvironment. The 3D‐CCs were composed of CRC cells in ratio 1:1 with normal human colon CCD841CoN fibroblasts and 5% human ECRF24 endothelial cells (500:500:50 cells). We found that the ODCs were similarly or frequently even more potent in the 3D‐CCs (p < 0.01‐0.001) and the effect was more pronounced after retreatment (day 5‐7 postspheroid formation, adding an additional volume of 1x concentrated drugs), see Fig. 2E and Fig. S13a‐d.

### 3.3 ODCs effectively inhibits tumor growth in subcutaneous models

**Fig. 3:**
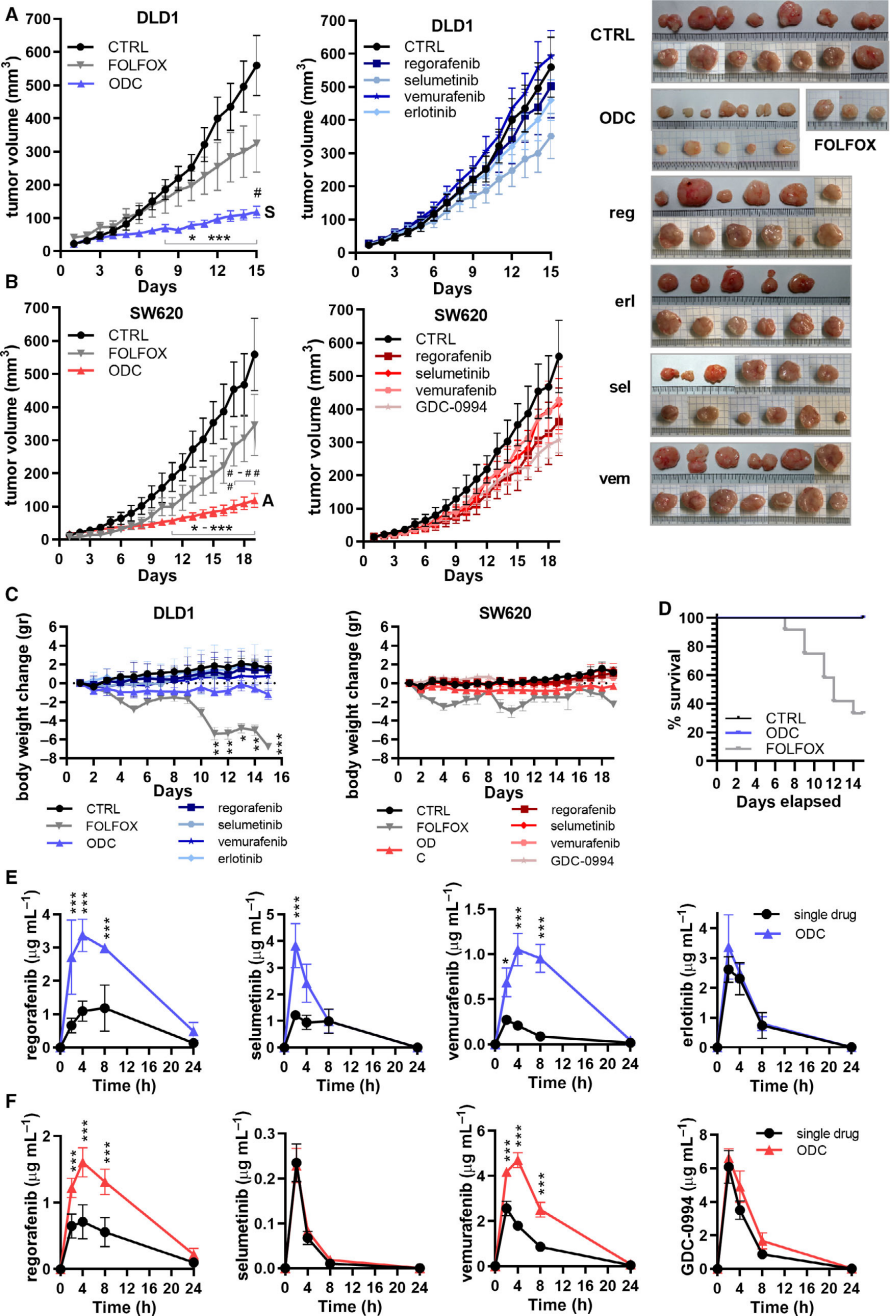
DLD1‐specific ODC efficacy *in vivo* and pharmacokinetics of the drugs composing. The ODC. a. DLD1 and b. SW620 tumor growth curves and representative images of subcutaneously implanted tumors in Swiss Nu/nu mice after 15 or 19 days of daily treatment, respectively, in *N* = 2 independent experiments, respectively, with CTRL, ODC, and FOLFOX (left graph) and corresponding monotherapies (right graph). Synergy (S) and additivity (A) of the overall combination is indicated for DLD1 and SW620, respectively. For DLD1, significance was observed for ODC (*n* = 16) compared to CTRL (sham, *n* = 13), FOLFOX (*n* = 4), 15 mg/kg regorafenib (*n* = 12), 12.5 mg/kg erlotinib (*n* = 11), 5 mg/kg selumetinib (*n* = 11), and 75 mg/kg vemurafenib (*n* = 13). For SW620, significance was observed for ODC (*n* = 16) compared to CTRL (sham, *n* = 7), FOLFOX (*n* = 4), 30 mg/kg regorafenib (*n* = 8), 0.2 mg/kg selumetinib (*n* = 6), 75 mg/kg vemurafenib (*n* = 7), and 10 mg/kg GDC‐0994 (*n* = 11). FOLFOX was administered as 6 mg/kg oxaliplatin two hours before administration of 90 mg/kg leucovorin and 50 (DLD1) or 25 (SW620) mg/kg 5‐fluorouracil at day 1 and a repeat of 5‐fluorouracil dosing on day 2. c. Weight loss of ODC‐, CTRL‐, and FOLFOX‐treated mice over time of mice with DLD1 and SW620 tumors, respectively. d. Survival of ODC‐, CTRL‐, and FOLFOX‐treated mice with DLD1 subcutaneous tumors over time. The survival of FOLFOX‐treated mice is reduced to *n* = 4 at the experimental endpoint. e,f. Drug concentrations in blood serum at 2h, 4h, 8h, and 24h post‐treatment with the ODC or the corresponding monotherapies of mice carrying subcutaneous DLD1 or SW620 tumors (*N* = 4). Error bars represent the SEM (a,b) or SD (c,e,f) and significance of *p < 0.05, **p < 0.01, and ***p < 0.001 represent the comparison with the ODC (a,b), no weight loss (g,h) or the comparison between the drug administered as single drug or as part of the ODC (e,f) using a two‐way ANOVA with post hoc Dunnett’s (a,b,c) or Sidak’s (e,f) multiple comparisons test

To evaluate the anti‐tumor activity of the ODCs *in vivo* models, DLD1 and SW620 cells were inoculated subcutaneously and tumors were allowed to develop in male and female Swiss nu/nu mice. When solid tumors were formed, mice were randomized. In the first step, several doses of single drugs were tested in order to select for suboptimal low doses *in vivo* corresponding to the effect of individual drugs *in vitro* (Tables S7‐9 and Fig. S14). The activity of the ODCs was compared with current clinical first‐line CRC treatment, that is, FOLFOX. Strikingly, the ODCs significantly outperformed the FOLFOX treatment and the corresponding monotherapies, resulting in approx. 80% of tumor growth inhibition (Fig. 3A,B, see images of the resected tumors on the right). The DLD1‐specific ODC was highly effective and acted synergistically, as observed in the *in vitro* screen (Fig. 3A). The SW620‐specific ODC inhibited tumor growth effectively in an additive manner, due to higher activity of vemurafenib as compared to *in vitro* conditions (Fig. 3B and Methods Bliss independence calculation). The images of the resected SW620 tumors can be found in Fig. S15a.

Chemotherapy in CRC is associated with significant side effects, including gastrointestinal toxicity [31] and cachexia [32]. Importantly, while ODC‐treated mice did not gain weight over time as compared to the control and single‐drug treatment groups, no statistically significant weight loss was observed in the ODC‐treated group (Fig. 3C). Resected tumor weights are compared in Fig. S15b. Conversely, the administration of FOLFOX resulted in significant weight loss (approx. 20%, p < 0.01–0.001) and reduced survival (Fig. 3C‐D). Additional adverse effects, such as low‐grade diarrhea, were only observed in the FOLFOX‐treated mice. Heart volume and heart weight were used as indicators for toxicity and cachexia. The % heart volume over total mouse body weight in mice treated with the DLD1‐ and SW620‐specific ODCs remained unchanged (Fig. S15c).

### 3.4 Synergistic ODCs increase drug concentrations in blood and in tumor tissues

To investigate drug pharmacokinetics of the ODC compared to the single‐drug treatments, we obtained drug concentration‐time profiles from whole blood of treated animals analyzed using LC‐MS/MS system. To visualize both the C_max_ (maximum observed concentration in 0–24 h in µg*h/mL) and drug elimination overtime for all of the drugs constituting the ODCs, 5 µL of blood samples were collected from the tail vein at 2h, 4h, 8h, and 24h post‐treatment. Drug concentrations measured were used to calculate the AUC_0–24h_ (area under the curve over 0–24 h in µg*h/mL) and the C_max_ (maximum observed concentrated in 0–24h in µg*h/mL), as summarized in Tables S10‐S11, for DLD1 and SW620 models, respectively.

We observed a significant increase of drug availability over time in AUCs for regorafenib, selumetinib, and vemurafenib (p < 0.0003, 2.8x, 1.6x and 6.6x higher, respectively), but not for erlotinib, when administered in mice with DLD1 tumors as part of the ODC as compared to the single‐drug treatments (1.14x higher, Fig. 3E). A similar trend was observed in mice bearing SW620 tumors with a significant increase noted for regorafenib and vemurafenib (p < 0.0001, 2.2x and 2.5x, respectively). This effect was only marginal for GDC‐0994 (1.4x increase) and not observed for selumetinib (1.2x increase) administered at a 150–200‐fold lower dose compared to the converted clinically relevant dose in mice (0.2 mg/kg vs. 31–41 mg/kg, Fig. 3F). Finally, whereas relatively high AUC_0–24h_ values were noted for regorafenib, erlotinib, and selumetinib administered at low doses, administration of vemurafenib (75 mg/kg) induced low AUC_0–24h_ values.

Furthermore, to compare drug concentrations in blood plasma and inside the tumor at the experimental endpoint in DLD1 tumors (day 15) and SW620 tumors (day 19), we treated them 1h pre‐euthanization, extracted blood and tumor tissue samples, and analyzed drug concentrations by LC‐MS/MS system. Interestingly, in DLD1‐bearing mice ODC‐treated tumors displayed significant accumulation of regorafenib and erlotinib (p < 0.001 and p < 0.01, respectively), differently from single‐drug treatments (Fig. S15d and Table S12). This effect was at least in part independent of the increased bioavailability observed with the AUC_0–24h_ as evidenced by the accumulation of the drugs. Conversely, in SW620‐bearing mice, the opposite trend was observed (p < 0.0001 for GDC‐0994). As a correlation between tumor weight and drug concentration could be excluded (Fig. S15e‐f), it indicates a strong drug efflux mechanism in the tumor cells.

### 3.5 ODC reduces tumor cell proliferation, microvessel density and the number of reticular fibroblasts

The ODC activity on DLD1 and SW620 tumors was further confirmed by IHC analysis of tumor heterogeneity (H&E), tumor endothelium (CD31), or by fluorescence staining for proliferating tumor cells (Ki67). Representative images and image‐based quantification are shown for DLD1 (Fig. S16a) and SW620 (Fig. S16b) tumors. H&E and Ki67 staining revealed a significant decrease in areas with tumor cells (H&E) in the ODC‐treated group, and proliferating nuclei in the ODC and vemurafenib‐treated groups, as compared to the control and monotherapies. Furthermore, ODC treatment significantly reduced microvessel density (MVD).

### 3.6 ODC synergistically inhibits tumor growth in CRC orthotopic model

**Fig. 4:**
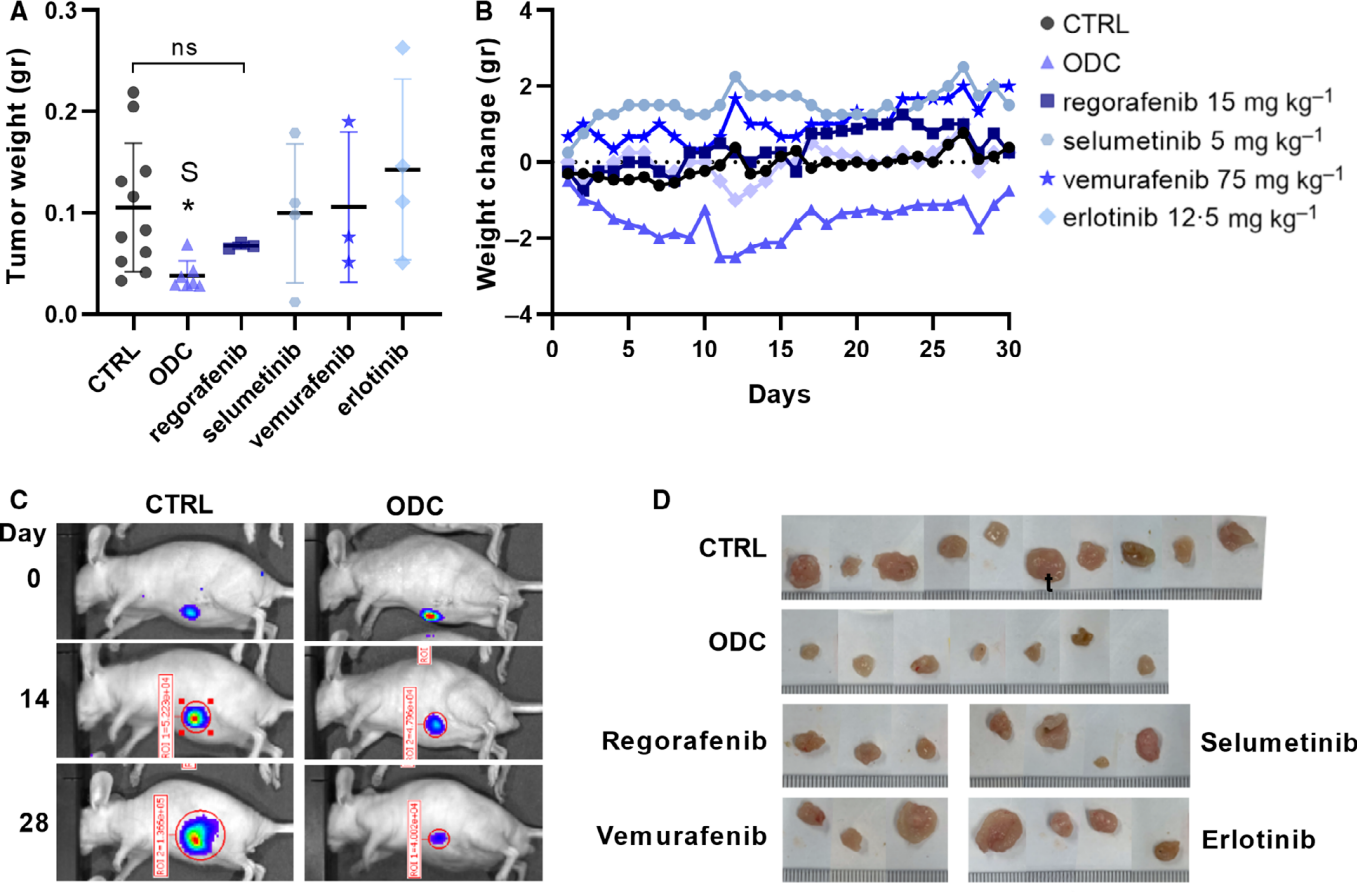
Optimized drug combination efficacy in DLD1 orthotopic *in vivo* model. a. Tumor weight of DLD1 orthotopic xenografts at the experimental endpoint after 30 days of daily treatment with CTRL (*n* = 11), ODC (n‐7), 15 mg/kg regorafenib (*n* = 3), 12.5 mg/kg erlotinib (*n* = 4), 5 mg/kg selumetinib (n = 4), and 75 mg/kg vemurafenib (n = 3). With an average of 64% inhibition on tumor weight for ODC treatment vs. 100% CTRL, synergy (S) was confirmed based on Bliss independence (64% observed inhibition vs 17% predicted additive activity on tumor weight). b. Mice weight change over time. Standard deviation (SD) increased over time with an average SD in grams of 1.24 for control, 1.24 for ODC, 1.56 for regorafenib, 1.23 for selumetinib, 0.83 for vemurafenib, and 1.84 for erlotinib. c. Representative bioluminescence images of DLD1 tumors at days 0, 14, and 28 of treatment and d. representative images of the DLD1 tumors after resection at the endpoint. Significance of *p < 0.05, **p < 0.01, and ***p < 0.001 represent the comparison of the ODC with all other groups using an unpaired Student’s t‐test (a) or the comparison with no weight loss using a two‐way ANOVA with post hoc Dunnett’s multiple comparisons test (b)

To investigate, if the anti‐tumor activity of the DLD1 ODC will remain active in an orthotopic CRC model, DLD1 luciferase‐expressing cells were inoculated intracecally in male and female Swiss nu/nu mice. Two weeks after implantation luciferase activity was measured by bioluminescence and used as an indicator of tumor take (91.5%) and size for mice randomization into treatment groups. After daily treatment, the ODC mice were euthanized and tumors were weighed. The ODC significantly and synergistically (S) inhibited tumor weight compared to CTRL (p = 0.0150) and significantly outperformed corresponding monotherapies (p < 0.05, Fig. 4A), while causing no significant weight loss (Fig. 4B). Bioluminescence measurements confirmed CTRL tumor expansion with time, while ODC tumor growth was arrested or inhibited (Fig. 4C). Finally, the macroscopic inspection of the ODC tumors confirmed the results obtained on tumor weight measurements (Fig. 4D).

### 3.7 RNA sequencing reveals differentially expressed genes after ODC treatment

**Fig. 5:**
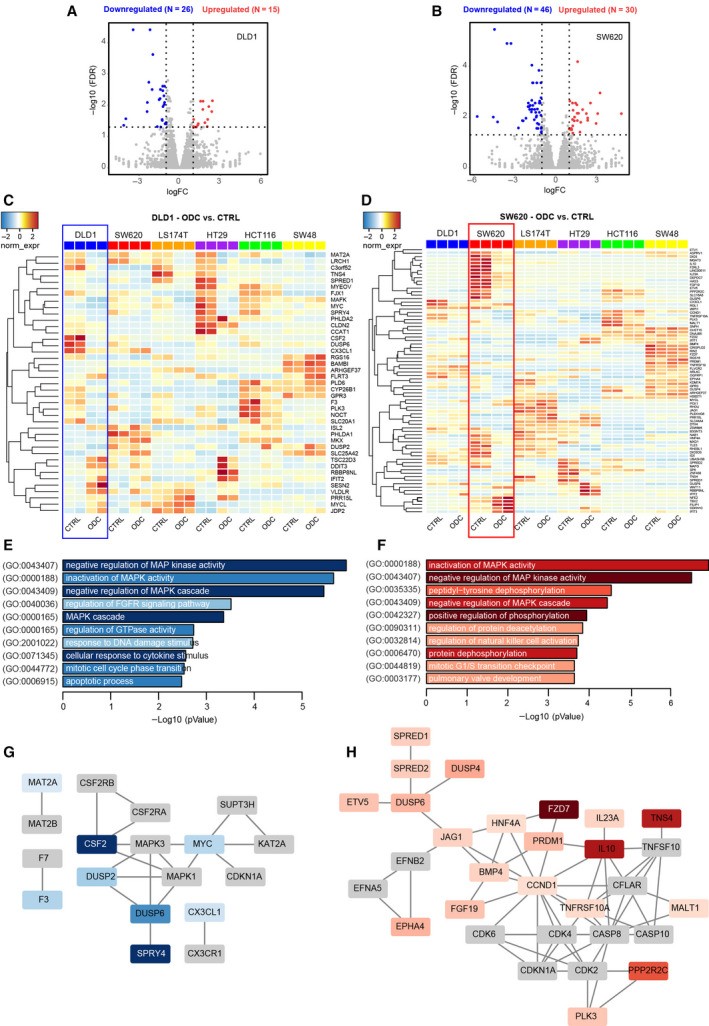
Cell‐specific ODCs decrease gene expression in the MAPK network. Differential gene expression analysis was performed based on RNA‐sequencing (RNA‐seq) analysis of ODC‐treated CRC cells relative to vehicle control (CTRL) of N = 2 (n = 4) independent experiments. Volcano plots of significant genes (p‐value < 0.05) and a fold change> 2 (logFC) in a. DLD1 and b. SW620 cells. Heatmap of genes in c. DLD1 and d. SW620 cells differentially up‐ and downregulated after 2h of ODC treatment compared to CTRL (0.15% DMSO). Color coding is based on the fold change (red = significant upregulated genes, blue = significant downregulated genes). Vertical lines highlight log2 fold changes of −1 and + 1, while a horizontal line represents a corrected for multiple test p‐value of 0.05. e,f. Enrichment analysis of downregulated genes in DLD1 (n = 26) and SW620 (n = 46) for Gene Ontology—Biological Process. The top 10 functional clusters are sorted according to p‐value, and color intensity is proportional to the number of represented genes per ontology. g,h. Protein interaction analysis of downregulated genes. Using STRING (string‐db.org), an enhanced network up to 10 interactors per 1^st^ order shell in DLD1 cells (g) and SW620 cells (h) was performed. Only connected nodes are shown. Nodes in gray are first‐order interactors of the differentially expressed genes, which are color‐coded proportionally to fold change

To identify the early effects of the ODCs on the RNA transcriptome, we performed RNA sequencing on the CTRL and ODC cells treated for 2 hours. We observed different gene expression signatures for each of the cell lines, confirming genetic heterogeneity within the panel of CRC cell lines included in this study. Consistently, more genes were downregulated than upregulated by the ODC treatment. For example, in DLD1 cells, expression of 41 genes was significantly altered, of which 26 genes were downregulated (Fig. 5A,C). Several downregulated genes were targeted by kinases in the MAPK, RAS, and ERK signaling pathway. Other downregulated genes were TNS4 (a regulator of MET); MYC and CCAT1 (an oncogene and regulator of MYC), PLK3 (a regulator of the cell cycle), various GPCR regulators. In SW620 cells, 76 genes were differentially expressed (Fig. 5B,D), of which 46 were downregulated, most notably a 53‐fold decrease for frizzled class receptor 7 (FZD7), a receptor of Wnt. Differential gene expression analysis for other cell lines is presented in Figs S17‐S18 and the top 50 downregulated genes are listed in Table S13.

Differentially expressed genes were analyzed in Enrichr (www.enrichr.org) for gene ontology biological process enrichment (GO‐BP) (Fig. 5E‐F, Figs S17‐S18). These data showed an extensive impact on MAP kinase activity in all cell lines, which is consistent with the panel of drugs presented for the different ODCs, whose direct and downstream targets include MAP kinases. We also analyzed protein–protein interactions of differentially expressed genes including first‐order interactions using STRING analysis, first‐order. This revealed ODC‐mediated downregulation of MAP kinases and cyclin/CDK complexes, while inflammatory signatures were also prevalent (Fig. 5G,H, Figs S17‐S18).

### 3.8 Phosphoproteome profiling suggests the molecular signature of active drug targets in CRC cell lines

**Fig. 6:**
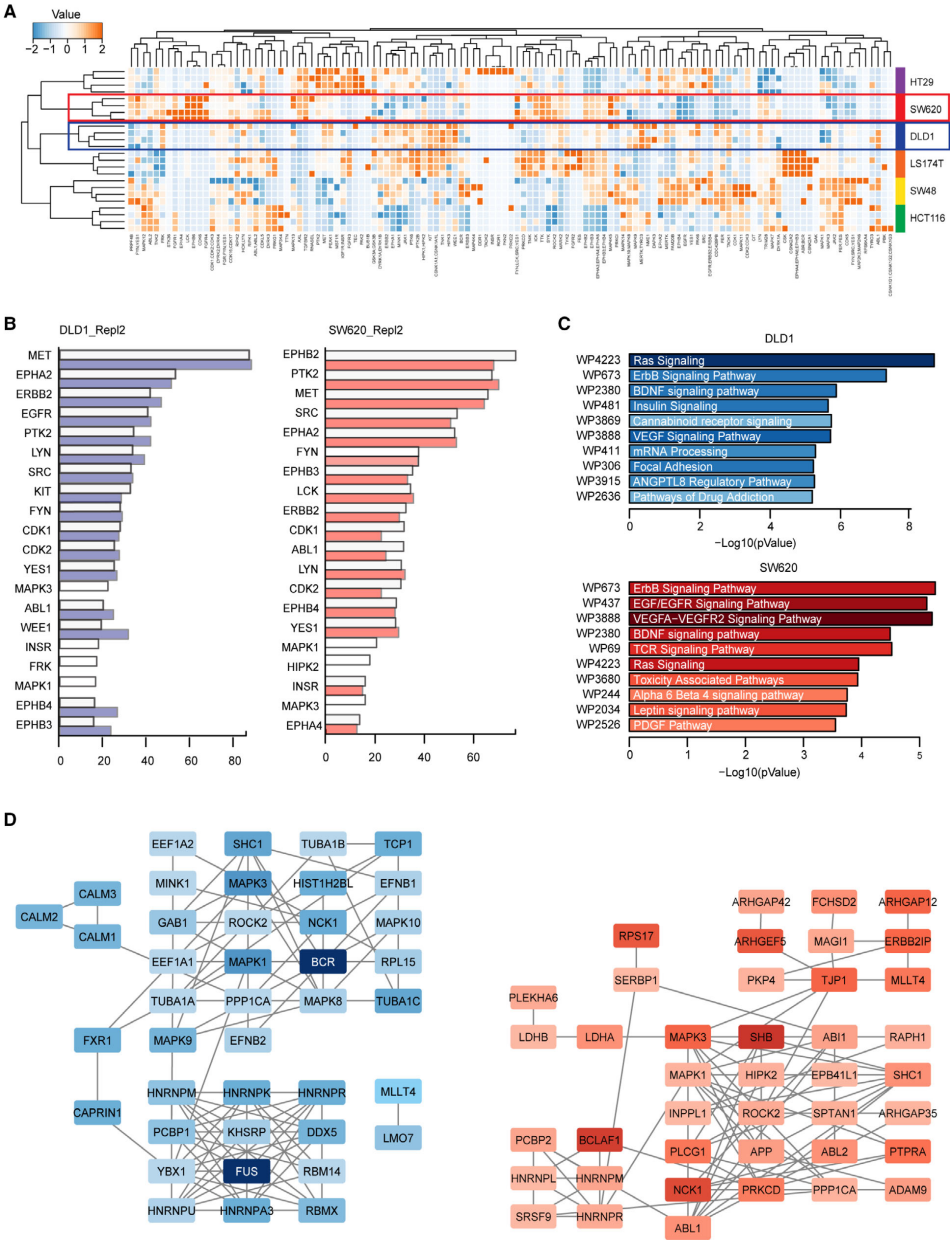
Phosphoproteomic profiling of CRC cell lines treated with ODCs. a. Heatmap and unsupervised clustering of phosphorylated kinases in six CRC cell lines (DLD1 in the blue frame, SW620 in the red frame) of N = 2 independent experiments. Color coding is based on normalized spectral counts measured, with relative expression scores per kinase depicted. b. Top 20 INKA scores in vehicle‐treated DLD1 (left) and SW620 (right) (white bars) with profiles after the indicated treatments superimposed in colored bars. c. Pathway enrichment analysis (WikiPathways) of downregulated phosphoproteins in DLD1 (N = 49) and SW620 (N = 59). The selection of phosphogenes was based on the sum of normalized spectral counts> 5 and fold change> 1.5 in both replicates. The top 10 functional clusters are sorted according to p‐value, and color intensity is proportional to the number of represented genes per pathway. d. Protein interaction analysis of downregulated phosphoproteins using STRING (string‐db.org). Nodes are color‐coded proportional to fold change. Left: DLD1, right: SW620 cells

In the next step, we directly investigated the signaling pathways involved by mass spectrometry analysis of phosphopeptides, enriched in CRC cells treated with the ODC. Following sequence database searches, the phosphopeptide data were aggregated to represent individual phosphorylated proteins, and phosphorylated kinases (phosphokinases) were subsequently identified. Unsupervised cluster analysis of phosphokinase expression (based on spectral counts) was performed to explore drug effects (Fig. 6). The heatmap showed major intrinsic differences in phosphokinase expression of the different cell lines, as cell line clustering played a major role in the treatment effect (Fig. 6A). Phosphokinase abundance showed only minor differences between ODC and CTRL for essentially all cell lines. We thus decided to analyze our data for kinase activity rather than kinase phosphorylation levels using the Integrative Inferred Kinase Activity (INKA) method. The INKA analysis of the top 20 ranked most active kinases showed considerable overlap among the cell lines (Fig. 6B, Fig. S19), suggesting common mechanisms driving oncogenesis in the CRC cells despite their differential mutation status. ODC treatment had only limited measurable effect on kinase activity directly as evidenced by the high degree of overlap between INKA scores in CTRL (outlined white bars) and after ODC treatment (overlaid colored bars), Figure 6B, Figure S19. However, interestingly, MAPK1 and MAPK3 activity were abrogated after treatment in DLD1, SW620 (Fig. 6B, bars in white), and in HT29 cells (Fig. S19). HCT116 cells displayed a consistent suppression of WEE1 kinase activity upon ODC treatment, as well as inhibition of EPHA2 and ERBB2 activity. SW48 cells showed strong inhibition of EGFR activity upon ODC treatment while in LS174T cells global profiles changed only little (Fig. S19). From the network analysis of the top 20 active kinases and their substrates, EGFR, SRC, ERBB2, ABL1, and MET are the most interconnected hub kinases in all cell lines. In concordance, after ODC treatment the major hubs retained a high level of connectivity (Fig. S19). Taken together, these data indicate that on single kinase activity level, the ODCs of the different cell lines had only subtle effects while major inhibition was seen downstream on MAPK1/3.

Pathway enrichment analysis for differentially expressed phosphorylated proteins, rather than only kinases, prior and post‐treatment, identified suppression of growth factor‐driven pathways, including EGFR, VEGFR, ErdB, and Ras as a major effect of ODC treatment (Fig. 6C, Fig. S20). Protein–protein interaction analysis of these differentially expressed phosphoproteins showed densely interacting clusters of proteins (Fig. 6D, Fig. S20), except for LS174T and HT29 cells, which showed a relatively low number of consistently suppressed phosphoproteins (N = 24 and N = 15, respectively). A global comparison of the remaining cell networks revealed several remarkable similarities. Clusters of ribonucleoproteins, involved in RNA processing, are present in different configurations. Clusters containing MAPKs are also evident. In several cases, a clear driver protein can be identified, such as PI3K in HCT116 and EGFR in SW48 (Fig. 6D, Figure S20). We also presented in silico analysis of target proteins in CRC cells (Figure S21). Together, these data demonstrate that the cell‐specific ODCs affect different signaling pathway components, yet similar cellular processes (i.e., cell proliferation and survival by inactivation of oncogenic MAPK signaling).

### 3.9 ODCs are active in cells from patient’s CRC liver metastases

In order to validate the ODCs activity on metastases of human CRC tumors, we cultured single‐cell suspensions isolated from freshly resected CRC liver metastases and patient‐matched samples of normal liver tissue (Figure S22a). Due to intrapatient variability, not all specimens obtained from both the metastatic and liver cell suspensions led to proliferating cultures and the outgrowth of spheroids/cells (Figure S22b‐c). Nevertheless, the cultures of six patient‐derived samples revealed enough viable cells to investigate responses to ODCs and corresponding monotherapies. The incubation of those cells with the ODCs for 72 hours resulted in a reduction of the cell viability by ~50% (Figure S22d). Significantly, in tumor cell cultures of patients treated with chemotherapy including 5‐fluorouracil, all ODCs tested clearly outperformed 5‐fluorouracil confirming the loss of sensitivity to the chemotherapy only.

## 4 Discussion

In this study, we used an improved variant of our recently developed s‐FSC technology [21, 22, 33], called the therapeutically guided multidrug optimization method (TGMO), to identify effective and selective multidrug combinations specific for CRC cells. This technology allows the selection of a high‐order (>2 drugs) low‐dose optimized drug combination (ODC) from a large search space, by experimental testing of only a small fraction of all possible drug combinations and predictive data modeling [18]. Applied to a set of human CRC cell lines, high‐order low‐dose ODCs were identified. We now showed that high therapeutic selectivity, that is, maximized difference in effects on CRC and nonmalignant cells, can be achieved by multitarget inhibition, which is in line with previous findings [17, 34, 35] and superior to FOLFOX‐treated selectivity and drug–drug interactions [36].

We observed that the selectivity for our ODCs was dependent on differences in dose sensitivity in CRC cells and normal cells, synergies as well as antagonisms between the drugs in tumor and normal cells. We commonly found both antagonisms and synergisms in the CRC cells in *Search 1* of the TGMO‐based optimization. Through rounds of eliminating of antagonistic drugs and selection of the most active drugs, we observed in *Search 3* drug synergisms in CRC cells and drug antagonisms in the normal cells. These observations are in line with the findings of Weinstein *et al*. who tested two‐drug dose‐escalating checkerboards and described that drug interactions may enhance, diminish or invert the therapeutic window [37]. Furthermore, it was shown that targeting of key signaling pathways accommodated the occurrence of synergies between drugs in a wide variety of genetically varying CRC cell lines, and that high‐order drug combinations were a requirement to kill cancer cells effectively [15].

As most of the drugs in this study target sequential steps in the dominant MAPK pathway, a certain level of redundancy may be expected. However, as each drug only causes partial inhibition of cell metabolic activity, each target might also undergo incomplete inhibition. Nevertheless, in general, the transcriptomics and phosphoproteomics analyses of ODC effect confirmed the shutdown of MAPK3/1 (i.e., ERK1/2) signaling. This could be dependent on the cell‐specific differences in drug combinations which are in turn derived from the cell‐specific differences in oncogenic activation of signaling pathways for each of the cell lines. Similar observations were found by Neto *et al*. in *EGFR^mut^* non‐small‐cell lung cancer cells; low‐dose inhibition of RAF/MEK/ERK with or without EGFR inhibition could completely block *MAPK* signaling without toxicity [17]. Similarly, Caumanns *et al*. reported that low‐dose triple‐drug combinations targeting the PI3K/AKT/mTOR and MAPK signaling pathways resulted in cumulative kinase activity inhibition and diminished tumor growth without inducing toxicities in ovarian carcinoma cells [38]. Importantly, this indicates that simultaneous multidrug modulation of key ontogenically activated pathways can successfully inhibit tumor growth in various cancer types.

We further observed that elimination of receptor inhibition, but not intracellularly acting inhibitors from the ODC, significantly diminished its efficacy suggesting the importance of upstream signaling inhibition. However, as the cells may evoke alternative signaling pathways in response to upstream inhibition, targeting of pathways through both upstream and downstream is needed [39]. In our study, all cell type‐specific ODCs include drugs both upstream and downstream of *RAS* and *RAF*. Specifically, we identified three synergistic drug pairs composed of upstream regorafenib (mediated receptor inhibition of mainly PDGFRα/β and VEGFR), with selumetinib or GDC‐0994 (inhibiting MEK or ERK1/2, respectively). Potentially, the synergy we found could depend on the feedback loops inhibition of one drug caused by the activity of another drug. An autocrine feedback loop via MEK and ERK has previously been reported upon EGFR inhibition [40]. Moreover, combining gefitinib (EGFR inhibitor) and PD98059 (MEK inhibitor) resulted in synergistic induction of cell death in breast cancer cells [41]. Similarly, inhibition of VEGFR2 in lung cancer cells induced a feedback loop through the MAPK signaling pathway, which could be interrupted by adding a MEK inhibitor [42]. The inhibition of feedback mechanisms is one of the main mechanisms of synergy between drug pairs according to Jia *et al*. [39].

Furthermore, although we did not perform molecular profiling after therapy withdrawal, it was shown that retreatment remained effective, suggesting that multilevel targeting does not (immediately) lead to the selection of resistant cell populations. Indeed, Caumanns *et al*. reported that the simultaneous administration of the triple‐drug combination prevented the induction of feedback mechanisms [38]. Importantly, Neto *et al*. noted that multinode inhibition could overcome resistance to high dose single‐drug inhibition.

The current consensus on molecular subtypes of CRC classifies tumors into four subgroups (CMS subgroups; see recapitulated in Table S1) [8]. This classification includes mutation status, chromosomal and microsatellite (in)stability, and methylation phenotype. Importantly, the TGMO‐based screen identified potent ODC for all cell lines and patient material even those from CMS4, known to have the worst prognosis.

Most CRC patients (60–80%) have constitutively active or overexpression of EGFR and therefore should respond to anti‐EGFR treatment. However, 45% of patients have additional mutations in downstream *KRAS, NRAS,* and/or *BRAF* and therefore do not respond well to anti‐EGFR treatment [43]. Indeed, EGFR‐overexpressing SW48 cells were the most sensitive to the EGFR‐targeting drug (erlotinib). BRAF^V600Emut^‐expressing HT29 cells had a high sensitivity to BRAF inhibitor (vemurafenib). Interestingly, low‐dose vemurafenib treatment in SW620 cells resulted in increased cell viability. This phenomenon, known as the hormetic dose effect, has been previously reported in *BRAF* wild‐type tumors where vemurafenib paradoxically stimulated RAF dimerization and RAS interaction independently of RAF kinase inhibition, thereby inducing MEK activation [44, 45]. In our studies, out of the five BRAF wild‐type cell lines, SW620 cells were the only ones to respond in this manner. SW620 cells are unique compared to other cell lines as they carry the *KRAS^G12V^* mutation associated with differential downstream pathway regulation and a poor prognosis [46]. In general, such a hormetic dose effect is observed more commonly and mostly arises from a compensatory feedback response [47]. Importantly, when vemurafenib was administered as part of ODCs, this stimulatory effect is negated by effective multinode targeting of the pathways involved.

Pharmacokinetics analysis showed that drug concentrations of regorafenib and vemurafenib in whole blood were significantly higher when administered in combination with other drugs as compared to individual drug administration. Interestingly, in the DLD1, but not in the SW620 tumor model, intratumor drug concentrations were significantly higher when the drug was part of the ODC, as compared to monotherapy. These higher intratumor drug concentrations in DLD1 vs. SW620 were consistent with the observed synergistic effect in DLD1 vs. the additive effect in SW620 *in vivo*. In the SW620 tumor model, we observed a decrease in intratumor drug concentrations in SW620 tumors vs. blood plasma, which indicates the possible acquisition of an efflux mechanism. Indeed, previous studies have reported drug efflux mechanisms in SW620 cells, including drug efflux pump MRP2 and LRP [48, 49].

In mice, regorafenib is metabolized by Cyp3a11 and Ugt1a9 and transported to the liver by Oatp1b2 [50]. Moreover, the observed increase in the AUC_0–24h_ might be due to the inhibition of Cyp3a11, Ugt1a9 or Oatp1b2 by one or more drugs composing ODC, as regorafenib is unlikely to be a substrate of efflux transporters mdr and bcrp [51, 52]. In humans, vemurafenib is metabolized by CYP3A [53], while selumetinib is by CYP2C19 and UGT1A1 [54]. Both vemurafenib and selumetinib are substrates of the drug efflux transporters P‐glycoprotein and breast cancer resistance protein [53, 55]. Inhibition of CYP3A, CYP2C19, or efflux transporters may explain the increase in the AUC_0–24h_ ratios between single drugs and ODC for the two drugs. Tumors also express a high level of metabolic enzymes and efflux transporters. Inhibition of metabolic enzymes and/or of efflux transporters may explain the increase in drugs AUC_0–24h_ ratios between single drugs and ODC.

Pharmacokinetics of GDC‐0994 and erlotinib were not affected by the presence of other drugs in the cocktail. No data are available for GDC‐0994 regarding its metabolism and transport. Erlotinib is known to be metabolized by CYP3A and is a substrate and an inhibitor of P‐glycoprotein, as well as breast cancer resistance protein. It could be therefore responsible for increased blood and tumors concentrations of regorafenib, selumetinib, and vemurafenib in DLD1 tumors [56]. More *in vitro* and *in vivo* studies would be needed to be performed in order to characterize drug–drug interactions between the molecules in the ODC cocktails.

To gain more insight at the underlying actions of the ODC, changes in phosphorylated kinases, as well as RNA expression, were addressed. The dominant mechanism of action of the ODCs observed at phosphoproteomic and RNA levels was on inhibition of MAPK signaling. At the cellular level, the blockade of this pathway is linked to apoptosis and cell cycle arrest [57]. Notably, we observed abundant representation of MAPK Pathway Activity Score (MPAS) signature genes, the so‐called transcriptional (*SPRY2, SPRY4, ETV4, ETV5, DUSP4, DUSP6, CCND1, EPHA2, and EPHA4*), among the differentially downregulated genes after ODC treatment. MPAS represents a relative score of the expression of these genes and was reported to be a clinically relevant biomarker in multiple cancer types. High MPAS is associated with poor prognosis in primary and metastatic CRC, and outperforms genome‐ and mutation‐based prediction models for sensitivity to MEK inhibition [58]. Indeed, in each cell line, a selection of MPAS genes was downregulated, independently of *BRAF, KRAS,* or *TP53* mutation status. Differentially expressed genes connect to cyclin‐CDK (cyclin‐dependent protein kinase) complexes, which regulate cell cycle progression, explaining our observations on cell cycle arrest. Phosphoproteomics analysis revealed inhibition of MAP kinase activity after ODC treatment, especially MAPK3/1 (i.e., ERK1/2).

In our study, there are numerous unchanged transcripts found in the RNA‐sequencing data set. They belong to several protein families, to name a few: zinc finger proteins, solute carrier proteins, transmembrane protein family, protein phosphatases, heat shock proteins, and proteins related to the cell structure. The relatively low number of differentially regulated (2FC) transcripts following ODC treatment (N = 4 to N = 767) suggests that the observed phenotype is due to more subtle expression changes, that conjointly effectuate cell function and response to treatment. The full list of unregulated genes per cell line and common to all cell lines is included in Data Files_Unregulated genes. As for phosphoprotein regulation, depending on the cellular dynamics, phosphorylation of a given protein can be very transient (due to concerted actions of kinases and phosphatases) and therefore not detectable in any experimental setup involving a single time point. Furthermore, the ‘low’ doses used may alter the overall phosphoprofile, though in such a way that the majority of the changed proteins do not qualify for being regarded as ‘regulated’. A direct comparison of transcriptome and tyrosine phosphoproteome changes did not show any overlap, indicating that these changes are complementary rather than overlapping. However, when comparing the gene ontology analysis on differentially expressed transcripts (Fig. 5E) and the pathway analysis of the differentially expressed phosphoproteins (Fig. 6C), a clear signature relating to alteration of tyrosine kinase signaling pathways is observed in both.

Furthermore, this is in line with the *in silico* analysis of protein–protein networks that were generated based on the predicted drug targets of the different ODCs, and their first‐ and second‐order protein interactions. These networks show that targeted drugs connect to dense networks of cyclins and CDKs, and those predicted for potential downstream effects, are observed in our study.

In order to prove that our drug combination optimization is phenotype specific, we tested our cell line‐specific ODCs in freshly resected liver metastases of CRC patients. As expected, due to the environment change (therapeutic window difference between colon and liver environment), the ODCs showed some level of activity, as compared to mostly inactive chemotherapy; however, the therapeutic window was lost. This confirms that the advanced mCRC patients could be potentially treated with ODCs that would need to be optimized in an individualized way. Apart from the complex regulatory architecture, the phenotypic manifestation may very well be tissue specific and thus targeted. Therefore, specific experimental design in such cases becomes a crucial consideration for effective and safe treatment design.

## 5 Conclusions

In summary, our study established that all CRC cell lines were sensitive to growth factor receptor inhibition leading to MAP kinase shutdown. However, every cell line showed specific action and interaction of the drugs in the combination. Based on our results, we suggest that the implementation of drug combinations in the clinic could benefit from the selection of drug combinations with a high synergistic and activity potential across multiple (resistant) cell types of the same cancer. We determined in every CRC cell line dose‐specific activity and drug–drug interactions, indicating the importance of individualized drug combinations. This is in line with the dose‐specific drug interactions reported in previous studies [33], but also confirmed results from *in vivo* and clinical trials when monitoring and adapting drug dosing [59]. Lastly, our results indicate that simultaneous multitarget inhibition of important deregulated pathways has strong therapeutic potential and translational value between tumor types.

## 7 Conflict of interest

The authors are the inventors on pending (PNS, AW, EP19199136) and issued (PNS, AW, AWG, WO2015136061A3) patents on methods of drug combination therapy. Other authors have no conflict to disclose.

## 8 **Author** **contributions**

PNS, TK, LRB, PYD, AWG, and YD performed the designing research studies. MZ, GMR, MR, JRvB, AW, VM, SRP, RRdH, AA, CT, TAM, YD, and PNS performed the conducting experiments. MZ, JRvB, AW, SRP, AAH, CRJ, TVP, SR, and YD contributed to data tools/analysis. MZ, GMR, MR, JRvB, AW, VM, RHB, SRP, CDV, MD, AAH, CRJ, YD, and PNS analyzed data. MZ and PNS wrote manuscript.

9

## Supporting information


**Fig. S1.** Graphical representation of the TGMO method and study approach.
**Fig. S2.** Analysis of linear regression models.
**Fig. S3.** Drug dose‐response curves for each cell line in the TGMO screen.
**Fig. S4.** TGMO identifies DLD1‐specific ODC.
**Fig. S5.** TGMO identifies SW620‐specific ODC.
**Fig. S6.** TGMO identifies LS174T‐specific ODC.
**Fig. S7.** TGMO identifies HT29‐specific ODC.
**Fig. S8.** TGMO identifies HCT116‐specific ODC.
**Fig. S9.** TGMO identifies SW48‐specific ODC.
**Fig. S10.** ODC response surfaces and synergistic power distribution.
**Fig. S11.** Cell cycle distribution after ODC treatment at 24 and 72 hours.
**Fig. S12.** Cell morphology after ODC treatment.
**Fig. S13.** ODC validation in heterotypic 3D cultures with fibroblasts and endothelial cells
**Fig. S14.** Tumor growth curves after single drug treatments in DLD1 and SW620 tumors *in vivo*.
**Fig. S15.** DLD1‐specific ODC is effective and non‐toxic *in vivo* and accumulates in the blood.
**Fig. S16.** Analysis of morphology, proliferating cells and microvessel density in DLD1 and SW620 tumors.
**Fig. S17.** Differential gene expression analysis in HT29 and LS174T cells.
**Fig. S18.** Differential gene expression analysis in HCT116 and SW48 cells.
**Fig. S19.** INKA profiles and networks before after ODCs treatments in CRC cell lines.
**Fig. S20.** Pathway enrichment analysis.
**Fig. S21.** In silico analysis of ODCs target proteins in CRC cells.
**Fig. S22.** Cell‐specific ODC activity in patient liver metastasis and normal liver cells.

**Table S1.** The panel of CRC cell lines used in 3D cultures.
**Table S2.** Selected drugs, drug targets and clinical status.
**Table S3.** Drug plasma concentration limit (PCL) calculation table.
**Table S4.** Cell line‐specific drug doses of the ODCs in different optimization phases.
**Table S5.** Combination index of ODC activity from Search and final dose optimization.
**Table S6.** Cross‐validation of the cell‐specific ODCs across the panel of CRC cells.
**Table S7.** Single drug efficacy in DLD1 tumors *in vivo*.
**Table S8.** Single drug efficacy in SW620 tumors *in vivo*.
**Table S9.** Growth of DLD1 and SW620 sham‐treated tumors *in vivo*.
**Table S10.** Pharmacokinetics of DLD1‐specific ODC and corresponding single drugs.
**Table S11.** Pharmacokinetics of SW620‐specific ODC and corresponding single drugs.
**Table S12.** Intratumor and serum mean drug concentrations of DLD1 and SW620 tumor models at the experimental endpoint.
**Table S13.** Top 50 downregulated gene expression of ODC‐treated cells.
**Table S14.** Lysate protein.
**Table S15.** Lysate peptide.
**Table S16.** Sample labeling.
**Table S17.** pTyrIP Phosphosite.
**Table S18.** pTyrIP Phosphopeptide.
**Table S19.** Normalized spectral counts of phosphokinases.

## Data Availability

Matlab code used of the drug combination optimization and search matrices is available at Zenodo (https://doi.org/10.5281/zenodo.3956660). The RNA‐Seq data have been deposited in the NCBI Gene Expression Omnibus (www.ncbi.nlm.nih.gov/geo) and are accessible through GEO Series accession number (GSE142340). Proteomics data are available via ProteomeXchange with identifier PXD016604. The raw data are available from the corresponding author upon request.
