# Supplementary material for: Optimized low‐dose combinatorial drug treatment boosts selectivity and efficacy of colorectal carcinoma treatment
Source: Mol Oncol. 2020 Oct 5;14(11):2894–919. doi: 10.1002/1878-0261.12797 (PMC7607171; doi:10.1002/1878-0261.12797)

**Supplementary Information**

**Optimized low-dose combinatorial drug treatment boosts selectivity and efficacy of colorectal carcinoma treatment**

Marloes Zoetemelk^1,2,3^, George M. Ramzy^1,2,3^, Magdalena Rausch^1,2,3^, Thibaud Koessler^4^,

Judy R. van Beijnum^5^, Andrea Weiss^1,2^, Valentin Mieville^1,2^, Sander R. Piersma^6,7^, Richard R. de Haas^6,7^, Céline Delucinge-Vivier^8^, Axel Andres^10,11^, Christian Toso^10,11^, Alexander A. Henneman^6,7^, Simone Ragusa^12,13^, Tatiana V. Petrova^12,13^, Mylène Docquier^8,9^, Thomas A. McKee^14^, Connie R. Jimenez^6,7^, Youssef Daali^15^, Arjan W. Griffioen^5^, Laura Rubbia-Brandt^14^, Pierre-Yves Dietrich^3,4^,

Patrycja Nowak-Sliwinska^1,2,3^*

^1^ Molecular Pharmacology Group, School of Pharmaceutical Sciences, University of Geneva, Geneva, Switzerland

^2^ Institute of Pharmaceutical Sciences of Western Switzerland, University of Geneva, Geneva, Switzerland

^3^ Translational Research Center in Oncohaematology, Geneva, Switzerland

^4^ Department of Oncology, Geneva University Hospitals and Faculty of Medicine, Geneva, Switzerland

^5^ Angiogenesis Laboratory, Department of Medical Oncology, Cancer Center Amsterdam, Amsterdam UMC-location VUmc, VU University Amsterdam, Amsterdam, The Netherlands

^6^ Department of Medical Oncology, Cancer Center Amsterdam, Amsterdam UMC, Vrije Universiteit Amsterdam, Amsterdam, The Netherlands

^7^ OncoProteomics Laboratory, Cancer Center Amsterdam, Amsterdam UMC, Vrije Universiteit Amsterdam, Amsterdam, The Netherlands

^8^ iGE3 Genomics Platform, University of Geneva, Switzerland

^9^ Department of Genetics & Evolution, University of Geneva, Switzerland

^10^ Translational Department of Digestive and Transplant Surgery, Geneva University Hospitals and Faculty of Medicine, Geneva, Switzerland

^11^ Hepato-Pancreato-Biliary Centre, Geneva University Hospitals and Faculty of Medicine, Geneva, Switzerland

^12^ Department of Oncology, University of Lausanne, Lausanne, Switzerland

^13^ Ludwig Institute for Cancer Research Lausanne, Lausanne, Switzerland

^14^ Division of Clinical Pathology, Diagnostic Department, University Hospitals of Geneva (HUG), Geneva, Switzerland

^15^ Division of Clinical Pharmacology and Toxicology, Department of Anaesthesiology, Pharmacology, Intensive Care and Emergency Medicine, Geneva University Hospitals, Geneva, Switzerland

#

# Supplementary Figures


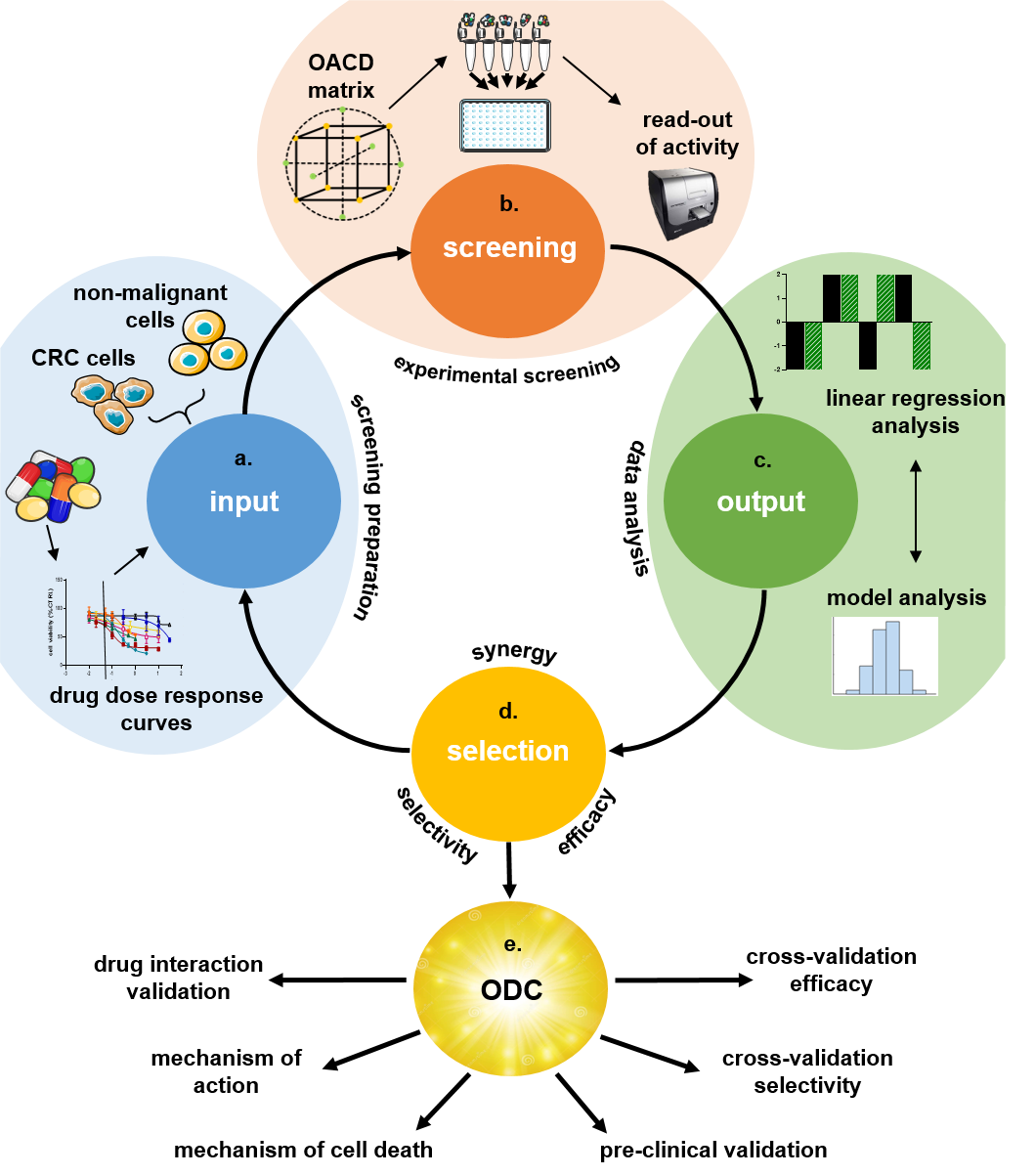


## Supplementary Figure S1. Graphical representation of the TGMO method and study approach

**a.** The input selection of an appropriate cell system (panel of CRC cells and non-malignant colon epithelial CCD841CoN cells), together with the drug/dose selection defined from drug dose-response curves. **b.** Experimental screening of the selected drugs and doses in the cells according to the OACD matrix with as output drug combination inhibition of cell metabolic activity with reader-based technology. **c.** Output modeling with step-wise linear regression analysis of both the activity on CRC cells and the therapeutic window (defined as the difference in activity between the CRC cell and the non-malignant cells), resulting in opposite models informing on effects from both the individual drugs and drug-drug interactions. Variance and predictive accuracy of the models are evaluated using various statistical tests. **d.** Drug selection and elimination of active, selective and synergistic drug combinations. **e.** Optimal drug combination validation and translation.


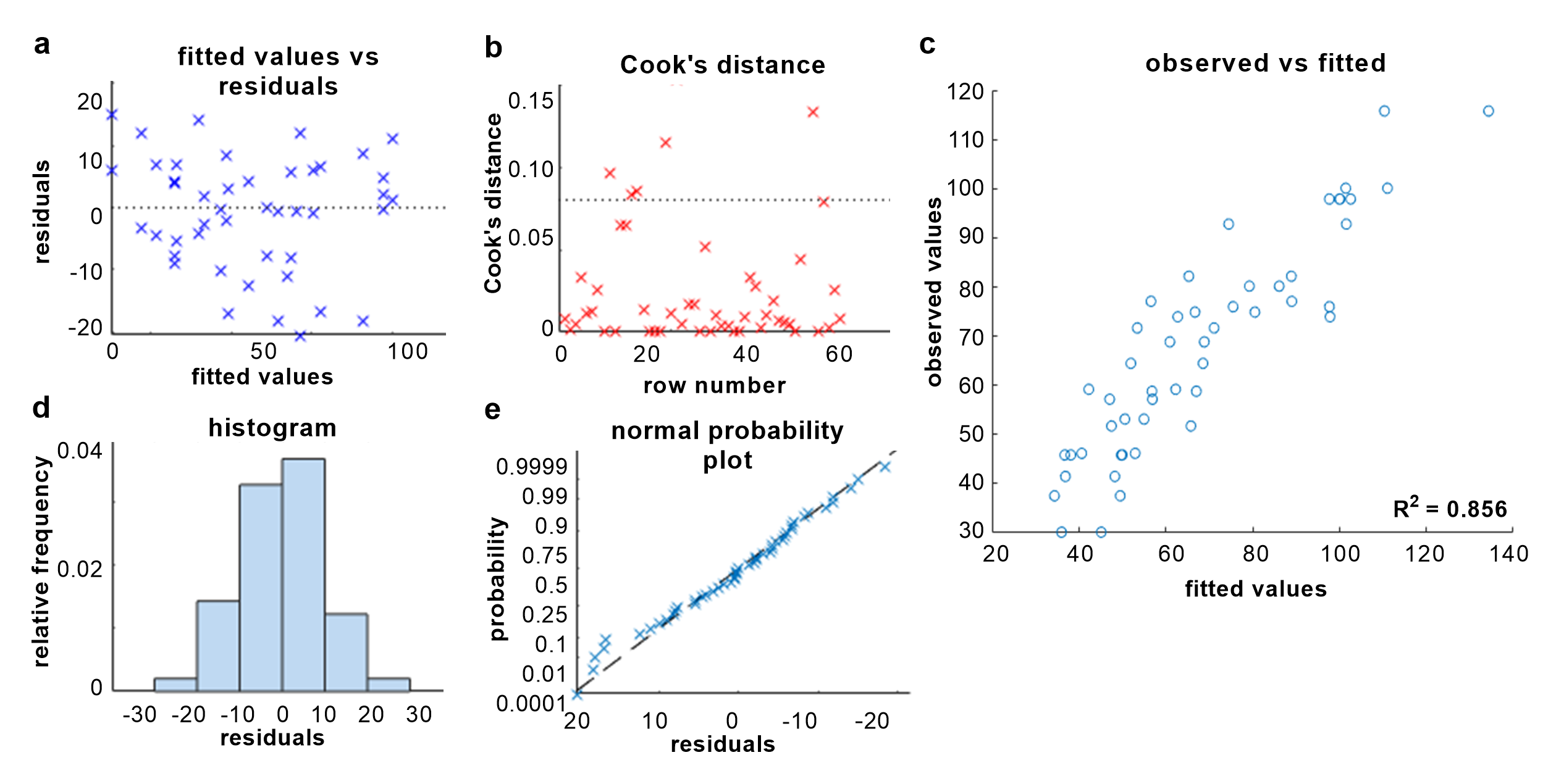


## Supplementary Figure S2. Analysis of linear regression models

Model analysis to evaluate the accuracy and predictive value of the models, illustrating the model analysis of *Search 3* in SW620. **a.** Residual analysis plot of data to visualize constant variance, **b**. Cook’s distance plot to identify influential outliers in a set of predictor variables, **c**. observed vs. fitted values plot with the multiple determination (R^2^) assessing fitted accuracy and lack of multicolinearity, **d.** histogram of residuals to appraise normal distribution of variance, and **e**. Q-Q plot to visualize independence of errors.


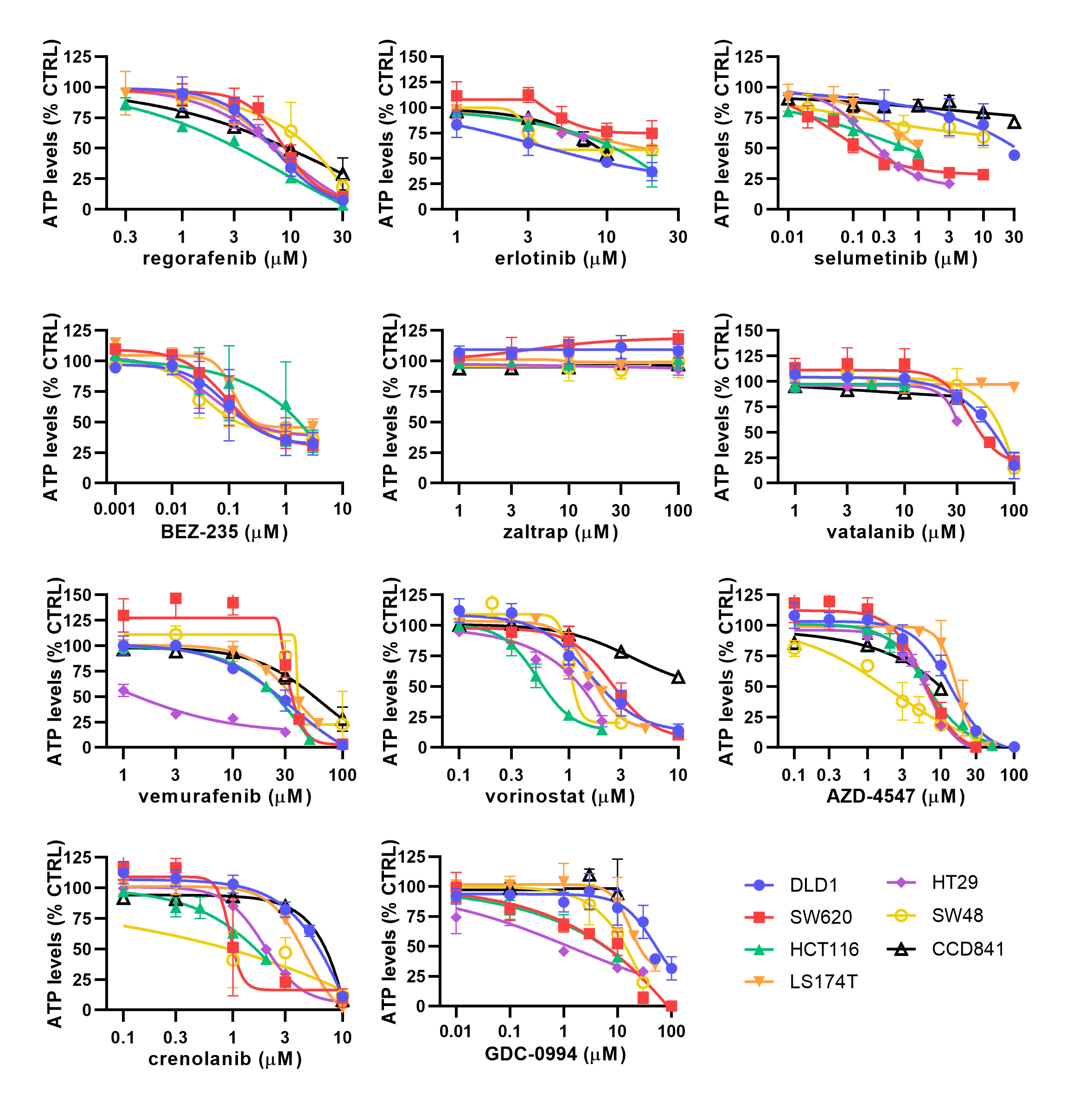


## Supplementary Figure S3. Drug dose-response curves for each cell line in the TGMO screen

Drug dose-response curves for regorafenib, erlotinib, selumetinib, BEZ-235, zaltrap, vatalanib, vemurafenib, Vorinostat, AZD-4547, crenolanib and GDC-0994. A four-parameter non-linear fit was applied to the data, with exception of a bell-shaped non-linear fit curve for erlotinib in SW620 and an asymmetric five parameter non-linear fit curve for vemurafenib in SW620 and SW48 cells, using Graphpad Prism^®^.


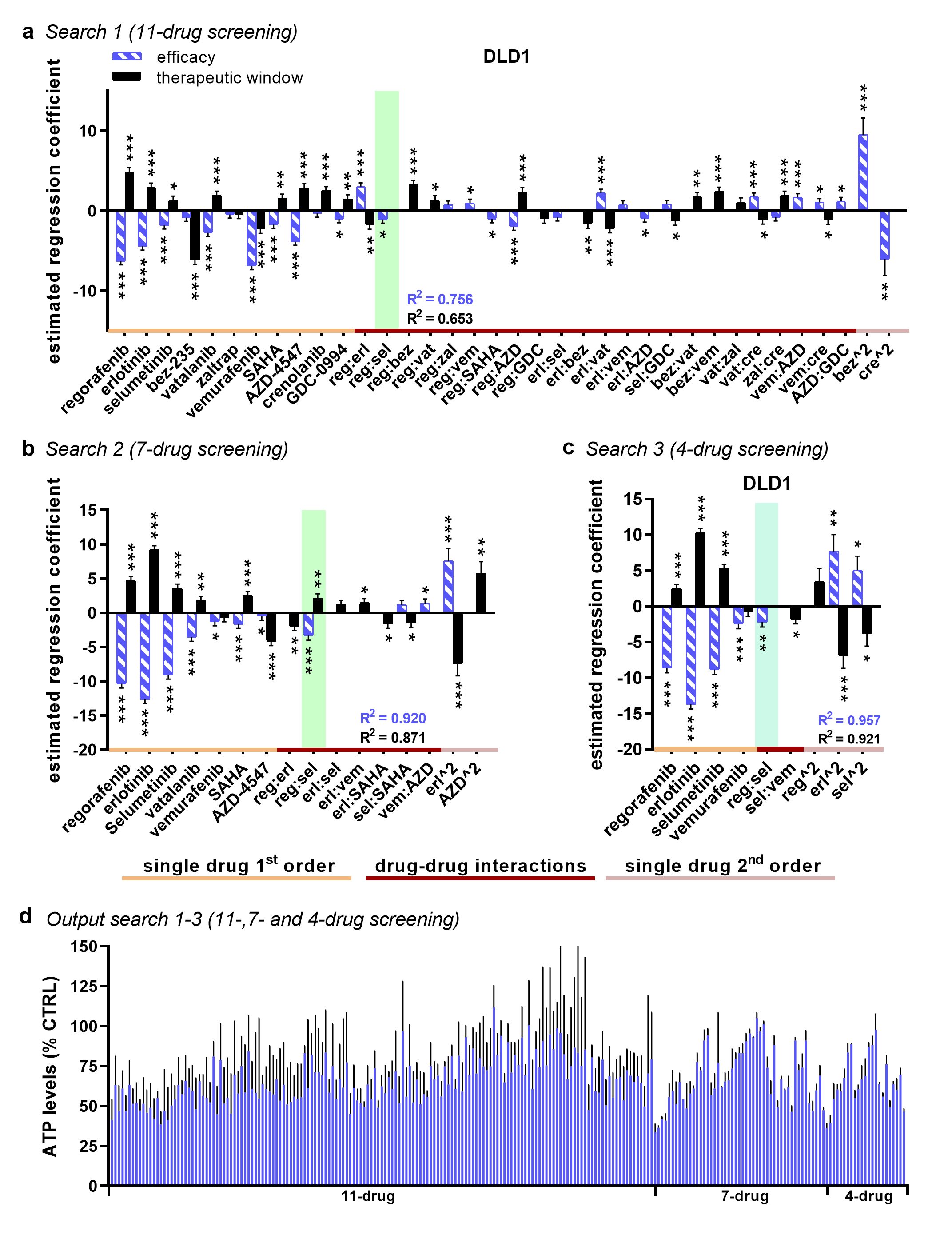


## Supplementary Figure S4. TGMO identifies DLD1-specific ODC

Estimated regression coefficients describing single drug 1^st^ order, drug-drug interactions and single drug 2^nd^ order activity of the drugs in the screening performed in DLD1 cells (blue striped bars) and non-malignant (CCD841 CoN) cells to create a therapeutic window (black bars). In green is highlighted the most interesting or robust synergistic interactions selected in the final drug combinations. **a.** *Search 1*, screening of 155 drug combinations with 11 drugs (n=3). **b.** *Search 2*, screening of 50 drug combinations with 7 drugs (n=2). **c.** *Search 3*, screening of 25 drug combinations with 4 drugs (n=2). **d.** Drug combination activity in ATP levels as a measure of metabolic activity and cell viability versus CTRL (0.15% DMSO) from the complete screening. Error bars represent the SD and significance of estimated regression coefficients was determined with a one-way ANOVA and is represented with *p < 0.05, **p < 0.01 and 0.01 < ***p < 0.001. R^2^ represents model accuracy in a coefficient of multiple determination.


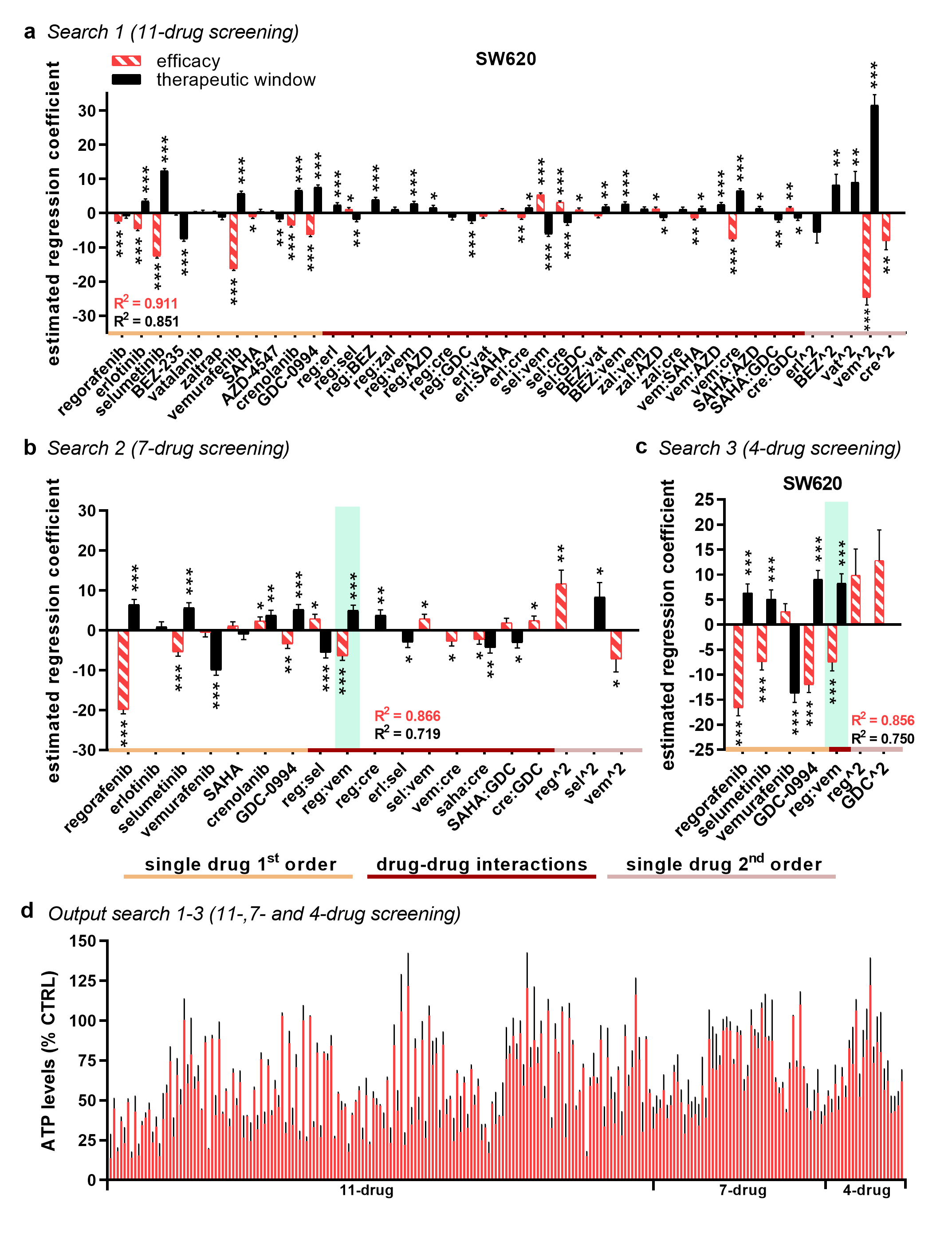


## Supplementary Figure S5. TGMO identifies SW620-specific ODC

Estimated regression coefficients describing single drug 1^st^ order, drug-drug interactions and single drug 2^nd^ order activity of the drugs in the screening performed in SW620 cells (blue striped bars) and non-malignant (CCD841 CoN) cells to create a therapeutic window (black bars). In green is highlighted the most interesting or robust synergistic interactions selected in the final drug combinations. **a.** *Search 1*, screening of 155 drug combinations with 11 drugs (n=2). **b.** *Search 2*, screening of 50 drug combinations with 7 drugs (n=2). **c.** *Search 3*, screening of 25 drug combinations with 4 drugs (n=2). **d.** Drug combination activity in ATP levels as a measure of metabolic activity and cell viability versus CTRL (0.15% DMSO) from the complete screening. Error bars represent the SD and significance of estimated regression coefficients was determined with a one-way ANOVA and is represented with *p < 0.05, **p < 0.01 and 0.01 < ***p < 0.001. R^2^ represents model accuracy in a coefficient of multiple determination.


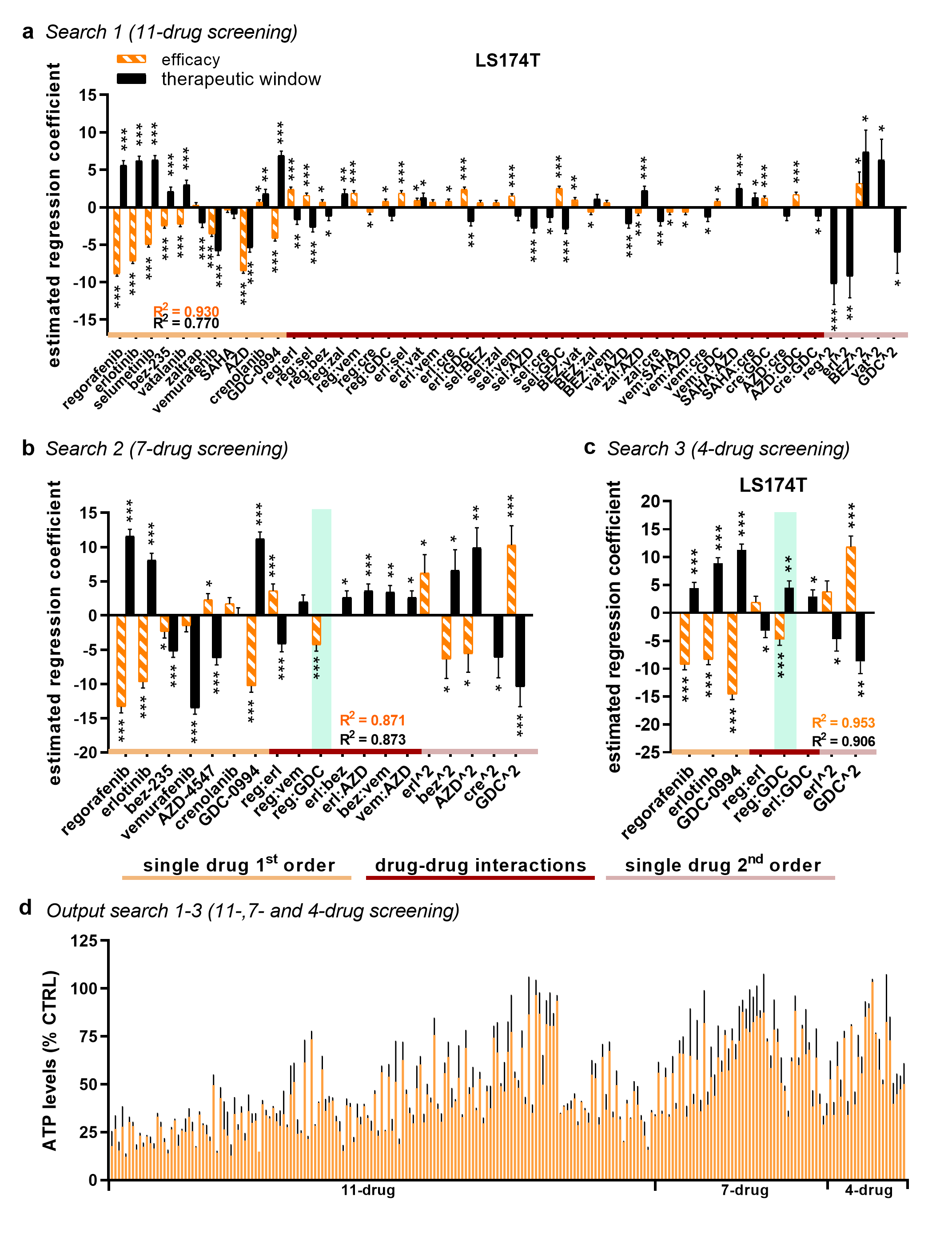


## Supplementary Figure S6. TGMO identifies LS174T-specific ODC

Estimated regression coefficients describing single drug 1^st^ order, drug-drug interactions and single drug 2^nd^ order activity of the drugs in the screening performed in LS174T cells (blue striped bars) and non-malignant (CCD841 CoN) cells to create a therapeutic window (black bars). In green is highlighted the most interesting or robust synergistic interactions selected in the final drug combinations. **a.** *Search 1*, screening of 155 drug combinations with 11 drugs (n=2). **b.** *Search 2*, of 50 drug combinations with 7 drugs (n=2). **c.** *Search 3*, screening of 25 drug combinations with 4 drugs (n=3). **d.** Drug combination activity in ATP levels as a measure of metabolic activity and cell viability versus CTRL (0.15% DMSO) from the complete screening. Error bars represent the SD and significance of estimated regression coefficients was determined with a one-way ANOVA and is represented with *p < 0.05, **p < 0.01, and 0.01 < ***p < 0.001. R^2^ represents model accuracy in a coefficient of multiple determination.


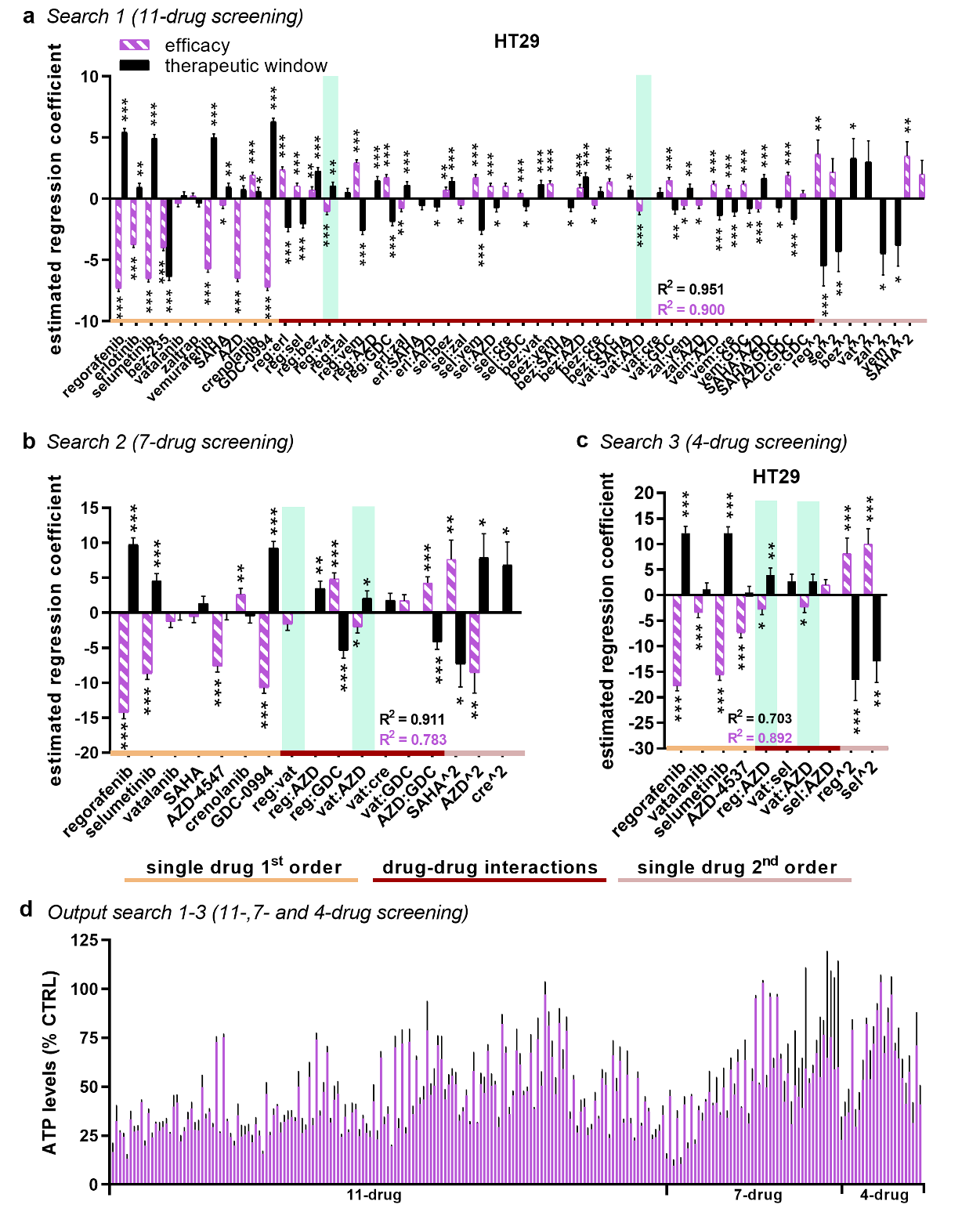


## Supplementary Figure S7. TGMO identifies HT29-specific ODC

Estimated regression coefficients describing single drug 1^st^ order, drug-drug interactions and single drug 2^nd^ order activity of the drugs in the screening performed in HT29 cells (blue striped bars) and non-malignant (CCD841 CoN) cells to create a therapeutic window (black bars). In green is highlighted the most interesting or robust synergistic interactions selected in the final drug combinations. **a.** *Search 1*, screening of 155 drug combinations with 11 drugs (n=3). **b.** *Search 2*, screening of 50 drug combinations with 7 drugs (n=2). **c.** *Search 3*, screening of 25 drug combinations with 4 drugs (n=4). **d.** Drug combination activity in ATP levels as a measure of metabolic activity and cell viability versus CTRL (0.15% DMSO) from the complete screening. Error bars represent the SD and significance of estimated regression coefficients was determined with a one-way ANOVA and is represented with *p < 0.05, **p < 0.01 and 0.01 < ***p < 0.001. R^2^ represents model accuracy in a coefficient of multiple determination.


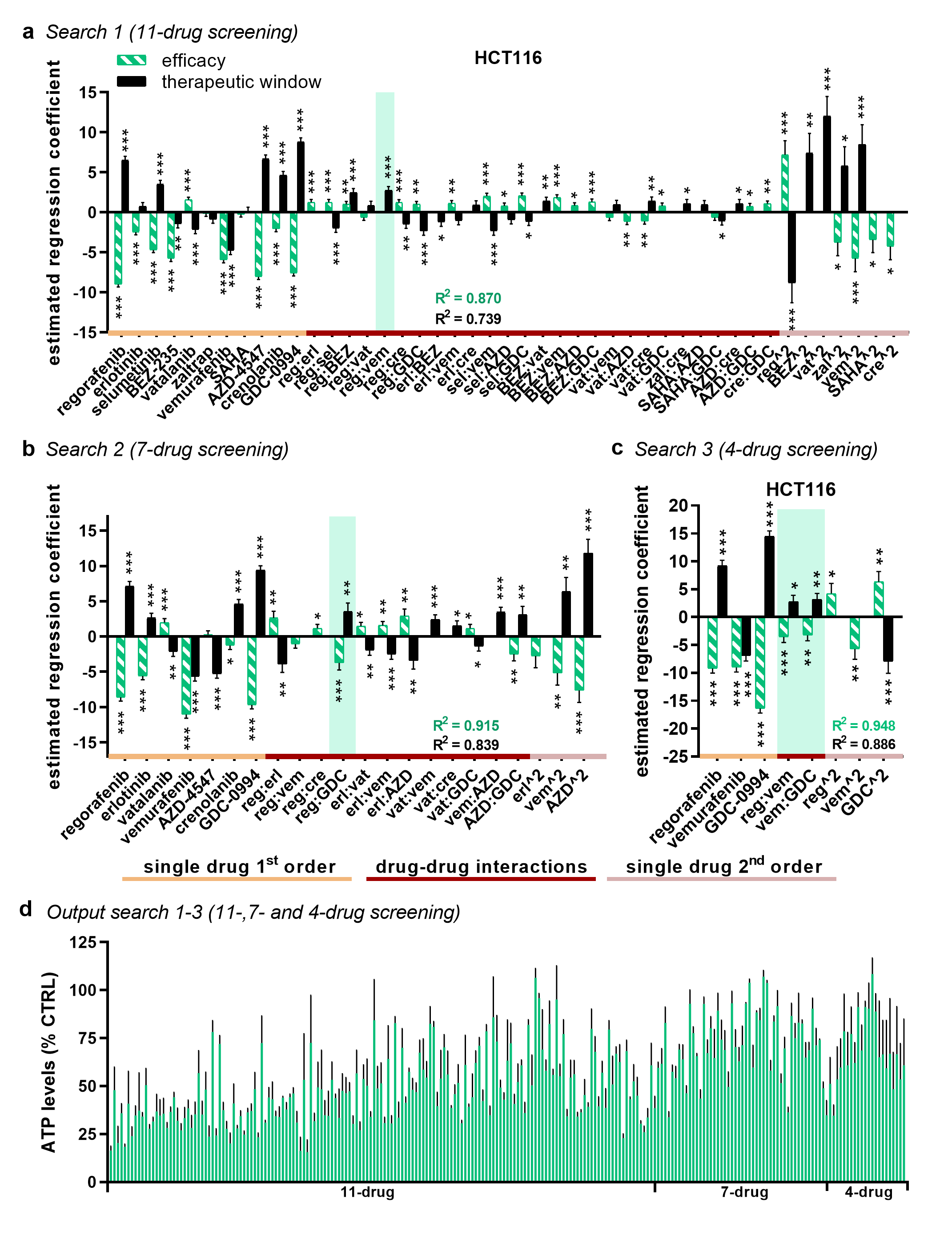


## Supplementary Figure S8. TGMO identifies HCT116-specific ODC

Estimated regression coefficients describing single drug 1^st^ order, drug-drug interactions and single drug 2^nd^ order activity of the drugs in the screening performed in HCT116 cells (blue striped bars) and non-malignant (CCD841 CoN) cells to create a therapeutic window (black bars). In green is highlighted the most interesting or robust synergistic interactions selected in the final drug combinations. **a.** *Search 1*, screening of 155 drug combinations with 11 drugs (n=3). **b.** *Search 2*, screening of 50 drug combinations with 7 drugs (n=3). **c.** *Search 3*, screening of 25 drug combinations with 4 drugs (n=3). **d.** Drug combination activity in ATP levels as a measure of metabolic activity and cell viability versus CTRL (0.15% DMSO) from the complete screening. Error bars represent the SD and significance of estimated regression coefficients was determined with a one-way ANOVA and is represented with *p < 0.05, **p < 0.01 and 0.01 < ***p < 0.001. R^2^ represents model accuracy in a coefficient of multiple determination.


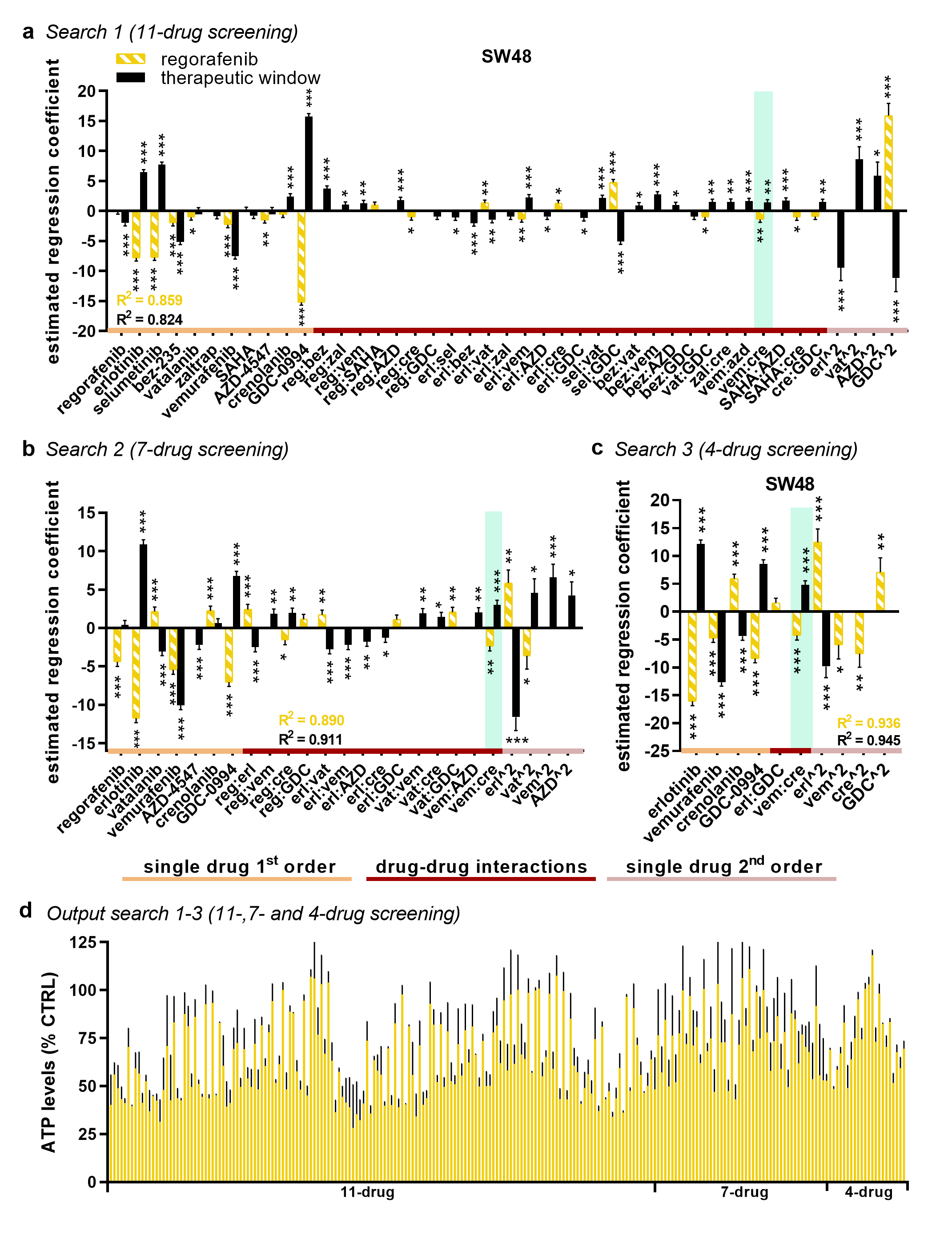


## Supplementary Figure S9. TGMO identifies SW48-specific ODC

Estimated regression coefficients describing single drug 1*st* order, drug-drug interactions and single drug 2^nd^ order activity of the drugs in the screening performed in SW48 cells (blue striped bars) and non-malignant (CCD841 CoN) cells to create a therapeutic window (black bars). In green is highlighted the most interesting or robust synergistic interactions selected in the final drug combinations. **a.** *Search 1*, screening of 155 drug combinations with 11 drugs (n=2). **b.** *Search 2*, screening of 50 drug combinations with 7 drugs (n=4). **c.** *Search 3*, screening of 25 drug combinations with 4 drugs (n=2). **d.** Drug combination activity in ATP levels as a measure of metabolic activity and cell viability versus CTRL (0.15% DMSO) from the complete screening. Error bars represent the SD and significance of estimated regression coefficients was determined with a one-way ANOVA and is represented with *p < 0.05, **p < 0.01 and 0.01 < ***p < 0.001. R^2^ represents model accuracy in a coefficient of multiple determination.


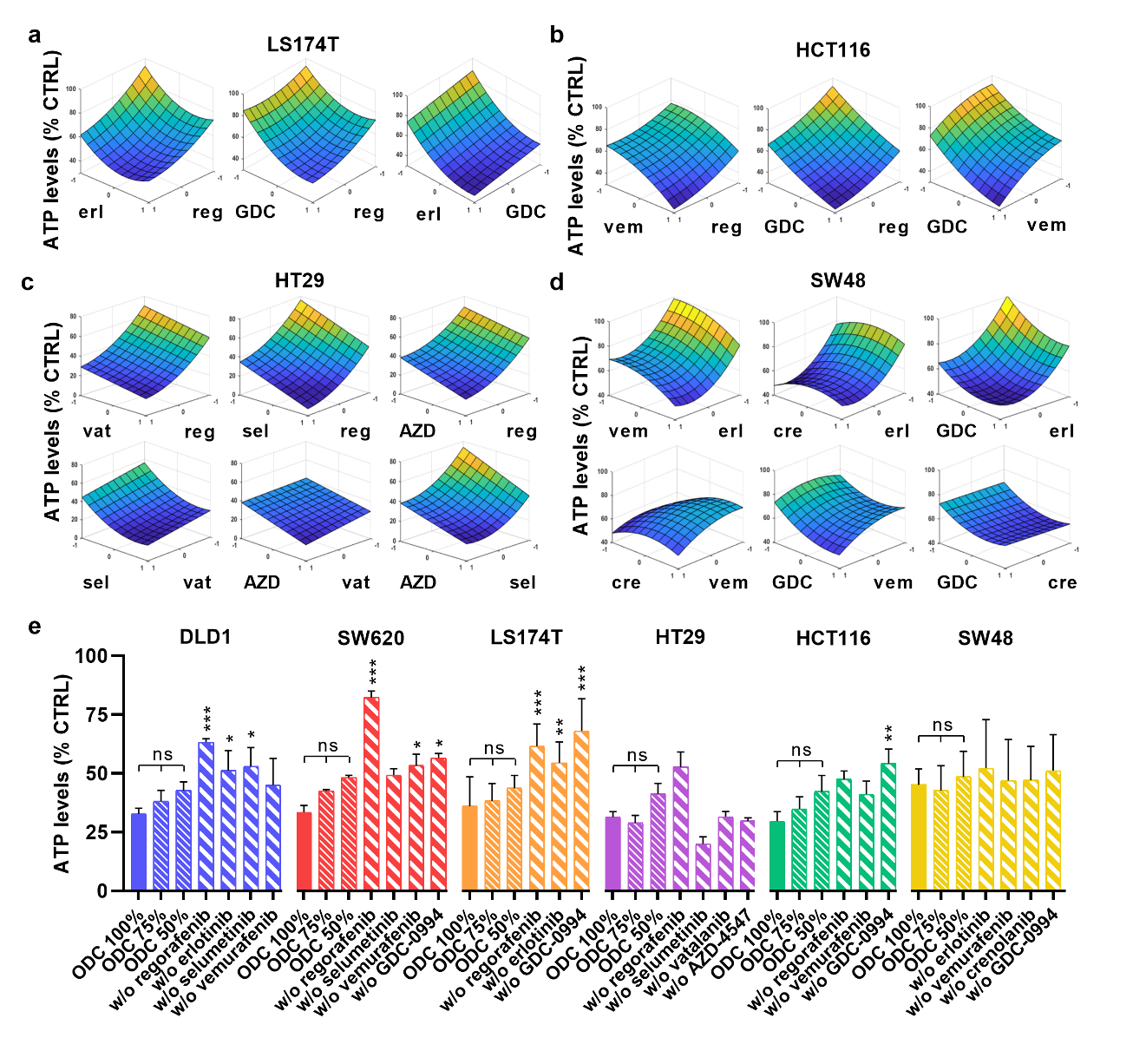


## Supplementary Figure S10. ODC response surfaces and synergistic power distribution

Response surface contour plots between all two-drug options after treatment with the cell-specific ODCs of **a.** LS174T, **b.** HCT116, **c.** HT29 and **d.** SW48 in *Search 3*, fitted with a step-wise second-order linear regression model. The y-axis represents drug activity (ATP levels, % CTRL), the x-axis’ represent the dose range (1, high dose, ED_20_; 0, low dose, ED_10_; -1, no drug) for each of the drugs. Abbreviations: reg, regorafenib; erl, erlotinib; sel, selumetinib; vem, vemurafenib; GDC, GDC-0994; vat, vatalanib; AZD, AZD-4547; cre, crenolanib. **e.** Efficacy in cell metabolic activity (ATP levels, % CTRL) of the ODC at 100%, with all drug doses, simultaneously decreased to 75% or 50% of the full dose, or by removing a drug from the ODCs, in N=2-4 independent experiments. Error bars represent the SD and significances of *p < 0.05, **p < 0.01 and ***p < 0.001 represent the comparison with the ODC at 100% dosing as determined by a two-way ANOVA with post hoc Dunnett’s multiple comparisons test.


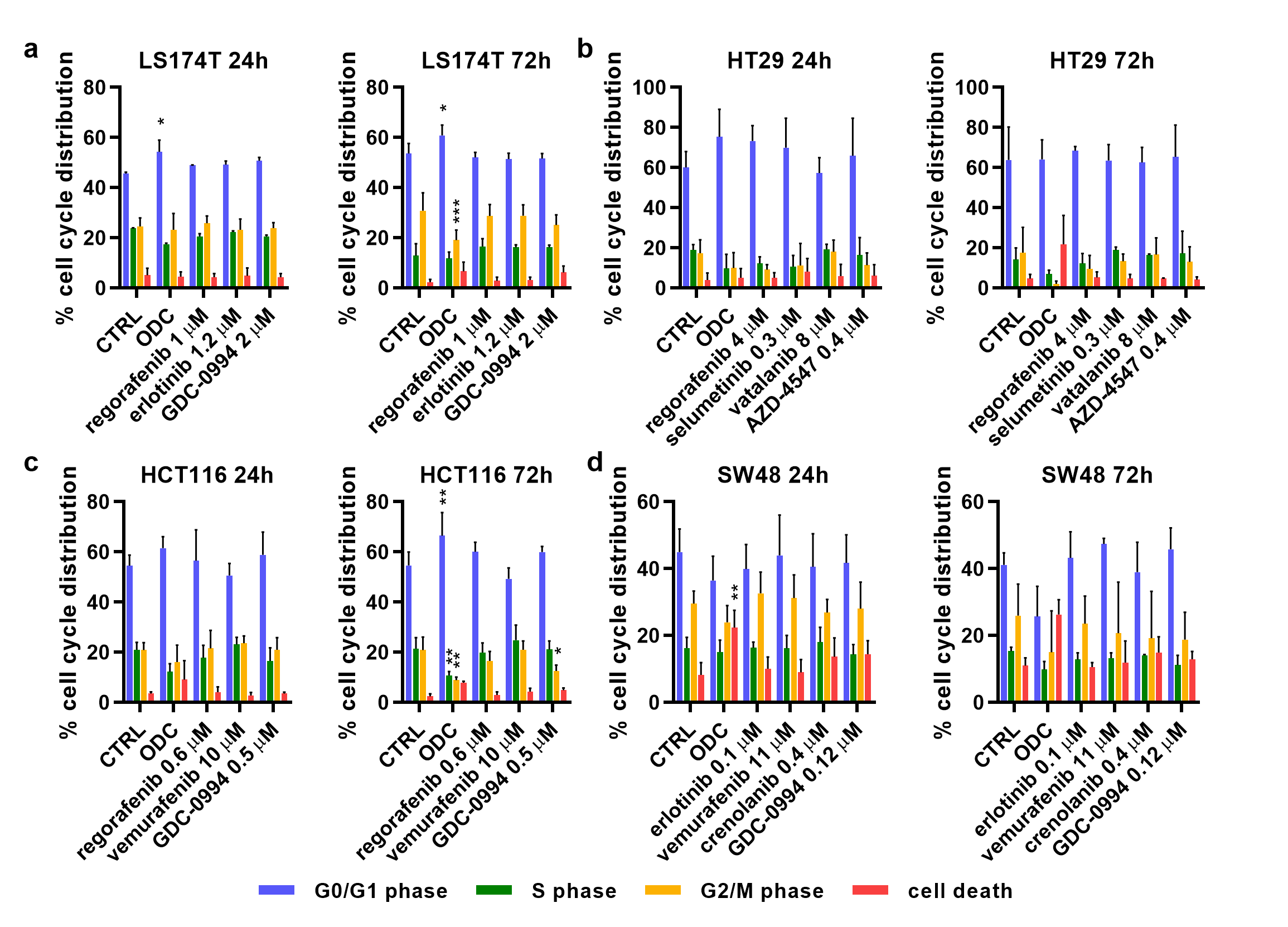


## Supplementary Figure S11. Cell cycle distribution after ODC treatment at 24 and 72 hours

Cell cycle distribution (G0/G1, S, G2/M phases or cell death) of **a.** LS174T, **b.** HT29, **c.** HCT116 and **d.** SW48 cells after 24h (left graph) or 72h (right graph) treatment with the ODCs, corresponding monotherapies or CTRL (0.15% DMSO). Independent experiments conducted per cell line for 24h and 72h, respectively, is N=2 and 4 for LS174T, N=4 and 2 for HT29, N=5 and 3 for HCT116 and N=4 and 2 for SW48. Error bars represent the SD and significances of *p < 0.05, **p < 0.01 and ***p < 0.001 represent the comparison with the CTRL as determined by a two-way ANOVA with post hoc Dunnett’s multiple comparisons test.


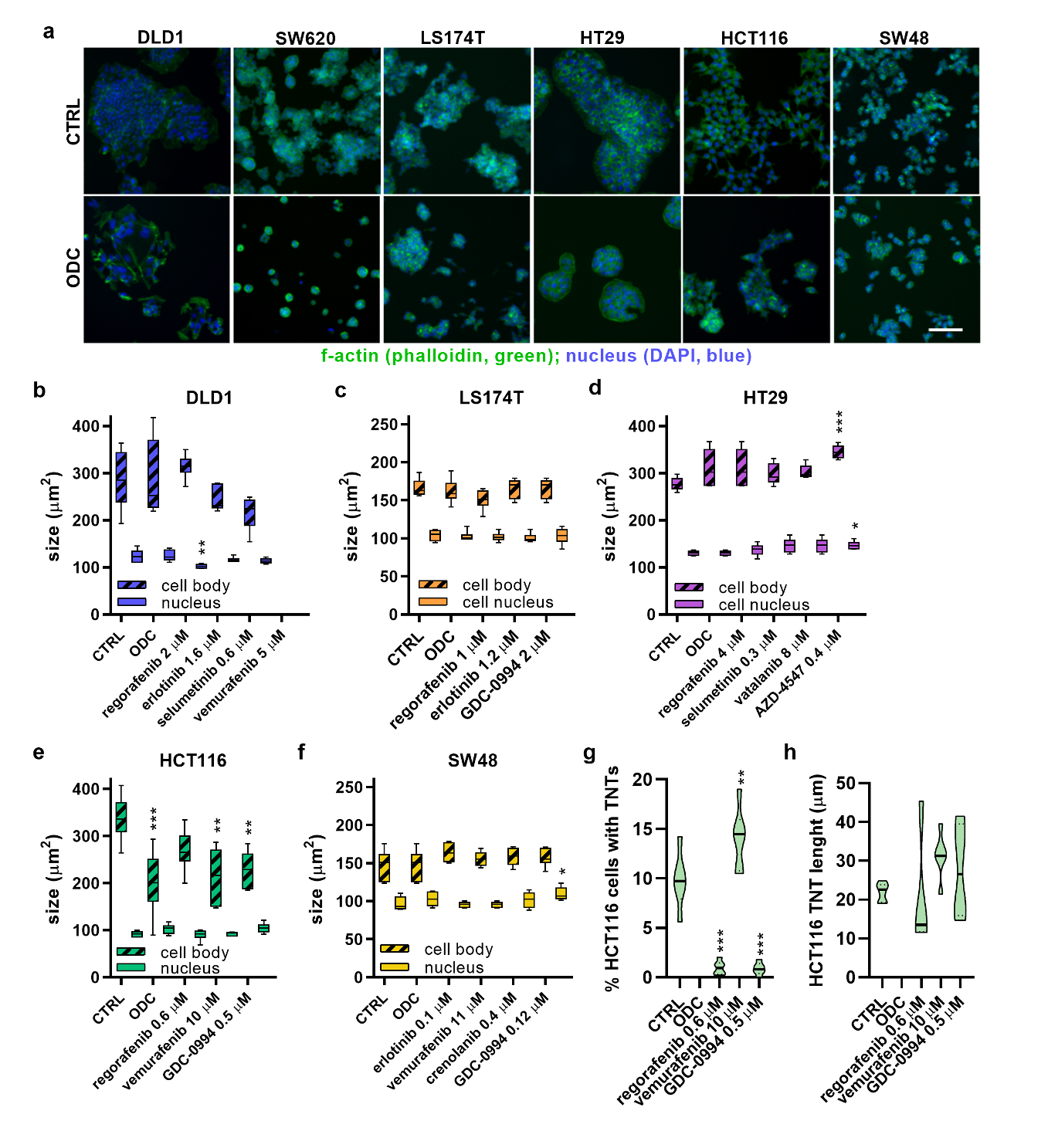


## Supplementary Figure S12. Cell morphology after ODC treatment

**a.** CRC cells after staining for f-actin (phalloidin, green) and the nucleus (DAPI, blue). Scale bar represents 100 µm. Boxplots of image-based quantification of cell size (top bars) and nucleus size (bottom bars) of cells treated with the ODC, corresponding monotherapies or the CTRL (0.15% DMSO) of **b.** DLD1, **c.** LS174T, **d.** HT29, **e.** HCT116 and **f.** SW48 cells of N=2 experiments. Violin plots for quantification of **f.** percentage and **g.** length of tunneling nanotubules (TNTs) of HCT116 cells treated with the ODC, corresponding monotherapies or the CTRL of N=2 independent experiments, n=6. Error bars represent the SD and significances of *p < 0.05, **p < 0.01 and ***p < 0.001 represent the comparison with the CTRL as determined by a one-way ANOVA with post hoc Dunnett’s multiple comparisons test.


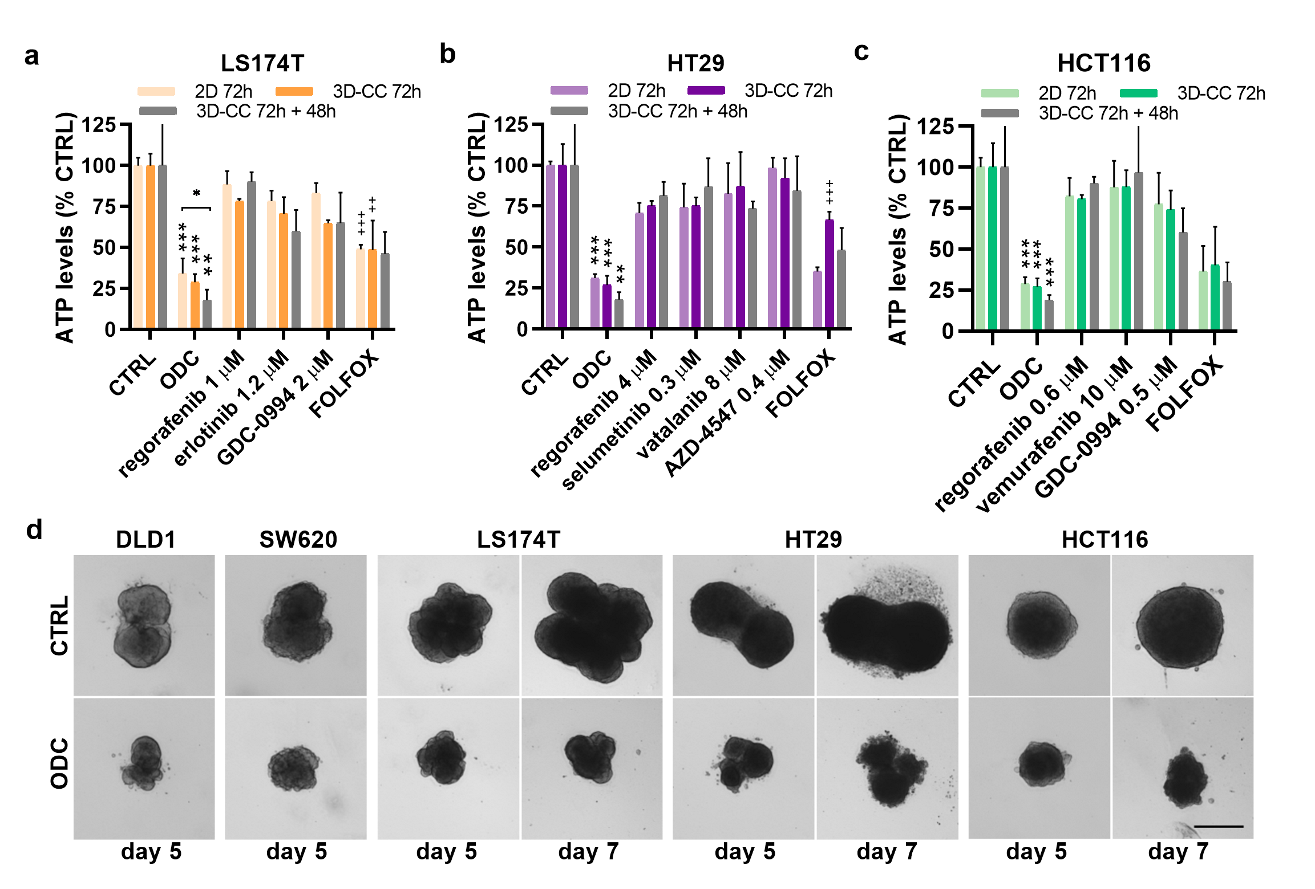


## Supplementary Figure S13. ODC validation in heterotypic 3D cultures with fibroblasts and endothelial cells

Efficacy in cell metabolic activity (ATP levels, % CTRL) of the ODC, corresponding monotherapies, FOLFOX and CTRL (0.15% DMSO) of **a.** LS174T, **b.** HT29 and **c.** HCT116 CRC cells. 2D cultures were treated on day 1 post-seeding for 72h. Heterotypic 3D co-cultures (3D-CCs) consisted of CRC cells in ratio 1:1 with healthy colon CCD841CoN fibroblasts and 5% ECRF24 endothelial cells (500:500:50 cells), and were treated on day 2 for 72h or 72h+48h. Error bars represent the SD of N=2-3 experiments and significance of *p < 0.05, **p < 0.01 and ***p < 0.001, and ^++^p < 0.01 and ^+++^p < 0.001 represent the comparison of the ODC with the CTRL and corresponding monotherapies or ODC with FOLFOX, respectively. Independent experiments conducted for 72h and 72h+48h, respectively: N=3 and 2 (LS174T), N=3 and 2 (HT29) and N=3 and 2 (HCT116). **d.** Representative images of heterotypic 3D-CCs after 72h (day 5) or 72h+48h (day 7) of treatment. Scale bar represents 400 µm.


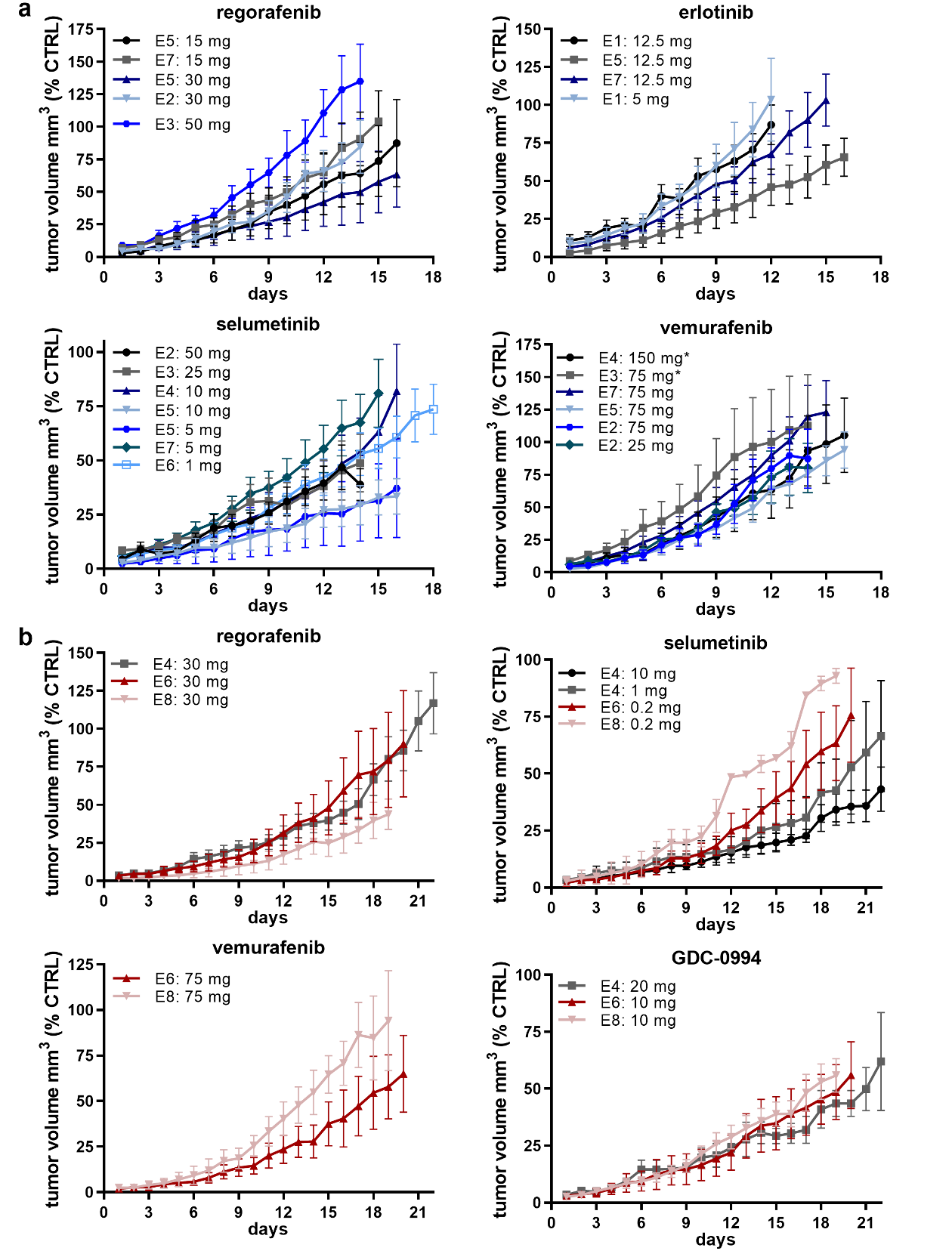


## Supplementary Figure S14. Tumor growth curves after single drug treatments in DLD1 and SW620 tumors *in vivo*

Tumor growth curves in volume mm^3^ (% CTRL, sham) *in vivo* after single drug treatments **a.** for DLD1 with regorafenib, erlotinib, selumetinib and vemurafenib and **b.** for SW620 with regorafenib, selumetinib, vemurafenib and GDC-0994. Various doses were tested to determine the sub-optimal dose-response in experiments 1-4 (E1-E4, see **Supplementary Table S7**). The corresponding monotherapies of the ODCs tested in experiment 5-8 (E5-E8, see **Supplementary Table S8**) are also included to visualize intra-experimental performance. The doses given represent the administration in mg/kg. *b.i.d.


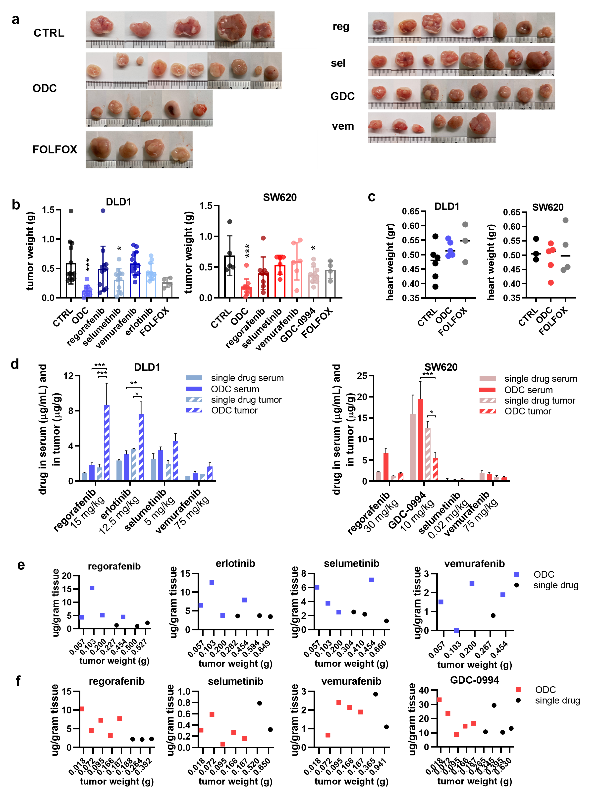


## Supplementary Figure S15. DLD1-specific ODC is effective and non-toxic *in vivo* and accumulates in the blood

**a.** Representative images of subcutaneously implanted SW620 tumors in Swiss Nu/nu mice after 19 days of daily treatment with CTRL, ODC and FOLFOX and corresponding monotherapies. **b.** Tumor weight and **c.** % heart weight of mouse body weight after resection of DLD1 and SW620 tumors, respectively. Independent points for control, ODC and FOLFOX, respectively includes: N=6, N=5 and N=3 for DLD1 and N=3, N=5 and N=4 for SW620. **d.** Drug concentration in serum (ng/mL) and tumor (ng/g) 1h post-treatment with the ODC or corresponding monotherapies at experiment endpoint for DLD1 and SW620 murine tumor models. Independent points for monotherapies and ODCs, respectively, includes: N=3 and N=5 for DLD1 and N=3-4 and N=4-5 for SW620. **e,f**. Drug concentrations plotted over increasing tumor weight of ODC or single drug-treated DLD1 or SW620 tumors, respectively, to visualize lack of weight to concentration correlation. Error bars represent the SD and significance of *p < 0.05, **p < 0.01 and ***p < 0.001 represent the comparison with the sham CTRL (**b,c**) or ODC vs single drug and tumor vs. serum (**d**) using a one-way (**b,c**) or two-way (**d**) ANOVA with post hoc Dunnett’s multiple comparisons test.


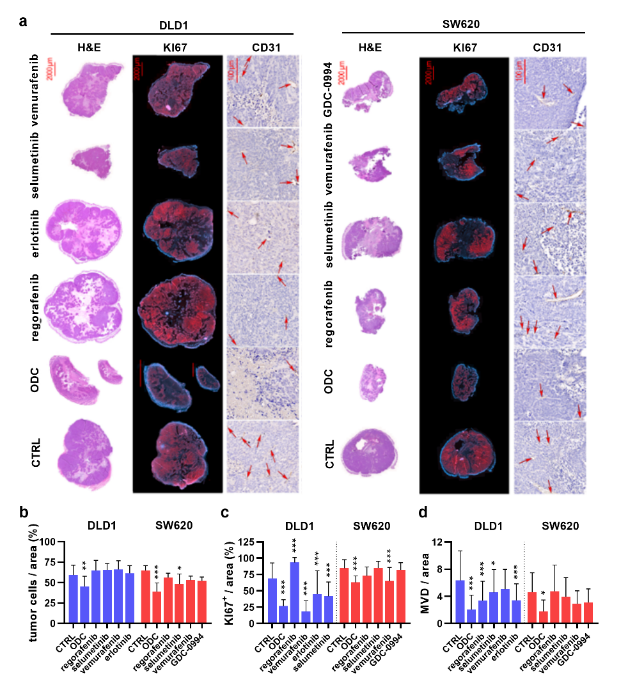


## Supplementary Figure S16. Analysis of morphology, proliferating cells and microvessel density in DLD1 and SW620 tumors

**a.** Representative images of DLD1 and SW620 tumor sections stained for morphology and tumor heterogeneity (H&E, first column), proliferating cell nuclei (KI67, second column) and blood vessels (endothelial marker CD31, third column, arrows depict CD31^+^ staining). **b.** Percentage of tissue with tumor cells identified with H&E staining. **c.** Percentage of tissue with Ki67^+^ tumor cells. **d.** Total microvessel density (MVD, number of CD31^+^ cells), **e.** MVD / 300 µm^2^ divided in diameter with a threshold of 50 µm, and **f.** MVD divided in morphology with open or closed lumen. Scalebar images whole tissues= 2,000 µm, scalebar images zoomed in = 100 µm, area/image quantified = 300 µm^2^. Error bars represent the SD and significance of *p < 0.05, **p < 0.01 and ***p < 0.001 represent the comparison with the CTRL using a one-way (**b,c,d**) or two-way (**e,f**) ANOVA with post hoc Dunnett’s (**b,c,d**) or Sidak’s (**e,f**) multiple comparisons test.


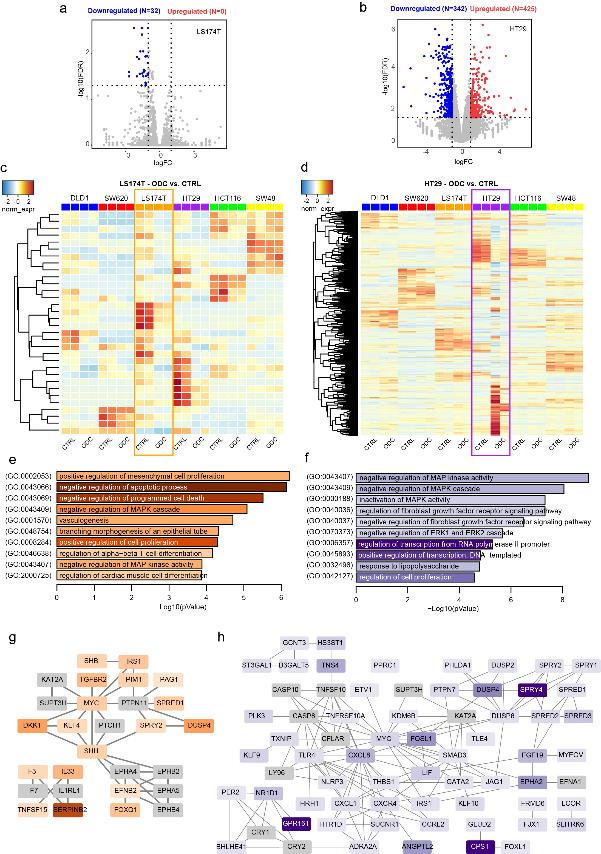


## Supplementary Figure S17. Differential gene expression analysis in LS174T and HT29 cells

Differential gene expression analysis was performed on from RNA-sequencing (RNA-seq) data of ODC-treated CRC cells relative to CTRL (0.15% DMSO) of N=2 (n=4) independent experiments. **a,b.** Vulcano plots of significant genes (p-value < 0.05) and a fold change > 2 (logFC) in LS174T and HT29 cells, respectively. Color-coding is based on the fold change (red = significant upregulated genes, blue = significant downregulated genes). Vertical lines highlight log2 fold changes of -1 and +1, while a horizontal line represents a corrected for multiple test p-value of 0.05. **c**,**d.** Heatmap of genes differentially up- and down-regulated after 2h of ODC treatment compared to CTRL in LS174T and HT29 cells, respectively. **e,f**. Enrichment analysis of downregulated genes in LS174T (N=32) and HT29 (N=103; >3FC) for Gene Ontology – Biological Process. The top 10 functional clusters are sorted according to p-value and color intensity is proportional to the number of represented genes per ontology. **g,h**. Protein interaction analysis of downregulated genes in LS174T and HT29 cells, respectively. Using STRING (string-db.org) an enhanced network up to 10 interactors per 1^st^ order shell was performed**.** Only connected nodes are shown. Nodes in grey are 1^st^ order interactors of the differentially expressed genes, which are color-coded proportionally to fold change.


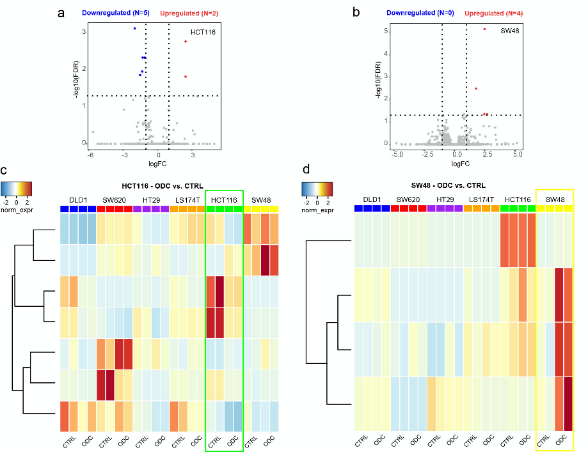


## Supplementary Figure S18. Differential gene expression analysis in HCT116 and SW48 cells

Differential gene expression analysis was performed on from RNA-sequencing (RNA-seq) data of ODC-treated CRC cells relative to CTRL (0.15% DMSO) of N=2 (n=4) independent experiments. **a,b.** Vulcano plots of significant genes (p-value < 0.05) and a fold change > 2 (logFC) in HCT116 (N=7) and SW48 (N=4) cells, respectively. Color-coding is based on the fold change (red = significant upregulated genes, blue = significant downregulated genes). Vertical lines highlight log2 fold changes of -1 and +1, while a horizontal line represents a corrected for multiple test p-value of 0.05. **c,d.** Heatmap of genes differentially up- and down-regulated after 2h of ODC treatment compared to CTRL in HCT116 and SW48 cells, respectively.


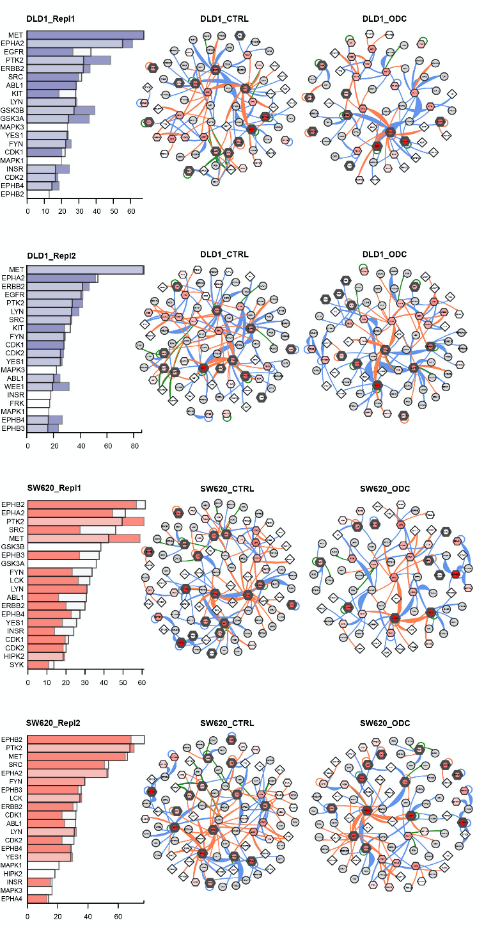


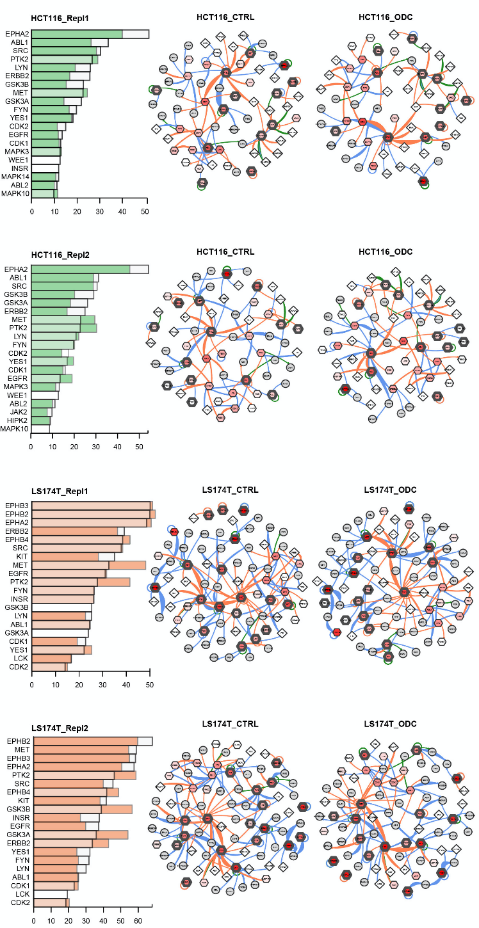


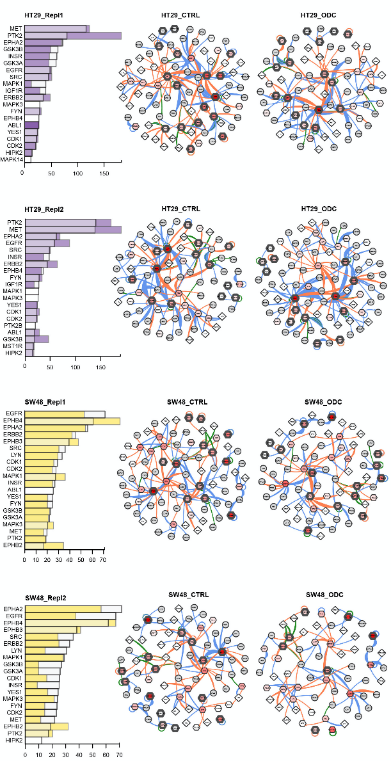


## **Supplementary Figure S19. INKA profiles and networks before after ODCs treatments in CRC cell** lines

INKA ranking plots of two replicate samples for treated and untreated in CRC cell lines (N=2). Kinases targeted by drugs are color-coded according to their respective legends. These plots allow for the relative ranking of kinases before and after treatment. Bars are darker colored if INKA score after ODC treatment was lower. If the INKA score was higher after ODC, the darker bar representing ODC INKA is placed behind the white bar. Uncolored bars are not presented in the top 20 after INKA. Network plots of measured kinases, substrates and inferred kinases in INKA analysis before and after treatment. Blue lines indicate evidence by NWK, orange by PSP and green by both.


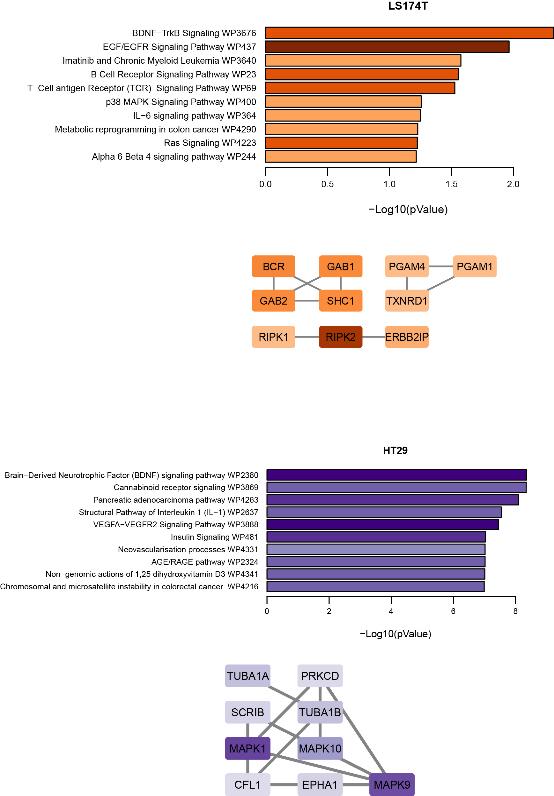


**
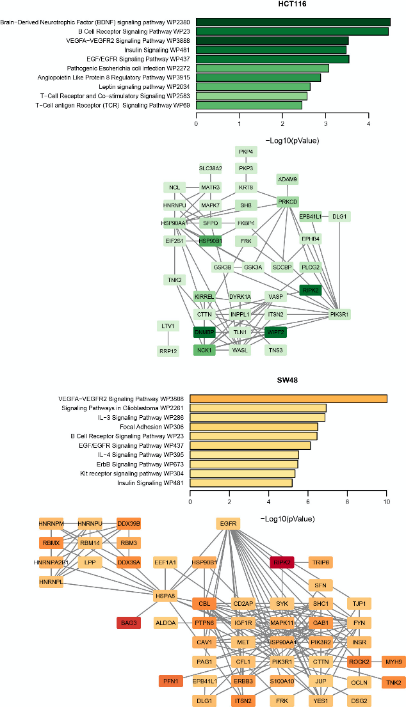
**

## Supplementary Figure S20. Pathway enrichment analysis

Pathway enrichment analysis (WikiPathways) of downregulated phosphoproteins in LS174T (n=23), HT29 (n=14), HCT116 (n=49) and SW48 (n=70). The selection of phosphogenes was based on the sum of normalized spectral counts > 5 and fold change >1.5 in both replicates. The top 10 functional clusters are sorted according to p-value and color intensity is proportional to the number of represented genes per pathway. Bottom panels: protein interaction analysis of downregulated phosphoproteins using STRING (string-db.org). Nodes are color-coded proportional to fold-change. Unconnected nodes are left out of the presentation.


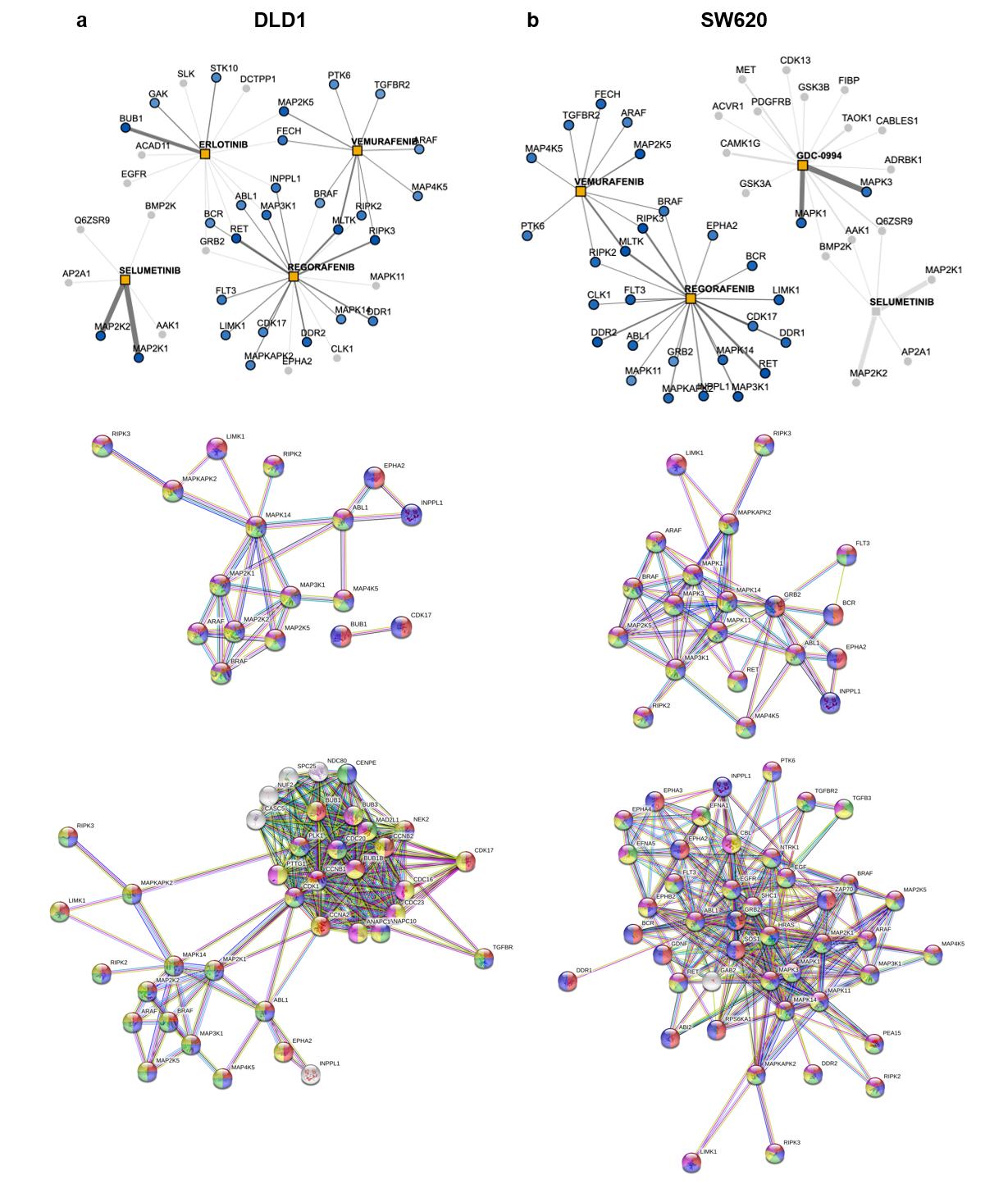

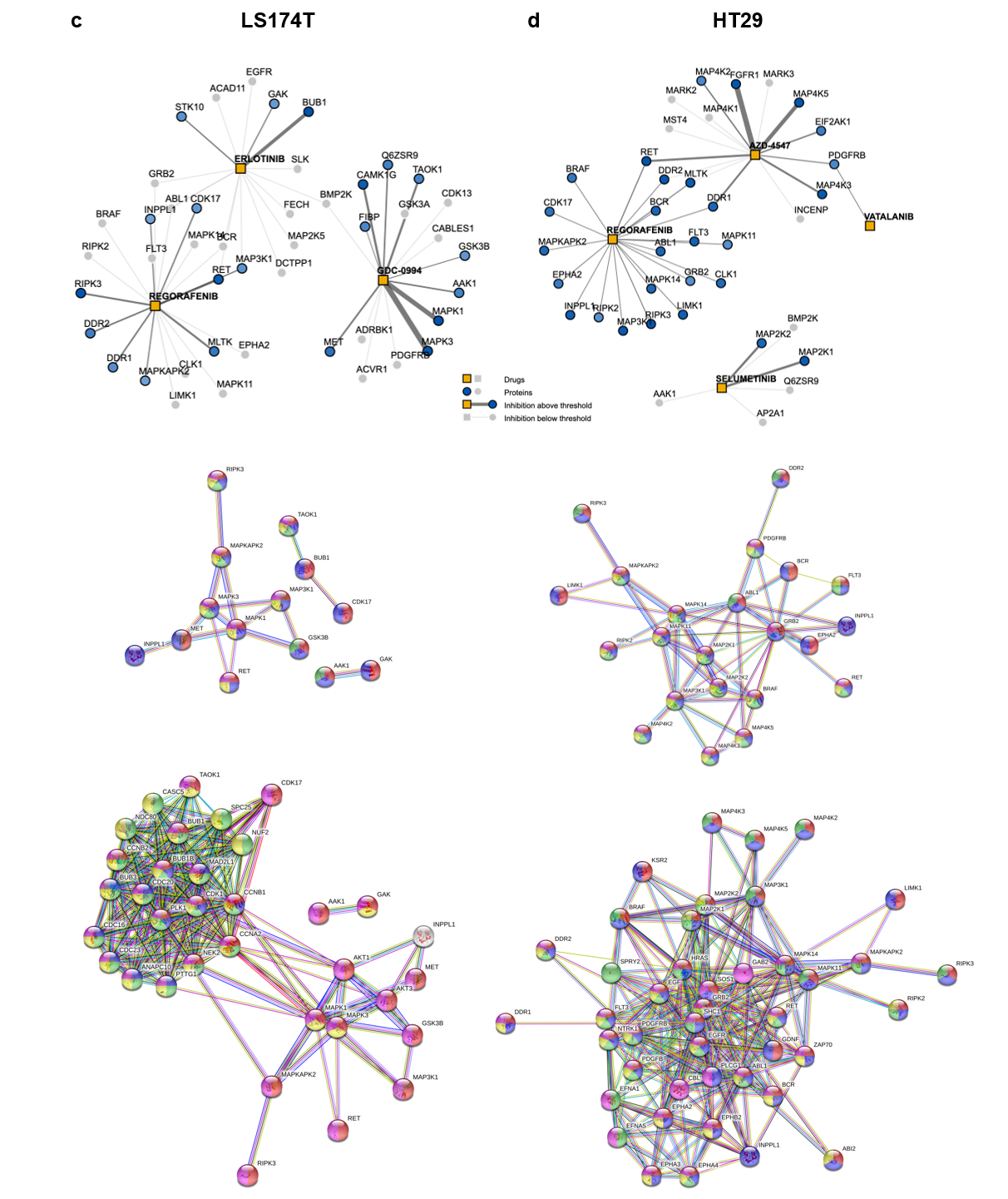

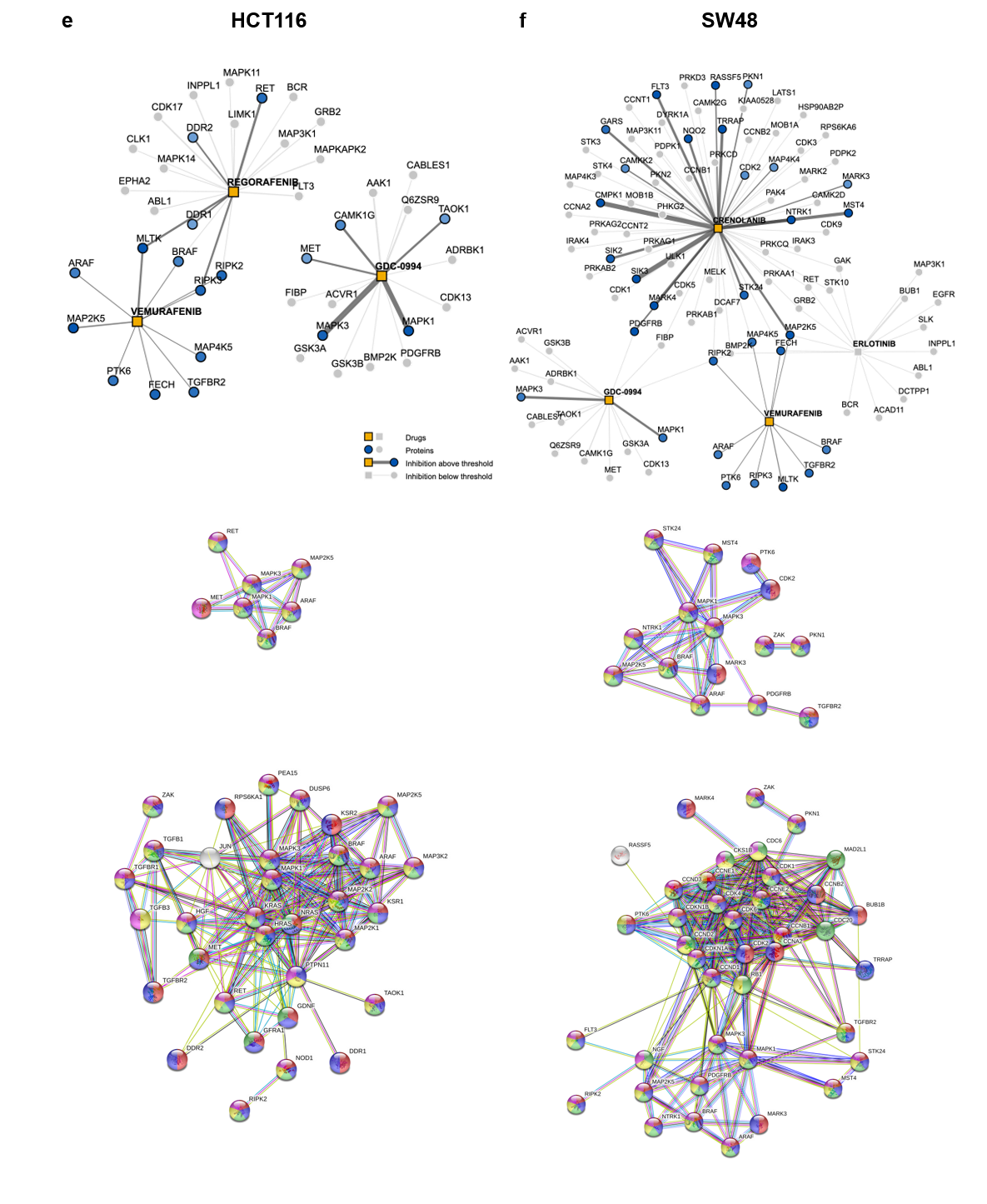


## Supplementary Figure S21. *In silico* analysis of ODCs target proteins in CRC cells

**a.** DLD1, **b.** SW620, **c.** LS174T, **d.** HT29, **e.** HCT116 and **f.** SW48. ODCs target proteins with effective inhibition (EI%) of >50% (top graphs) (<https://www.proteomicsdb.org>). Drugs are marked in orange boxes and proteins in blue. Note that only some inhibition was above the threshold (thick line) at applied drug doses. The target proteins were subject to network analysis using STRING (middle graphs) and enrichment for the GO database. The top targets are sorted according to p-value and color intensity is proportional to the number of represented targets per ontology. An enhanced network up to 10 interactors per 1^st^ and 2^nd^ order shell was performed. GO-Reactome pathways database indicated the top four enriched pathways were enriched significantly: **a**. HSA-69278 (cell cycle, mitotic), HSA-69620 (cell cycle checkpoints), HSA-69618 (mitotic spindle checkpoint), HSA-68886 (M phase); **b.** HSA-422475 (axon guidance), HSA-9006934 (signaling by receptor tyrosine kinases), HSA-187037 signaling by NTRK1, HSA-5673001 (RAF/MAP kinase cascade); **c.** HSA-422475 (axon guidance), HSA-9006934 (signaling by receptor tyrosine kinases), HSA-5673001 (RAF/MAPK kinase cascade), HSA-5663202 (diseases of signal transduction); **d.** HSA-69278 (cell cycle, mitotic), HSA-68886 (M phase), HSA-69620 (cell cycle checkpoints), HSA-69618 (mitotic spindle checkpoint); **e.** HSA-5683057 (MAPK family signaling cascades), HSA-5684996 (MAPK1/MAPK3 signaling), HSA-5673001 (RAF/MAPK kinase cascade); **f.** HSA-453279 (mitotic G1-G1/S phases), HSA-69278 (cell cycle, mitotic), HSA-69206 (G1/S transition), HSA-69231 (cyclin D associated events in G1).


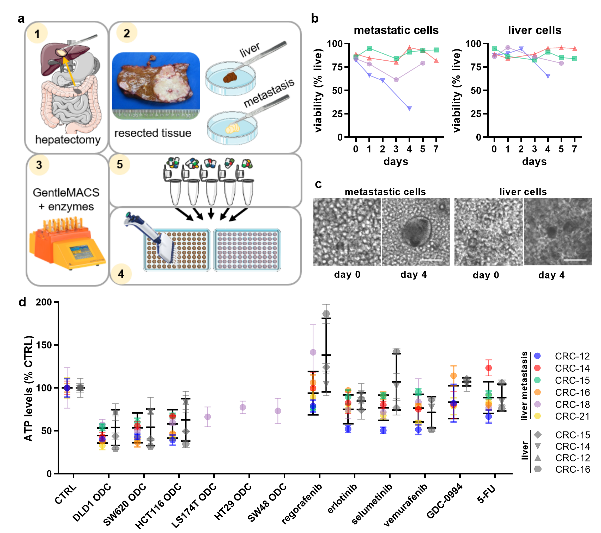


## Supplementary Figure S22. Cell-specific ODC activity in patient liver metastasis and normal liver cells

**a.** Schematic representation of (1) hepatectomy for metastasis resection, (2) mechanical and (3) GentleMACS and enzymatic digestion of the tissue with final (4) seeding of cell suspensions and (5) drug (combination) treatment. **b.** Cell viability determined with live/dead fluorescence staining for four representative cultures of patient metastasis and normal liver cells. **c.** Representative images of cells isolated from one tumor (CRC12). Scalebar = 50 µm. **c.** Intra-patient heterogeneous treatment response in cell cultures isolated from six patients to various cell-specific ODCs.

### **Supplementary Information S1:** Therapeutically Guided Multidrug Optimization method


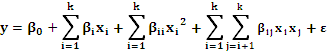

Supplement: Supplementary file 1 — Fig. S1. Graphical representation of the TGMO method and study approach. Fig. S2. Analysis of linear regression models. Fig. S3. Drug dose‐response curves for each cell line in the TGMO screen. Fig. S4. TGMO identifies DLD1‐specific ODC. Fig. S5. TGMO identifies SW620‐specific ODC. Fig. S6. TGMO identifies LS174T‐specific ODC. Fig. S7. TGMO identifies HT29‐specific ODC. Fig. S8. TGMO identifies HCT116‐specific ODC. Fig. S9. TGMO identifies SW48‐specific ODC. Fig. S10. ODC response surfaces and synergistic power distribution. Fig. S11. Cell cycle distribution after ODC treatment at 24 and 72 hours. Fig. S12. Cell morphology after ODC treatment. Fig. S13. ODC validation in heterotypic 3D cultures with fibroblasts and endothelial cells Fig. S14. Tumor growth curves after single drug treatments in DLD1 and SW620 tumors in vivo. Fig. S15. DLD1‐specific ODC is effective and non‐toxic in vivo and accumulates in the blood. Fig. S16. Analysis of morphology, proliferating cells and microvessel density in DLD1 and SW620 tumors. Fig. S17. Differential gene expression analysis in HT29 and LS174T cells. Fig. S18. Differential gene expression analysis in HCT116 and SW48 cells. Fig. S19. INKA profiles and networks before after ODCs treatments in CRC cell lines. Fig. S20. Pathway enrichment analysis. Fig. S21. In silico analysis of ODCs target proteins in CRC cells. Fig. S22. Cell‐specific ODC activity in patient liver metastasis and normal liver cells. [file MOL2-14-2894-s001.docx]
