# Supplementary material for: Optimized low‐dose combinatorial drug treatment boosts selectivity and efficacy of colorectal carcinoma treatment
Source: Mol Oncol. 2020 Oct 5;14(11):2894–919. doi: 10.1002/1878-0261.12797 (PMC7607171; doi:10.1002/1878-0261.12797)
Supplement: Supplementary file 2 — Table S1. The panel of CRC cell lines used in 3D cultures. Table S2. Selected drugs, drug targets and clinical status. Table S3. Drug plasma concentration limit (PCL) calculation table. Table S4. Cell line‐specific drug doses of the ODCs in different optimization phases. Table S5. Combination index of ODC activity from Search and final dose optimization. Table S6. Cross‐validation of the cell‐specific ODCs across the panel of CRC cells. Table S7. Single drug efficacy in DLD1 tumors in vivo. Table S8. Single drug efficacy in SW620 tumors in vivo. Table S9. Growth of DLD1 and SW620 sham‐treated tumors in vivo. Table S10. Pharmacokinetics of DLD1‐specific ODC and corresponding single drugs. Table S11. Pharmacokinetics of SW620‐specific ODC and corresponding single drugs. Table S12. Intratumor and serum mean drug concentrations of DLD1 and SW620 tumor models at the experimental endpoint. Table S13. Top 50 downregulated gene expression of ODC‐treated cells. Table S14. Lysate protein. Table S15. Lysate peptide. Table S16. Sample labeling. Table S17. pTyrIP Phosphosite. Table S18. pTyrIP Phosphopeptide. Table S19. Normalized spectral counts of phosphokinases. [file MOL2-14-2894-s002.docx]

# Supplementary Tables

**Optimized low-dose combinatorial drug treatment boosts selectivity and efficacy of colorectal carcinoma treatment**

Marloes Zoetemelk^1,2,3^, George M. Ramzy^1,2,3^, Magdalena Rausch^1,2,3^, Thibaud Koessler^4^,

Judy R. van Beijnum^5^, Andrea Weiss^1,2^, Valentin Mieville^1,2^, Sander R. Piersma^6,7^, Richard R. de Haas^6,7^, Céline Delucinge-Vivier^8^, Axel Andres^10,11^, Christian Toso^10,11^, Alexander A. Henneman^6,7^, Simone Ragusa^12,13^, Tatiana V. Petrova^12,13^, Mylène Docquier^8,9^, Thomas A. McKee^14^, Connie R. Jimenez^6,7^, Youssef Daali^15^, Arjan W. Griffioen^5^, Laura Rubbia-Brandt^14^, Pierre-Yves Dietrich^3,4^,

Patrycja Nowak-Sliwinska^1,2,3^*

^1^ Molecular Pharmacology Group, School of Pharmaceutical Sciences, University of Geneva, Geneva, Switzerland

^2^ Institute of Pharmaceutical Sciences of Western Switzerland, University of Geneva, Geneva, Switzerland

^3^ Translational Research Center in Oncohaematology, Geneva, Switzerland

^4^ Department of Oncology, Geneva University Hospitals and Faculty of Medicine, Geneva, Switzerland

^5^ Angiogenesis Laboratory, Department of Medical Oncology, Cancer Center Amsterdam, Amsterdam UMC-location VUmc, VU University Amsterdam, Amsterdam, The Netherlands

^6^ Department of Medical Oncology, Cancer Center Amsterdam, Amsterdam UMC, Vrije Universiteit Amsterdam, Amsterdam, The Netherlands

^7^ OncoProteomics Laboratory, Cancer Center Amsterdam, Amsterdam UMC, Vrije Universiteit Amsterdam, Amsterdam, The Netherlands

^8^ iGE3 Genomics Platform, University of Geneva, Switzerland

^9^ Department of Genetics & Evolution, University of Geneva, Switzerland

^10^ Translational Department of Digestive and Transplant Surgery, Geneva University Hospitals and Faculty of Medicine, Geneva, Switzerland

^11^ Hepato-Pancreato-Biliary Centre, Geneva University Hospitals and Faculty of Medicine, Geneva, Switzerland

^12^ Department of Oncology, University of Lausanne, Lausanne, Switzerland

^13^ Ludwig Institute for Cancer Research Lausanne, Lausanne, Switzerland

^14^ Division of Clinical Pathology, Diagnostic Department, University Hospitals of Geneva (HUG), Geneva, Switzerland

^15^ Division of Clinical Pharmacology and Toxicology, Department of Anaesthesiology, Pharmacology, Intensive Care and Emergency Medicine, Geneva University Hospitals, Geneva, Switzerland

## Supplementary Table S1. The panel of CRC cell lines used in 3D cultures

| *Cell line* | *Origin* | *Duke’s type* | *MSI/CIN status* | *Mutations/ deregulations* | *Doubling time in 2D (h)* | *Reference* |
| --- | --- | --- | --- | --- | --- | --- |
| DLD1 | Primary | C | MSI (CMS1) | APC^l1417fs,R2166^ KRAS^G13D^ PIK3CA^E545K;D549N^ TP53^S241F^ | 20 | [1-4] |
| SW620 | Metas-tatic | C | MSS; CIN^pos46^  (CMS4) | APC^Q1338^ KRAS^G12V^ TP53^R273H;P309S^ | 31 | [3-6] |
| LS174T | Primary | C | MSI (CMS3) | KRAS^G12D^ PIK3CA^H1047R^ | 32 | [3, 4, 6, 7] |
| HT29 | Primary | C | MSS (CMS3) | APC^E853;T1556fs^ BRAF^V600E^ PIK3CA^P449T^ TP53^R273H^ | 20-24 | [3, 8] |
| HCT116 | Primary | A | MSI (CMS4) | KRAS^G13D^ PIK3CA^H1047R^ | 20-24 | [3, 4, 6, 9, 10] |
| SW48 | Primary | C | MSI (CMS1) | EGFR^G719S^ | 31 | [3, 5, 11] |

MSI: microsatellite instability; MSS: microsatellite stability; CIN: chromosomal instability pathway; CMS1: consensus MSI immune; CMS2: consensus MSI canonical; CMS3: consensus MSI metabolic; CMS4: consensus MSI mesenchymal

## Supplementary Table S2. Selected drugs, drug targets and clinical status

| *Drug name* | *Drug target* | *Status* |
| --- | --- | --- |
| regorafenib | VEGFR1-3, PDGFRβ, FGFR1, TIE2, c-KIT, and RET | approved |
| erlotinib | EGFR | approved |
| selumetinib | MEK1,2 | phase II/III |
| BEZ-235 | PI3K, mTOR | phase II |
| vatalanib | VEGFR1-3 | phase II/III |
| zaltrap | VEGF | approved |
| vemurafenib | BRAF | approved |
| vorinostat | HDAC | approved |
| AZD-4547 | FGFR | phase II/III |
| crenolanib | PDGFRα,β, FLT2 | Phase II/III |
| GDC-0994 | ERK1/2 | Phase I |

## Supplementary Table S3. Drug plasma concentration limit (PCL) calculation table

| *Drug name* | *PCL (µM)* | *Clinical dose* | *Schedule** | *AUC** and reference* |
| --- | --- | --- | --- | --- |
| regorafenib | 4.34 | 160 mg/kg | 3 wk on/  1 wk off | 50.26 mg*h/L [12]  58.3 mg*h/L [13] |
| erlotinib | 1.61 | 150 mg/kg | once daily | 15.2 mg*h/L EPAR WC00033991 |
| selumetinib | 0.60 | 100 mg/kg  150 mg/kg | twice daily | 3.299_(0-12h)_ µg*h/L [14]  2.999_(0-12h)_ µg*h/L [15] |
| BEZ-235 | 0.09 | 400 mg/kg | once daily | 741.3 µg*h/L [16];  1404.4 µg*h/L [17] |
| vatalanib | 8.31 | 1,200 mg/kg  1,000 mg/kg | once daily | 69.2 mg*h/L [18]  52.9 mg*h/L [19] |
| zaltrap | 0.12 | 4 mg/kg | i.v. once/ 2 wk | 293 mg*h/L [20]  218 mg*d/L EPAR 755420 |
| vemurafenib | 11.1 | 960 mg/kg | twice daily | 131 mg*h/L [21]  21.1 mg*h/L EPAR 002409 |
| vorinostat | 0.19 | 200 mg/kg | twice daily | 1.184 µg*h/L [22]  4.61 h*µM [23] |
| AZD-4547 | 0.37 | 120 mg/kg | twice daily | 2058_(0-12h)_ µg*h/L [24] |
| crenolanib | 0.43 | 200 mg/kg  100 mg/kg | once daily  twice daily | 4.610 mg*h/L [25]  3.2 mg*h/L [26] |
| GDC-0994 | 2.02 | 200 mg/day | 3 wk on/1 wk off | AUC estimated from data [27] |

* Schedule of drug administrated, route of administration is oral unless otherwise specified.
** AUC from 0-24h unless otherwise specified.

## Supplementary Table S4. Cell-line specific drug doses of the ODCs in different optimization phases

| *Cell line* | | *Phase* | *PCL* | *DLD1* | *SW620* | *LS174T* | *HT29* | *HCT116* | *SW48* |
| --- | --- | --- | --- | --- | --- | --- | --- | --- | --- |
| regorafenib (µM) | | *Search 3*  *Search 4* | 4.3 | 2  2 | 3  4 | 1  1 | 3  4 | 0.4  0.6 |  |
| erlotinib (µM) | | *Search 3*  *Search 4* | 1.6 | 1.6  1.6 |  | 0.8  1.2 |  |  | 0.2  0.1 |
| selumetinib (µM) | | *Search 3*  *Search 4* | 0.6 | 0.6  0.6 | 0.01  0.02 |  | 0.2  0.3 |  |  |
| BEZ-235 (µM) | | *Search 3*  *Search 4* | 1.7 |  |  |  |  |  |  |
| vatalanib (µM) | | *Search 3*  *Search 4* | 8.3 |  |  |  | 8  8 |  |  |
| zaltrap (µM) | | *Search 3*  *Search 4* | 0.1 |  |  |  |  |  |  |
| vemurafenib (µM) | | *Search 3*  *Search 4* | 11.1 | 2  5 | 10  9 |  |  | 10  10 | 11  11 |
| vorinostat (µM) | | *Search 3*  *Search 4* | 0.2 |  |  |  |  |  |  |
| AZD-4547 (µM) | | *Search 3*  *Search 4* | 0.4 |  |  |  | 3  0.4 |  |  |
| crenolanib (µM) | | *Search 3*  *Search 4* | 0.4 |  |  |  |  |  | 0.4  0.4 |
| GDC-0994 (µM) | | *Search 3*  *Search 4* | 2.0 |  | 0.3  0.2 | 1  2 |  | 0.2  0.5 | 0.04  0.12 |
| CRC | viability (% CTRL)  SD (% CTRL)  viability (% CTRL)  SD (% CTRL) | *Search 3*  *Search 4* |  | **37.4**  2.9  **34.2**  5.3 | **34.7**  3.5  **33.5**  2.9 | **39.8**  1,5  **36.2**  8.4 | **32.5**  4.3  **28.2**  4.9 | **45.8**  9.2  **30.3**  4.3 | **53.7**  4.0  **42.4**  8.6 |
| TW | viability (% CTRL)  SD (% CTRL)  viability (% CTRL)  SD (% CTRL) | *Search 3*  *Search 4* |  | **64.7**  5.7  **61.5**  7.3 | **45.8**  3.3  **63.6**  10.9 | **79.2**  3.5  **78.6**  6.4 | **63.3**  2.5  **72.1**  6.3 | **59.8**  7.1  **62.3**  5.7 | **59.5**  2.8  **75.2**  6.8 |

## Supplementary Table S5. Combination index of ODC activity from *Search 3* and final dose optimization.

|  |  | *DLD1* | | *SW620* | *HCT116* | *LS174T* | *HT29* | *SW48* |
| --- | --- | --- | --- | --- | --- | --- | --- | --- |
| TGMO interactions | Synergisms | reg:sel | reg:vem | | reg:vem vem:GDC | reg:GDC | reg:AZD  vat:AZD | vem: cre |
| TGMO interactions | Additivities | reg, erl, sel, vem | reg, sel, GDC | | reg, vem, GDC | reg, erl, GDC | reg, sel, AZD | reg, erl, GDC |
| CI Loewe *Search 3* | CRC cells  healthy cells | 0.63  1.11 | 0.64  1.13 | | 0.55  1.02 | 0.76  1.63 | 0.89  0.32 | 9.89  0.69 |
| CI Loewe *Search 4* | CRC cells  healthy cells | 0.67  1.00 | 0.44  1.29 | | 0.57  1.10 | 0.80  1.45 | 1.18  0.37 | 24.67  1.24 |

Drugs: reg, regorafenib; erl, erlotinib; sel, selumetinib; vem, vemurafenib, GDC, GDC-0994; vat, vatalanib; AZD, AZD-4547; cre, crenolanib. CI < 1 = synergistic combination, CI > 1 = antagonistic combination.

## Supplementary Table S6. Cross-validation of the cell-specific ODCs across the panel of CRC cells.

| *Cell line* |  | *DLD1* | *SW620* | *LS174T* | *HT29* | *HCT116* | *SW48* |
| --- | --- | --- | --- | --- | --- | --- | --- |
| DLD1 | viability (% CTRL) | **34.2** | **27.2** | **47.6** | **54.3** | **39.2** | **76.8** |
|  | SD (% CTRL) | 3.0 | 9.0 | 3.3 | 26.0 | 10.8 | 18.8 |
| SW620 | viability (% CTRL) | **29.5** | **33.5** | **53.4** | **47.0** | **38.6** | **124.0** |
|  | SD (% CTRL) | 8.6 | 2.9 | 28.5 | 23.8 | 3.0 | 10.3 |
| LS174T | viability (% CTRL) | **26.6** | **27.4** | **36.2** | **29.6** | **52.9** | **89.3** |
|  | SD (% CTRL) | 5.5 | 9.0 | 8.4 | 14.8 | 11.7 | 2.8 |
| HT29 | viability (% CTRL) | **29.3** | **35.2** | **35.8** | **28.2** | **48.7** | **40.5** |
|  | SD (% CTRL) | 17.6 | 7.4 | 6.4 | 4.9 | 15.5 | 14.7 |
| HCT116 | viability (% CTRL) | **25.7** | **22.4** | **33.1** | **26.9** | **30.3** | **52.9** |
|  | SD (% CTRL) | 2.7 | 2.1 | 8.5 | 9.9 | 4.3 | 12.8 |
| SW48 | viability (% CTRL) | **28.7** | **50.3** | **28.5** | **48.8** | **49.6** | **42.4** |
|  | SD (% CTRL) | 8.0 | 8.5 | 7.5 | 7.8 | 8.0 | 8.6 |

## Supplementary Table S7. Single drug efficacy in DLD1 tumors *in vivo*

|  | *regorafenib* | | | | | *selumetinib* | | | | | | | | |
| --- | --- | --- | --- | --- | --- | --- | --- | --- | --- | --- | --- | --- | --- | --- |
|  | *15* | *15* | *30* | *30* | *50* | *1* | 5 | 5 | 10 | 10 | | 25 | | 50 |
| % CTRL  % SD  % SEM | 87  75  34 | 96  24  38 | 85  46  20 | 63  56  25 | 135  76 29 | 74  29  12 | 37  39  20 | 67  50  25 | 82  49  22 | 33  18  8 | | 49  33  13 | | 39  16  7 |
| Volume  SD  SEM | 559  479  214 | 120  78  39 | 594  320  143 | 404  358  160 | 465 261 99 | 366  142  58 | 237  234  125 | 408  223  79 | 457  270  121 | 213  117  52 | | 168  112  46 | | 272  115  52 |
| n | 5 | 7 | 5 | 5 | 7 | 6 | 3 | 8 | 5 | 5 | | 7 | | 5 |
| Exp  Day | 5  16 | 7  15 | 2  14 | 5  16 | 3 13 | 6  18 | 5  16 | 7  25 | 4  16 | 5  16 | | 3  13 | | 2  14 |
|  | *vemurafenib* | | | | | | *erlotinib* | | | |  | |  | |
|  | 25 | 75 | 75* | 75 | 75 | 150* | *5* | *12.5* | *12.5* | *12.5* | |  | |  |
| % CTRL  % SD  % SEM | 80  33  19 | 87  46  23 | 113  88  39 | 94  31  14 | 123  69  24 | 105  64  28 | 104  72  27 | 87  35  13 | 66  28  12 | 98  44  22 | |  | |  |
| Volume  SD  SEM | 562  231  133 | 612  319  160 | 388  304  136 | 600  200  89 | 619  347  123 | 587  354  159 | 482  336  127 | 405  161  61 | 420  178  80 | 420  211  86 | |  | |  |
| n | 5 | 5 | 6 | 5 | 8 | 5 | 7 | 7 | 5 | 6 | |  | |  |
| Exp  Day | 2  14 | 2  14 | 3  13 | 5  16 | 7  15 | 4  16 | 1  11 | 1  11 | 5  16 | 7  15 | |  | |  |

* b.i.d.

## Supplementary Table S8. Single drug efficacy in SW620 tumors *in vivo*

|  | *regorafenib* | | | *selumetinib* | | | | *vemurafenib* | | *GDC-0994* | | |
| --- | --- | --- | --- | --- | --- | --- | --- | --- | --- | --- | --- | --- |
| *Reg + Erl* | *30* | *30* | *30* | *0.2* | *0.2* | *1* | *10* | *75* | *75* | *20* | *10* | *10* |
| % CTRL  % SD  % SEM | 117  45  20 | 90  78  35 | 44  18  10 | 76  41  21 | 93  5  3 | 67  55  24 | 43  20  9 | 65  42  21 | 94  48  58 | 62  31  22 | 56  33  15 | 56  18  7 |
| Volume  SD  SEM | 567  220  98 | 468  407  182 | 278  111  64 | 393  214  107 | 588  30  21 | 323  265  118 | 209  95  43 | 337  219  109 | 936  488  365 | 301  148  105 | 290  170  76 | 354  114  46 |
| n | 5 | 5 | 3 | 4 | 2 | 5 | 5 | 4 | 3 | 2 | 5 | 6 |
| Exp,  Day | 4  22 | 6  20 | 8  19 | 6  20 | 8  19 | 4  22 | 4 22 | 6  20 | 8  19 | 4  22 | 6  20 | 8  19 |

## Supplementary Table S9. Growth of DLD1 and SW620 sham-treated tumors *in vivo*

| *CTRL* | *DLD1* | *DLD1* | *DLD1* | *DLD1* | *DLD1* | *DLD1* | *DLD1* | *SW620* | *SW620* | *SW620* |
| --- | --- | --- | --- | --- | --- | --- | --- | --- | --- | --- |
| % CTRL  % SD  % SEM | 100  50  20 | 100  44  22 | 100  65  27 | 100  53  26 | 100  65  25 | 100  42  19 | 100  59  24 | 100  57  29 | 100  44  22 | 100  62  36 |
| Volume  SD  SEM | 466  233  95 | 702  310  155 | 345  224  91 | 557  295  147 | 639  418  158 | 498  210  94 | 504  297  121 | 485  277  138 | 519  230  115 | 633  389  225 |
| n | 6 | 4 | 6 | 4 | 7 | 5 | 6 | 5 | 4 | 3 |
| Exp  Day | 1  11 | 2  14 | 3  13 | 4  16 | 5  16 | 6  18 | 7  15 | 4  22 | 6  20 | 8  19 |

Mice n and weight correspond to day 1 at the start of the experiment. All data is provided as the mean ± standard deviation.

## Supplementary Table S10. Pharmacokinetics of DLD1-specific ODC and corresponding single drugs

| *Cell line* | *regorafenib  15 mg/kg* | | *erlotinib  12.5 mg/kg* | | *selumetinib  5 mg/kg* | | *vemurafenib*  *75 mg/kg* | |
| --- | --- | --- | --- | --- | --- | --- | --- | --- |
|  | *mono* | *ODC* | *mono* | *ODC* | *mono* | *ODC* | *mono* | *ODC* |
| n total  male  female | 3  1  2 | 4  2  2 | 4  2  2 | 4  2  2 | 4  2  2 | 4  2  2 | 4  2  2 | 4  2  2 |
| Mean weight (g)  male  female | 31.0  23.5 | 29.5 25.0 | 30.0  25.0 | 29.5 25.0 | 34.5 27.5 | 29.5 25.0 | 30.5 28.0 | 29.5 25.0 |
| AUC_0-24h_ (µg*h/mL) | **17.54** ± 5.79 | **49.41** ± 2.98 | **19.58** ± 7.66 | **22.28** ± 5.14 | **15.16** ± 7.59 | **24.83** ± 8.25 | **2.18**  ± 0.22 | **14.43**  ± 2.74 |
| C_max_  (µg*h/mL) | 1.19 ± 0.69 | 3.38 ± 0.49 | 2.61 ± 0.86 | 3.37 ± 2.16 | 1.22 ± 0.33 | 3.83 ± 1.64 | 0.27 ± 0.08 | 1.05 ± 0.36 |
| T_max_ (h) | 8 | 4 | 2 | 2 | 2 | 2 | 2 | 4 |

Mice n and weight correspond to day 1 at the start of the experiment. All data is provided as the mean ± standard deviation.

## Supplementary Table S11. Pharmacokinetics of SW620-specific ODC and corresponding single drugs

| *Cell line* | *regorafenib  30 mg/kg* | | *selumetinib 0.02 mg/kg* | | *vemurafenib  75 mg/kg* | | *GDC-0994*  *10 mg/kg* | |
| --- | --- | --- | --- | --- | --- | --- | --- | --- |
|  | *mono* | *ODC* | *mono* | *ODC* | *mono* | *ODC* | *mono* | *ODC* |
| n total  male  female | 4  2  2 | 4  2  2 | 4  2  2 | 4  2  2 | 4  2  2 | 4  2  2 | 4  2  2 | 4  2  2 |
| Mean weight (g)  male  female | 32.5  22.5 | 29.0  22.5 | 28.0 23.0 | 29.0  22.5 | 32.0  23.0 | 29.0  22.5 | 31.0  24.0 | 29.0  22.5 |
| AUC_0-24h_ (µg*h/mL) | **9.86** ± 1.93 | **22.18** ±1.83 | **0.77** ± 0.14 | **0.89** ± 0.13 | **19.43** ± 3.25 | **48.20** ± 5.72 | **31.61**  ± 4.74 | **45.00** ± 9.09 |
| C_max_  (µg*h/mL) | 0.71 ± 0.26 | 1.61 ± 0.22 | 0.24 ± 0.08 | 0.23 ± 0.08 | 2.56 ± 0.64 | 4.71 ± 0.65 | 6.09 ± 1.93 | 6.61 ± 1.11 |
| T_max_ (h) | 4 | 4 | 2 | 2 | 2 | 4 | 2 | 2 |

Mice n and weight correspond to day 1 at the start of the experiment. All data is provided as the mean ± standard deviation.

## Supplementary Table S12. Intra-tumor and serum mean drug concentrations of DLD1 and SW620 tumor models at the experimental endpoint

|  | *concentration  serum (µg/mL)* | | *concentration  tumor (µg/g)* | | *ratio tumor vs serum* | | *ratio  ODC vs single drug* | |
| --- | --- | --- | --- | --- | --- | --- | --- | --- |
| *DLD1* | *single dr.* | *ODC* | *single dr.* | *ODC* | *single dr.* | *ODC* | *serum* | *tumor* |
| regorafenib 15 mg/kg | 0.88 ±0.25 | 1.83 ±0.54 | 1.54 ±0.68 | 8.67 ±5.53 | 1.76 | 4.74 | 2.09 | 5.62 |
| erlotinib 12.5 mg/kg | 2.33 ±0.18 | 3.04 ±0.91 | 3.62 ±0.14 | 7.60 ±3.20 | 1.55 | 2.50 | 1.31 | 2.10 |
| Selumetinib 5 mg/kg | 2.50 ±1.08 | 3.49 ±0.90 | 1.98 ±0.66 | 4.53 ±1.95 | 0.79 | 1.30 | 1.40 | 2.29 |
| vemurafenib 75 mg/kg | 0.62 | 0.91 ±0.38 | 0.79 | 1.65 ±1.00 | 1.28 | 1.81 | 1.48 | 2.09 |
| SW620 | *single dr.* | *ODC* | *single dr.* | *ODC* | *single dr.* | *ODC* | *serum* | *tumor* |
| regorafenib 30 mg/kg | 2.22 ±0.07 | 6.60 ±2.80 | 1.33 ±0.23 | 1.82 ±0.77 | 0.60 | 0.28 | 2.97 | 1.37 |
| selumetinib 0.2 mg/kg | 0.41 ±0.35 | 0.28 ±0.20 | 0.56 ±0.09 | 0.22 ±0.10 | 1.39 | 0.77 | 0.68 | 0.38 |
| vemurafenib 75 mg/kg | 1.96 ±1.05 | 1.77 ±0.77 | 0.94 0.68 | 0.89 ±0.53 | 0.48 | 0.50 | 0.90 | 0.95 |
| GDC-0994 10 mg/kg | 15.89 ±9.07 | 19.45 ±9.39 | 12.69 ±2.87 | 5.56 ±3.00 | 0.80 | 0.29 | 1.22 | 0.44 |

## Supplementary Table S13. Top 50 downregulated gene expression of ODC-treated cells

| *DLD1* | | | *SW620* | | | *HT29* | | |
| --- | --- | --- | --- | --- | --- | --- | --- | --- |
| *Genes* | *FC>2* | *p-value* | *Genes* | *FC>2* | *p-value* | *Genes* | *FC>2* | *p-value* |
| CSF2  SPRY4  DUSP6  GPR3  DUSP2  TNS4  F3  MYC  RGS16  PHLDA2  CX3CL1  CYP26B1  FJX1  PHLDA1  CCAT1  PLK3  SLC20A1  C3orf52  MYEOV  PLD6  MAFK  MAT2A  NOCT  SPRED1  CLDN2  LRCH1 | -17.6  -15.8  -10.8  -5.3  -5.2  -4.9  -4.6  -4.1  -4.0  -3.1  -2.9  -2.7  -2.7  -2.6  -2.5  -2.4  -2.4  -2.4  -2.3  -2.2  -2.2  -2.2  -2.2  -2.1  -2.0  -2.0 | 0.000193  8.02E-05  4.98E-09  4.02E-05  1.45E-05  5.96E-07  4.95E-09  1.70E-06  4.63E-08  0.000225  7.63E-06  5.97E-06  1.83E-06  0.000249  8.68E-05  0.000101  1.05E-06  2.43E-06  2.17E-05  3.89E-06  1.78E-05  1.11E-06  1.48E-05  0.000155  0.000158  0.000133 | FZD7  IL10  TNS4  F2RL3  PPP2R2C  SLC16A6  DIO3OS  ISX  GPR3  DUSP4  EPHA4  ZSWIM4  DUSP6  KDM7A  PRDM1  RHEBL1  FGF19  ETV5  MGAT3  HAS3  PLK3  DIO3  OGFRP1  SPRED2  SPRED1  WIPI1 | 53.1  -23.5  -22.1  -18.9  -11.7  -9.6  -6.6  -5.4  -5.2  -4.5  -4.0  -4.0  -3.8  -3.7  -3.6  -3.4  -3.3  -3.3  -3.3  -3.2  -3.2  -3.1  -2.9  -2.9  -2.8  -2.7 | 2.86E-05  3.27E-05  2.21E-10  6.48E-05  1.64E-09  2.39E-09  0.000131  5.40E-05  5.78E-05  3.78E-05  6.77E-06  4.02E-06  6.94E-06  1.03E-05  5.78E-06  1.41E-05  1.82E-06  2.86E-08  9.52E-06  2.78E-07  5.73E-06  2.16E-07  4.65E-05  1.01E-05  3.85E-06  1.55E-05 | GPR161  SPRY4  CPS1  DUSP4  FOSL1  EPHA2  ANGPTL2  TNS4  CXCL8  FGF19  HAS3  NR1D1  HTR7P1  SPRED3  ITPRIP  TNFSF15  LIF  HS3ST1  RASSF9  KLF9  SLITRK6  PLK3  RHEBL1  MYC  ZNF792  SGK223 | -73.8  -43.9  -41.4  -15.7  -14.2  -13.4  -13.4  -11.1  -10.2  -9.4  -8.0  -7.9  -7.8  -7.3  -7.0  -6.8  -6.5  -6.4  -5.8  -5.8  -5.7  -5.5  -5.5  -5.4  -5.4  -5.4 | 1.39E-05  5.16E-07  0.000464  6.04E-08  4.83E-05  2.88E-06  0.002578  2.43E-09  6.67E-05  6.07E-08  8.96E-08  7.90E-05  0.001851  7.96E-06  1.10E-10  2.12E-08  4.53E-05  4.33E-08  0.000175  0.001448  4.92E-07  7.96E-08  6.02E-05  1.39E-07  4.30E-10  3.01E-08 |
| LS174T | | | CHST15 | -2.7 | 3.28E-05 | DGAT2 | -5.3 | 4.03E-09 |
| *Genes* | *FC>2* | *p-value* | JAG1 | -2.6 | 3.28E-05 | NIPAL1 | -5.0 | 4.03E-09 |
| CREB5  SERPINB2  TNS4  DUSP4  DKK1  MIR614  IL33  TGFBR2  FOXQ1  RGS16  SMIM3  GPRC5A  IRS1  SPRED1  MYC  SHH  PIM1  SHB | -6.4  -6.4  -4.8  -4.0  -4.0  -3.5  -3.3  -3.2  -3.0  -3.0  -2.9  -2.9  -2.9  -2.7  -2.7  -2.6  -2.6  -2.5 | 4.41E-05  8.13E-05  6.25E-07  8.17E-05  1.23E-05  1.46E-05  5.55E-05  4.93E-06  0.000158  7.23E-07  1.25E-06  2.71E-07  5.29E-05  4.57E-06  7.59E-05  3.10E-05  4.19E-05  3.54E-05 | ASPRV1  BMP4  IL23A  RGL1  LINC00511  RGS16  TLE3  MAFG  DEPDC7  TNFRSF10A  MALT1  HNF4A  CCND1  B3GNT3  ETV1  UBASH3B  MXD1 | -2.6  -2.6  -2.5  -2.5  -2.4  -2.3  -2.3  -2.3  -2.3  -2.2  -2.2  -2.1  -2.1  -2.1  -2.1  -2.1  -2.1  -2.0 | 0.000141  2.39E-07  7.74E-05  8.96E-06  0.000152  8.70E-05  5.51E-08  3.55E-06  1.50E-05  2.25E-06  4.12E-06  2.85E-07  2.40E-05  1.33E-06  0.000144  0.000252  0.000123  0.000224 | IKZF3  PTPN7  SPRED2  KLF10  ZNF485  CXCR4  TXNIP  SUCNR1  CCAT1  CREB5  TXK  MIR614  ERRFI1  DOK7  CCRL2  CXCL1  PRDM8  TLR4 | -5.0  -4.9  -4.9  -4.8  -4.6  -4.6  -4.5  -4.5  -4.5  -4.5  -4.4  -4.4  -4.4  -4.4  -4.4  -4.4  -4.4  -4.2 | 0.000731  0.006785  5.81E-08  2.15E-05  1.84E-06  1.15E-06  0.000833  0.002173  1.18E-07  9.19E-05  2.19E-06  8.52E-06  0.000262  9.62E-05  5.91E-05  0.000484  4.72E-05  3.98E-06 |
| F3 | -2.4 | 1.13E-05 | HCT116 | | | DUSP6 | -4.2 | 4.23E-06 |
| ST3GAL1 | -2.4 | 0.000126 | *Genes* | *FC>2* | *p-value* | HRH1 | -4.2 | 9.28E-06 |
| PLK3  LIPH  KLF4  TNFSF15  SPRY2  MAFK  PDE9A  C3orf52  FFAR4  PAG1  EFNB2  ZFP36L2 | -2.4  -2.3  -2.3  -2.3  -2.3  -2.3  -2.2  -2.2  -2.1  -2.1  -2.1  -2.0 | 0.000156  0.000148  0.000133  7.27E-05  7.32E-06  1.04E-05  8.52E-06  1.00E-05  5.77E-05  1.88E-05  6.49E-05  6.92E-07 | HAS3  SOX9  F3  DNMBP  TSC22D1 | -3.9  -2.9  -2.5  -2.4  -2.1 | 4.86E-08  5.33E-06  3.56E-06  1.20E-06  1.07E-06 | MYEOV  DUSP2 | -4.1  -4.1 | 1.56E-08  5.42E-05 |

FC, fold change > 2

**Supplementary Table S14: Lysate protein**

**Supplementary Table S15: Lysate peptide**

**Supplementary Table S16: Sample labeling**

**Supplementary Table S17: pTyrIP Phosphosite**

**Supplementary Table S18: pTyrIP Phosphopeptide**

**Supplementary Table S19: Normalized spectral counts of phosphokinases**

**Supplementary Tables S14-S19** have been deposited at Zenodo with DOI: 10.5281/zenodo.3580018

# References Supplementary Tables

Uncategorized References

1. Chen, T.R., et al. (1995). DLD-1 and HCT-15 cell lines derived separately from colorectal carcinomas have totally different chromosome changes but the same genetic origin. Cancer Genet Cytogenet *81*, 103-8.

2. Dexter, D.L., et al. (1981). Heterogeneity of cancer cells from a single human colon carcinoma. Am J Med *71*, 949-56.

3. Ahmed, D., et al. (2013). Epigenetic and genetic features of 24 colon cancer cell lines. Oncogenesis *2*, e71.

4. Karagiannis, G.S., et al. (2014). In-depth proteomic delineation of the colorectal cancer exoproteome: Mechanistic insight and identification of potential biomarkers. J Proteomics *103*, 121-36.

5. Leibovitz, A., et al. (1976). Classification of human colorectal adenocarcinoma cell lines. Cancer Res *36*, 4562-9.

6. Morelli, M.P., et al. (2012). Preclinical Activity of the Rational Combination of Selumetinib (AZD6244) in Combination with Vorinostat in KRAS-Mutant Colorectal Cancer Models. Clinical cancer research : an official journal of the American Association for Cancer Research *18*, 10.1158/1078-0432.CCR-11-1507.

7. Tom, B.H., et al. (1976). Human colonic adenocarcinoma cells. I. Establishment and description of a new line. In Vitro *12*, 180-91.

8. J., F. (1975). New human tumor cell lines. In: Fogh J. (eds) Human tumor cells in vitro. Springer, Boston, MA.

9. Brattain, M.G., et al. (1981). Initiation and characterization of cultures of human colonic carcinoma with different biological characteristics utilizing feeder layers of confluent fibroblasts. Oncodev Biol Med *2*, 355-66.

10. Brattain, M.G., et al. (1981). Heterogeneity of malignant cells from a human colonic carcinoma. Cancer Res *41*, 1751-6.

11. Ovechkina, Y.Y. (2008). Assessing cancer therapeutic agents across a fifteen human tumor cell line panel. Cancer research *68*, 1550.

12. Strumberg, D., et al. (2012). Regorafenib (BAY 73-4506) in advanced colorectal cancer: a phase I study. Br J Cancer *106*, 1722-7.

13. Eisen, T., et al. (2012). Regorafenib for patients with previously untreated metastatic or unresectable renal-cell carcinoma: a single-group phase 2 trial. Lancet Oncol *13*, 1055-62.

14. O'Neil, B.H., et al. (2011). Phase II study of the mitogen-activated protein kinase 1/2 inhibitor selumetinib in patients with advanced hepatocellular carcinoma. J Clin Oncol *29*, 2350-6.

15. LoRusso, P.M., et al. (2017). A phase I dose-escalation study of selumetinib in combination with docetaxel or dacarbazine in patients with advanced solid tumors. BMC Cancer *17*, 173.

16. Wise-Draper, T.M., et al. (2017). A Phase Ib Study of the Dual PI3K/mTOR Inhibitor Dactolisib (BEZ235) Combined with Everolimus in Patients with Advanced Solid Malignancies. Target Oncol *12*, 323-332.

17. Bendell, J.C., et al. (2015). A phase 1 study of the sachet formulation of the oral dual PI3K/mTOR inhibitor BEZ235 given twice daily (BID) in patients with advanced solid tumors. Invest New Drugs *33*, 463-71.

18. Mross, K., et al. (2005). Phase I clinical and pharmacokinetic study of PTK/ZK, a multiple VEGF receptor inhibitor, in patients with liver metastases from solid tumours. Eur J Cancer *41*, 1291-9.

19. Wang, X., et al. (2014). Vatalanib population pharmacokinetics in patients with myelodysplastic syndrome: CALGB 10105 (Alliance). Br J Clin Pharmacol *78*, 1005-13.

20. Lockhart, A.C., et al. (2010). Phase I study of intravenous vascular endothelial growth factor trap, aflibercept, in patients with advanced solid tumors. J Clin Oncol *28*, 207-14.

21. Grippo, J.F., et al. (2014). A phase I, randomized, open-label study of the multiple-dose pharmacokinetics of vemurafenib in patients with BRAF V600E mutation-positive metastatic melanoma. Cancer Chemother Pharmacol *73*, 103-11.

22. Stathis, A., et al. (2011). Phase I study of decitabine in combination with vorinostat in patients with advanced solid tumors and non-Hodgkin's lymphomas. Clin Cancer Res *17*, 1582-90.

23. Burhenne, J., et al. (2017). Intracellular vorinostat accumulation and its relationship to histone deacetylase activity in soft tissue sarcoma patients. Cancer Chemother Pharmacol *80*, 433-439.

24. Saka, H., et al. (2017). Safety, tolerability and pharmacokinetics of the fibroblast growth factor receptor inhibitor AZD4547 in Japanese patients with advanced solid tumours: a Phase I study. Investigational new drugs *35*, 451-462.

25. Lewis, N.L., et al. (2009). Phase I study of the safety, tolerability, and pharmacokinetics of oral CP-868,596, a highly specific platelet-derived growth factor receptor tyrosine kinase inhibitor in patients with advanced cancers. Journal of clinical oncology : official journal of the American Society of Clinical Oncology *27*, 5262-5269.

26. Michael, M., et al. (2010). Phase Ib study of CP-868,596, a PDGFR inhibitor, combined with docetaxel with or without axitinib, a VEGFR inhibitor. British journal of cancer *103*, 1554-1561.

27. Blake, J.F., et al. (2016). Discovery of (S)-1-(1-(4-Chloro-3-fluorophenyl)-2-hydroxyethyl)-4-(2-((1-methyl-1H-pyrazol-5-y l)amino)pyrimidin-4-yl)pyridin-2(1H)-one (GDC-0994), an Extracellular Signal-Regulated Kinase 1/2 (ERK1/2) Inhibitor in Early Clinical Development. J Med Chem *59*, 5650-60.
